# Atmospheric reaction of methyl mercaptan with hydroxyl radical as an acid rain primary agent

**DOI:** 10.1038/s41598-020-74767-6

**Published:** 2020-10-22

**Authors:** Hamed Douroudgari, Morteza Vahedpour, Samane Mohammadi

**Affiliations:** Department of Chemistry, University of Zanjan, PO Box 38791-45371, Zanjan, Iran

**Keywords:** Atmospheric science, Climate sciences, Chemistry, Theoretical chemistry, Molecular dynamics, Reaction mechanisms, Structure prediction

## Abstract

For the CH_3_SH + OH atmospheric reaction, we study the mechanism, potential energy surface, thermodynamic parameters of all stationary points, and rate of generation of the main product channels at high, low, and intermediate pressures. In this study, the UMP2, UM062X, UB3LYP, and CCSD(T) methods by Dunning and Pople basis sets are used and the results are compared with the experimental data. It is theoretically predicted that the reaction has fourteen possible pathways with eight different products in the gas phase. The thermodynamic results show that OH radical extracts predominantly the hydrogen of the SH functional group compared to the hydrogen of the CH_3_ group of CH_3_SH. Also, the rate constant calculations indicate that the extraction of the hydrogen atom of the SH group has a major role in 150–3000 K, while a good contribution is observed for the hydrogen of methyl group above 1200 K. Our results show that the used methods lead to good agreement with experiment. Finally, we demonstrated that why the main path is the main path.

## Introduction

The chemical formula of methyl sulfide is CH_3_SH. It is named methyl mercaptan and also methanethiol. In the troposphere, the sulfur cycle plays an important role in acid–base chemistry and also in the formation and growth of aerosol particles^1^. The emission of sulfur into the atmosphere occurs through human-made resources and natural cycles. The natural sulfur cycle has been led to forming acid rain and increasing aerosol content throughout the world for centuries^2,3^.

In recent years, the atmospheric sulfur has been a very popular and important research topic due to the need to evaluate the contributions of sulfur compounds in acid rain and climate change. In industrial areas such as East and West European, sulfur compounds mainly are emitted from human activities. Human activities constitute the main part of atmospheric sulfur emissions after natural sources^4^. The main sources of atmospheric sulfur (with related amount) are volcanoes (greater than 5 Tg S a^−1^), sea spray (44 Tg S a^−1^), biogenic emission (98 Tg S a^−1^), coal (69.1 Tg S a^−1^), petroleum products (29.1 Tg S a^−1^), non-ferrous ores (10.7 Tg S a^−1^), and other sources (1.9 Tg S a^−1^)^5^.

In industrial units, S_2_ is applied to produce sulfuric acid for batteries, gunpowder, and heating of rubbers^6^. Also, some of the sulfur compounds are an antifungal agent in the phosphate fertilizer generation process^7^. Sulfur compounds are responsible for short-lived environmental perturbations through the formation of aerosols that cause climate change^8^.

Methyl mercaptan is a volatile gas compound, which releases from natural and industrial sources, including sewage sludge, waste paper, and pulp production. Also, other important resources are buried waste, artificial fuels, activities related to petrochemical fields, and seas^9^.

In the troposphere or stratosphere, methyl mercaptan finally is converted into sulfuric acid in the presence of sulfur dioxide. In the earth’s atmosphere, the presence of sulfate particles along with some radiations leads to the formation of acid rain. Methyl mercaptan in the industry can cause poisoning and even elimination of some catalysts, which their repairs are very difficult^10,11^.

In the atmosphere, generally, organic compounds with thiol functional groups such as RSH–RSR–RSR′, are key environmental issues that are considered as pollutants. Their concentrations in the atmosphere are related to human activities such as petrochemical and synthetic fuel industries.

Recently, it is proved that the oxidation of light methyl sulfide leads to the formation of strong acids such as sulfuric acid and methanosulfonic acid, affecting on acid rain formation^12^.

Dimethylsulphide (dimethylsulfide) and disulfidedimethyl are also more abundant reduced sulfur compounds. They have been released into the atmosphere through biological sources. Methylsulfide and dimethylsulphide have been formed 3–10% of the atmospheric sulfur compounds. It depends on the geographical region^13^.

Reactions of methanethiol with active atmospheric species such as CN, NO_X_ (X = 1–3), and OH radicals have been noticed by experimental and theoretical researchers. Bao-En et al. have investigated the reaction mechanism of CH_3_SH + CN in the gas phase theoretically. This work showed that CN tends to react with the hydrogen of the SH group. Also, this channel was a barrier-less reaction^14^. Reactions of CH_3_SH plus NO and NO_2_ could have a significant role in the atmosphere. The photolysis of CH_3_SH in the presence of NO radicals led to producing CH_3_SNO as an intermediate and (CH3S)_2_ as a final adduc^15^. Also, The reaction of CH_3_SH + NO_2_ led to generate CH_3_SNO intermediate and OH or CH_3_S + HONO and/or CH_3_S(O)H + NO^16^. A theoretical analysis of the kinetics and thermochemistry of the CH_3_SH + NO_2_ reaction was presented by Brudnik et al.^17^. To generate CH_3_S + HNO_2_ and CH_2_SH + HNO_2_ products, they computed rate constants were k(T) = 7.9 × 10^–15^ (T/300)^1.9^ exp(− 8190/T) and k(T) = 7.9 × 10^–13^ (T/300)^1.94^ exp(− 16,290/T) cm^3^ molecule^−1^ s^−1^, respectively. The reaction of methyl mercaptan with NO_3_^18^ had less important in comparison with OH, due to the low rate with an order of 10. Also, NO_3_ reacts predominantly with the hydrogen of the SH group.

The kinetics and mechanisms of the CH_3_SH + OH have been investigated extensively by experimental researchers^19–27^. The measured values for the rate constant at room temperature were in the range 2.1–9.04 in unit 10^–11^ cm^3^ molecule^−1^ s^−1^. The reported rate constants will be discussed in more detail later. Masgrau et al.^28^ have investigated theoretically the title reaction by MCCM-CCSD(T)-CO-2m/cc-pVDZ//MP2(full)/cc-pVDZ level. They have used VTST theory for the calculation of the rate constant and the obtained result at this level showed that the rate constants of H-abstraction from the SH and CH_3_ groups at 298 K were 8.85 × 10^–12^ and 2.95 × 10^–14^ cm^3^ molecule^−1^ s^−1^, respectively. In addition to the main pathway, some of the addition–elimination reactions of the CH_3_SH + OH were proposed computationally using the G1 method by Muino^29^.

In this article, we argue the connection between excitation energies and the stability of prereactive complexes as a starter of the reaction in the presence of sunlight. Using these results along with kinetic calculations, we answer this question “which reaction has the most probability to occur in the atmospheric condition?’’. It is shown that the methodology used here has precise results in the kinetic calculations of the title reaction in comparison with the previous study. Using the validated meta hybrid M06-2X method, the effect of pressure is cleared that has been less attended in the mentioned theoretical and the most experimental studies until now. We demonstrate that the pressure and altitude have significant effects on the rate of the CH_3_SH + OH reaction. Moreover, similar to our previous work^30^ on the reactions of methyl mercaptan, we pay attention (a) to complete investigation of the mechanism of CH_3_SH + OH reaction (b) to obtain more accurate theoretical intuition of the potential energy surface (c) to calculate pressure/temperature dependent rate constants with high accuracy, and (d) to get precise thermodynamic viewpoints on the title reaction. Therefore, the CH_3_SH + OH reaction pathways are studied using both wave function and density functional theories to obtain reliable potential energy surface, precise rate constants of main paths, and thermodynamic parameters of all components in the proposed PES. Therefore, the multi-well potential energy surface is calculated by higher-level calculations. The coupled-cluster method including triple excitations such as the UCCSD(T)/aug-cc-PV(T + d)Z//UMP2/aug-cc-pVTZ and the UCCSD(T)/6–311 +  + g (3df,3pd)//UMP26-311 +  + g (3df,3pd) levels are used to get an accurate and complete treatment of the mentioned reaction pathways. The stability of all adducts is examined energetically using thermodynamic parameters at the UCCSD(T)/aug-cc-PV(T + d)Z(energies) + UMP2/aug-cc-pVTZ (thermodynamic corrections) level at room temperature. In addition, to get the nature of interactions of pre-reactive collision complexes, topological analysis of the electronic charge density is implemented. High-pressure limit rate constants are carried out by using transition state (TST) and VTST theories for the reaction paths with one transition state (second-order elementary reactions), and Rice–Ramsperger–Kassel–Marcus (RRKM) theory is used for the low-pressure limit rate constant and its behavior in the fall of regime region.

## Methodology

In the reaction of CH_3_SH + OH, all stationary points on the ground-state of the multi-well PES were fully optimized using the UMP2 (wave function)^31^, UM06-2X^32,33^, and UB3LYP^34,35^ (DFT) along with the Dunning’s augmented correlation-consistent polarized valence triple zeta, aug-cc-pVTZ^36^, and split-valence augmented triple zeta, 6–311 +  + G(3df,3pd), basis sets. The harmonic vibrational frequencies were also computed for all stationary points at the mentioned level of theories. The calculated frequencies were used to provide the zero-point vibrational energy (ZPE), enthalpy, Gibbs free energy, and absolute entropy for each stationary point. Also, the vibrational frequencies were applied to confirm the nature of the corresponding stationary points as minimum structure or transition state structure in the PES. All thermodynamic parameters were computed by assuming each stationary point has an ideal gas behavior of the standard pressure of 1 atm temperature of 298.15 K. Moreover, the intrinsic reaction coordinate (IRC)^37,38^ was implemented to ensure that the transition states have connected to the desired reactants, complex reactants, intermediate and complex products. IRC calculations were done at the UMP2/6-311 +  + G(d,p) level of theory for all transition states. The energies, geometries, and the harmonic vibrational frequencies in different levels were obtained to examine the sensitivity of the stationary points to different methods and basis sets and to assess the reliability of the mentioned levels in the prediction of minimum structures and the transition state structures. Since, it is well-known that the single reference methods such as UMP2 and UM06-2X, UB3LYP without including some important excitations have some large error on the estimated energy (for example for H atom abstraction reactions the UB3LYP method underestimates the computed energy barriers). Therefore, we carried out (frozen core) single-point calculation using coupled-cluster (CC) theory. In CC calculation entire single and the double excitations accompanied by the perturbative treatment of the entire connected triple excitations were included in a single reference determinant of the UHF formalism,. The UCCSD(T)^39,40^ calculations were carried out on the geometries optimized at the UMP2/aug-cc-pVTZ level. In the UCCSD(T) calculations the high exponent *d* functions for the sulfur atom^41–44^ were included in combination with the aug-cc-pVTZ basis set to generate the aug-cc-pV(T + d)Z basis set. The topological analysis based on the theory of atoms in molecules (AIM)^45^ was employed to determine the bonding features of in complexes using the analysis of the electronic charge density and its Laplacian at critical points. For this purpose, the Hassian Matrix as a first-order density matrix was computed at the UMP2/aug-cc-pVTZ level of theory.

T1 diagnostic values play an important role in open-shell systems because of the need for multi-reference calculations if the threshold value is larger than 0.45. T1 diagnostic values of all stationary points were calculated. All T1 diagnostic values are below 0.037^46–49^.

All of the high-pressure limit rate constants were calculated using Gpop program^50^ based on the transition state (TST) and variational transition state (VTST) theories and the reaction rate in the low-pressure limit and the fall of the regime were computed using strong collision master equation/ RRKM theory by Ssumes program^51^. The Eckart tunneling correction^52^ was used for the inclusion of quantum effects in rate constant calculation. The Gaussian suite program^53^ was executed for the electronic structure calculation of the title reaction components.

When an unrestricted Hartree–Fock based method like the UMP2 method was applied to calculate the electronic structure of an open-shell system, the eigenvalues of the **S**^2^ operator are key parameters to assess spin contamination. The quantity of the spin contamination is gained by the expectation value of the **S**^2^ operator, <**S**^2^>. The expectation value of <**S**^2^> of the UHF base methods obtains from the spatial overlaps among all pairs of $\alpha$ and $\beta$ spin-orbitals as follows:

$$\left\langle {S^{2} } \right\rangle = S_{Z} (S_{Z} + 1) + N_{\beta } - \sum\limits_{ij}^{{N_{MO} }} {\left\langle {\phi_{i}^{\alpha } |\phi_{j}^{\beta } } \right\rangle }$$

If all $\alpha$ and $\beta$ orbitals are the same, there is no spin contamination in the considered system. If the amount of the expectation value <**S**^2^> is greater than 5% of the pure spin state, the used wave function is unacceptable for computing the energy^54^. Our results show that the spin is contaminated at the MP2/aug-cc-pVTZ level. Spin contamination makes the computed energies to be unacceptable at the mentioned level. Thus, we extract the energies at the PMP2/aug-cc-pVTZ level ( P refers to the projected wave function). All of the energies reported in the text are based on the UCCSD(T)/aug-cc-pV(T + d)Z level, unless we have mentioned the level of computation.

## Results and discussion

**Figure 1:**
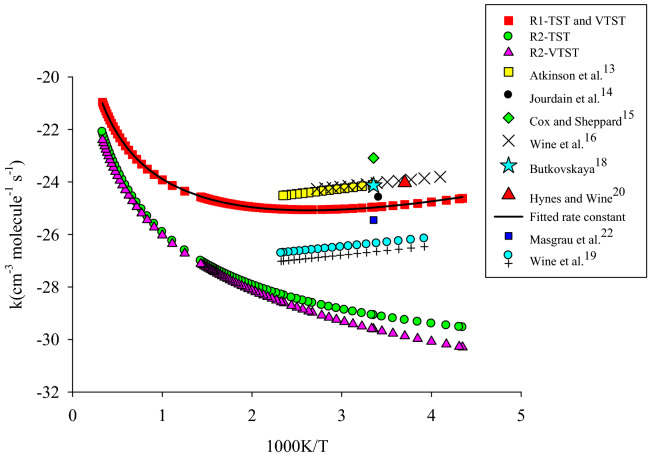
High pressure limit rate constants of the CH_3_SH + OH reaction. R1 and R2 refer to the R1 and R2 pathways. The rate constant computed by TST and VTST theories for R1 pathway is fitted in the non-Arrhenius temperature dependence rate constant.

All of the computed relative energies (in kcal/mol) at the different methods are improved by ZPE correction. The corrections on the absolute energies calculated at the MP2, M06-2X, and B3LYP methods are done by the zero-point energy that is computed at the same level. Also, the ZPEs at the MP2 level are implemented for the correction of the UCCSD(T) energies for kinetic calculations. The relative energies of different species at the mentioned levels are tabulated in Table 2. The potential energy surface for all reactions is plotted by relative energies at the UCCSD(T)/aug-cc-pV(T + d)Z level and depicted in Fig. 4. The geometrical parameters of the optimized structures such as the reactants, the primary complexes, the transition states, the post reactive complexes, and the products are represented in Fig. 3 at the UMP2/aug-cc-pVTZ level of theory. For all stationary points, the extracted T1 diagnostic values and the expectation values of the S^2^ operator are listed in Table S4 at the UCCSD(T)/aug-cc-pV(T + d)Z and the UMP2/aug-cc-pVTZ levels, respectively. All thermodynamic parameters are calculated at the UMP2/aug-cc-pVTZ level and collected in Table 3. Calculated equilibrium constants and rate constant values using the mentioned theories are collected in Table 1. The Arrhenius plot of the P1 and P2 products are depicted in Fig. 1. Throughout the paper, all thermodynamic values are reported at temperature 298.15 K and pressure 1 atm.

**Table 1:** Equilibrium constants (cm^3^ molecule^−1^) calculated at the UCCSD(T)/aug-cc-pV(T + d)Z level of initial complexes in the temperature range of 150–3000 K.

CR1				CR2				CR3			
T/K	K	T/K	K	T/K	K	T/K	K	T/K	K	T/K	K
150	3.11E − 19	580	1.84 E − 24	150	3.51 E − 18	580	9.67 E − 23	150	4.53 E − 22	580	3.82 E − 22
160	1.10 E − 19	590	1.74 E − 24	160	1.34 E − 18	590	9.26 E − 23	160	3.96 E − 22	590	3.90 E − 22
170	4.36 E − 20	600	1.65 E − 24	170	5.74 E − 19	600	8.88 E − 23	170	3.54 E − 22	600	3.99 E − 22
180	1.92 E − 20	610	1.56 E − 24	180	2.71 E − 19	610	8.54 E − 23	180	3.24 E − 22	610	4.08 E − 22
190	9.22 E − 21	620	1.48 E − 24	190	1.39 E − 19	620	8.23 E − 23	190	3.01 E − 22	620	4.16 E − 22
200	4.76 E − 21	630	1.41 E − 24	200	7.66 E − 20	630	7.94 E − 23	200	2.83 E − 22	630	4.26 E − 22
210	2.62 E − 21	640	1.35 E − 24	210	4.48 E − 20	640	7.67 E − 23	210	2.69 E − 22	640	4.35 E − 22
220	1.52 E − 21	650	1.29 E − 24	220	2.76 E − 20	650	7.43 E − 23	220	2.59 E − 22	650	4.44 E − 22
230	9.28 E − 22	660	1.23 E − 24	230	1.77 E − 20	660	7.20 E − 23	230	2.51 E − 22	660	4.54 E − 22
240	5.90 E − 22	670	1.18 E − 24	240	1.19 E − 20	670	6.99 E − 23	240	2.45 E − 22	670	4.64 E − 22
250	3.89 E − 22	680	1.14 E − 24	250	8.24 E − 21	680	6.80 E − 23	250	2.41 E − 22	680	4.73 E − 22
260	2.65 E − 22	690	1.09 E − 24	260	5.90 E − 21	690	6.62 E − 23	260	2.38 E − 22	690	4.84 E − 22
270	1.86 E − 22	700	1.05 E − 24	270	4.33 E − 21	700	6.45 E − 23	270	2.36 E − 22	700	4.94 E − 22
280	1.34 E − 22	710	1.02 E − 24	280	3.26 E − 21	710	6.30 E − 23	280	2.35 E − 22	710	5.04 E − 22
290	9.91 E − 23	720	9.84 E − 25	290	2.51 E − 21	720	6.16 E − 23	290	2.35 E − 22	720	5.15 E − 22
298	7.90 E − 23	730	9.52 E − 25	298	2.07 E − 21	730	6.02 E − 23	298	2.35 E − 22	730	5.26 E − 22
298.15	7.86 E − 23	740	9.23 E − 25	298.15	2.06 E − 21	740	5.90 E − 23	298.15	2.35 E − 22	740	5.37 E − 22
300	7.47 E − 23	750	8.96 E − 25	300	1.97 E − 21	750	5.78 E − 23	300	2.35 E − 22	750	5.48 E − 22
310	5.74 E − 23	760	8.70 E − 25	310	1.57 E − 21	760	5.67 E − 23	310	2.36 E − 22	760	5.59 E − 22
320	4.49 E − 23	770	8.46 E − 25	320	1.28 E − 21	770	5.57 E − 23	320	2.38 E − 22	770	5.71 E − 22
330	3.57 E − 23	780	8.24 E − 25	330	1.05 E − 21	780	5.47 E − 23	330	2.40 E − 22	780	5.82 E − 22
340	2.88 E − 23	790	8.03 E − 25	340	8.78 E − 22	790	5.38 E − 23	340	2.42 E − 22	790	5.94 E − 22
350	2.35 E − 23	800	7.84 E − 25	350	7.41 E − 22	800	5.30 E − 23	350	2.45 E − 22	800	6.06 E − 22
360	1.95 E − 23	900	6.42 E − 25	360	6.33 E − 22	900	4.70E − 23	360	2.48E − 22	900	7.36E − 22
370	1.63E − 23	1000	5.61E − 25	370	5.46E − 22	1000	4.39E − 23	370	2.52E − 22	1000	8.85E − 22
380	1.38E − 23	1100	5.13E − 25	380	4.75E − 22	1100	4.25E − 23	380	2.56E − 22	1100	1.05E − 21
390	1.17E − 23	1200	4.85E − 25	390	4.17E − 22	1200	4.21E − 23	390	2.60E − 22	1200	1.24E − 21
400	1.01E − 23	1300	4.69E − 25	400	3.69E − 22	1300	4.24E − 23	400	2.64E − 22	1300	1.44E − 21
410	8.78E − 24	1400	4.62E − 25	410	3.29E − 22	1400	4.33E − 23	410	2.69E − 22	1400	1.67E − 21
420	7.68E − 24	1500	4.61E − 25	420	2.95E − 22	1500	4.46E − 23	420	2.74E − 22	1500	1.92E − 21
430	6.77E − 24	1600	4.65E − 25	430	2.66E − 22	1600	4.62E − 23	430	2.79E − 22	1600	2.19E − 21
440	6.00E − 24	1700	4.73E − 25	440	2.42E − 22	1700	4.82E − 23	440	2.85E − 22	1700	2.48E − 21
450	5.36E − 24	1800	4.84E − 25	450	2.21E − 22	1800	5.04E − 23	450	2.90E − 22	1800	2.79E − 21
460	4.81E − 24	1900	4.97E − 25	460	2.03E − 22	1900	5.28E − 23	460	2.96E − 22	1900	3.13E − 21
470	4.34E − 24	2000	5.13E − 25	470	1.87E − 22	2000	5.55E − 23	470	3.02E − 22	2000	3.49E − 21
480	3.93E − 24	2100	5.30E − 25	480	1.73E − 22	2100	5.84E − 23	480	3.09E − 22	2100	3.88E − 21
490	3.58E − 24	2200	5.50E − 25	490	1.61E − 22	2200	6.14E − 23	490	3.15E − 22	2200	4.29E − 21
500	3.28E − 24	2300	5.71E − 25	500	1.50E − 22	2300	6.47E − 23	500	3.22E − 22	2300	4.73E − 21
510	3.02E − 24	2400	5.94E − 25	510	1.41E − 22	2400	6.82E − 23	510	3.29E − 22	2400	5.19E − 21
520	2.78E − 24	2500	6.18E − 25	520	1.32E − 22	2500	7.18E − 23	520	3.36E − 22	2500	5.68E − 21
530	2.58E − 24	2600	6.44E − 25	530	1.25E − 22	2600	7.56E − 23	530	3.43E − 22	2600	6.20E − 21
540	2.40E − 24	2700	6.71E − 25	540	1.18E − 22	2700	7.97E − 23	540	3.50E − 22	2700	6.74E − 21
550	2.23E − 24	2800	6.99E − 25	550	1.12E − 22	2800	8.39E − 23	550	3.58E − 22	2800	7.31E − 21
560	2.09E − 24	2900	7.29E − 25	560	1.06E − 22	2900	8.82E − 23	560	3.66E − 22	2900	7.91E − 21
570	1.96E − 24	3000	7.60E − 25	570	1.01E − 22	3000	9.28E − 23	570	3.74E − 22	3000	8.54E − 21

### Enthalpy and free energy formation, relative stability, and nature of interactions in the prereactive complexes

**Figure 2:**
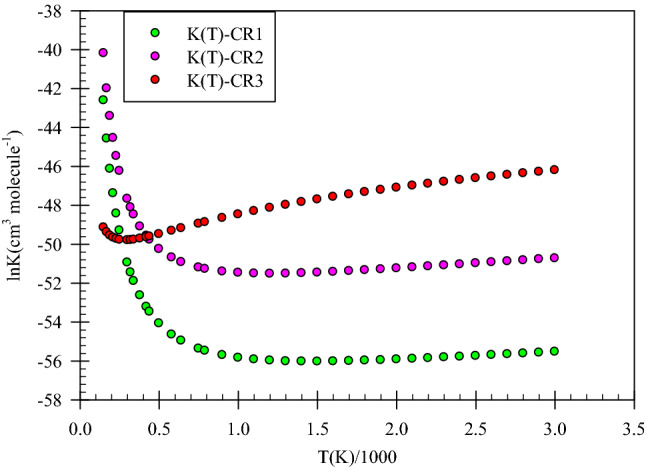
Equilibrium constants of the initial complexes computed at the UCCSD(T)/aug-cc-pV(T + d)Z level of theory.

The enthalpy formation of each pre-reactive complexes are calculated by the following equation

$$CH_{3} SH + OH\overset {K_{i} } \longleftrightarrow CR_{i} \quad \Delta X_{298.15}^{0} = X_{298.15}^{0} (CR_{i} ) - \left( {X_{298.15}^{0} (CH_{3} SH) + X_{298.15}^{0} (OH)} \right)$$

where K_i_ is the equilibrium constant of *i*-*th* complex (*i* = 1, 2 and 3 in respect of three pre-reactive complexes), ${X}_{298}^{^\circ }\left(\mathrm{S}\right)$ is the absolute enthalpy or free energy of S species. All equilibrium constants computed at the UCCSD(T)/aug-cc-pV(T + d)Z + UMP2/aug-cc-pVTZ level are sketched in Fig. 2 and tabulated in Table 1 and all of the reported $X_{298.15}^{0} (Y)$ values (*Y* is reaction components) in this study are based on the results of the UCCSD(T)/aug-cc-pV(T + d)Z + UMP2/aug-cc-pVTZ level (the total energies from UCCSD(T) method and thermodynamic corrections from the UMP2 method) and are collected in Table 3.

**Figure 3:**
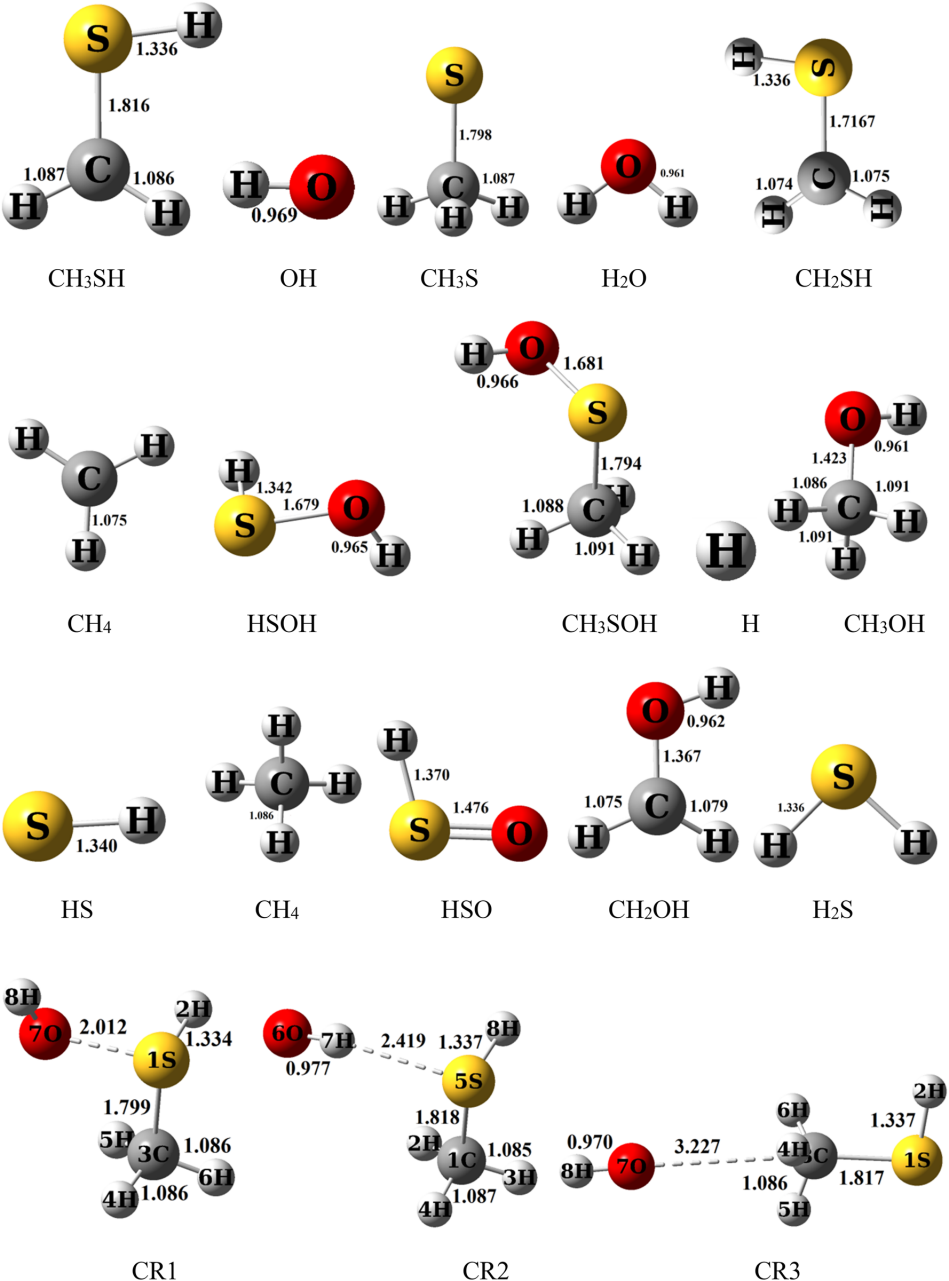

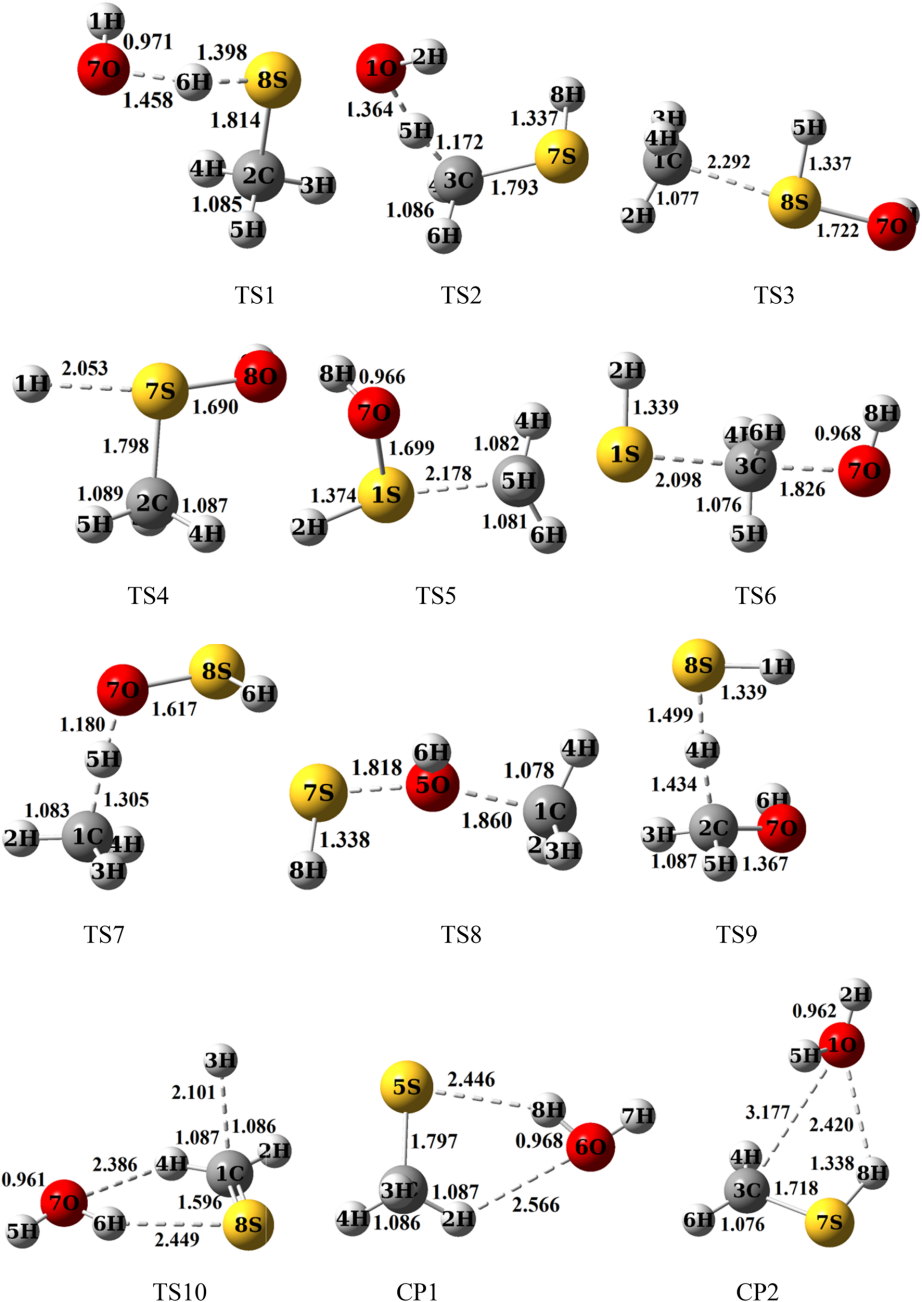

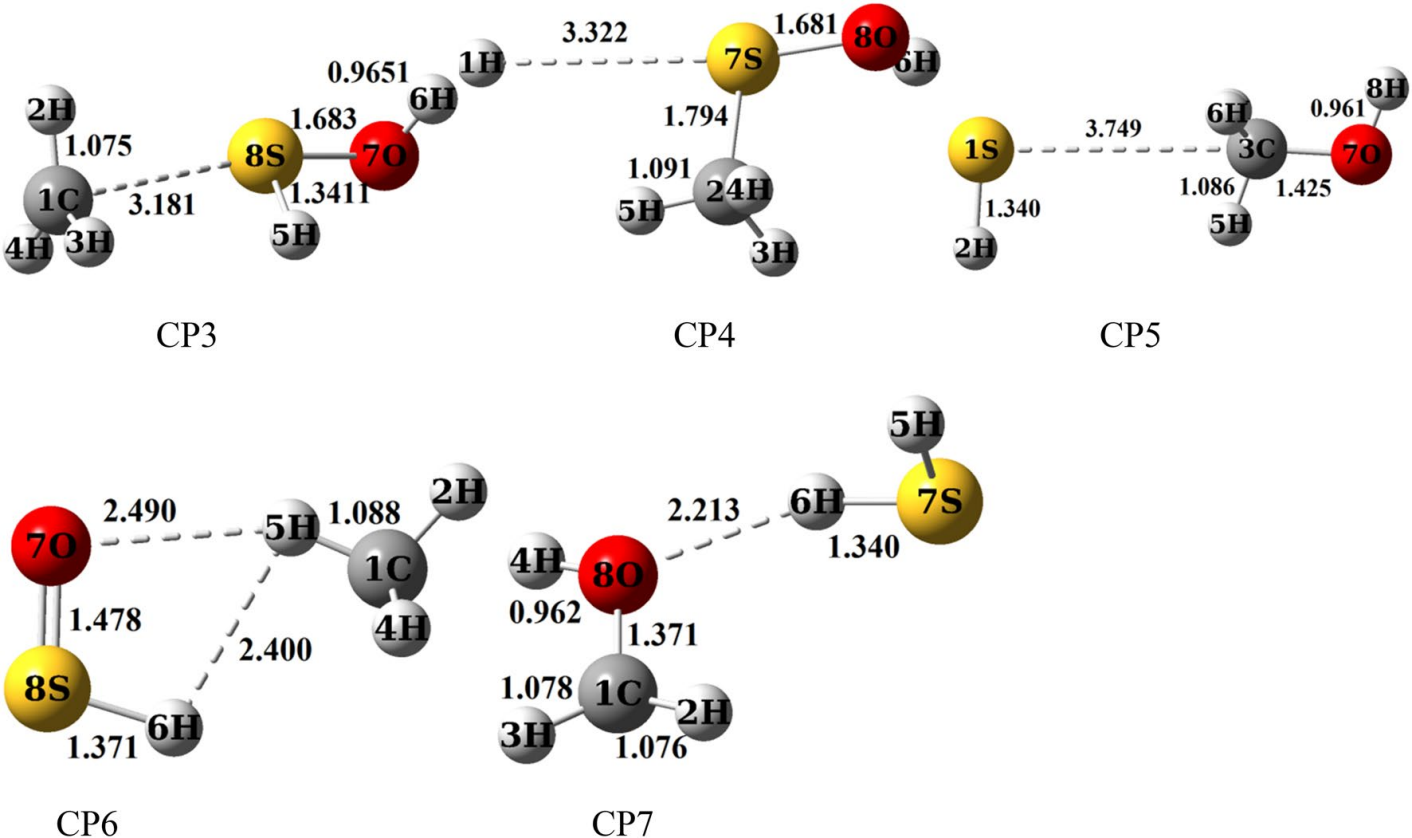
Bond lengths of all species (in angstrom) in the CH_3_SH + OH reaction calculated at the MP2/aug-cc-pVTZ level of theory.

**Figure 4:**
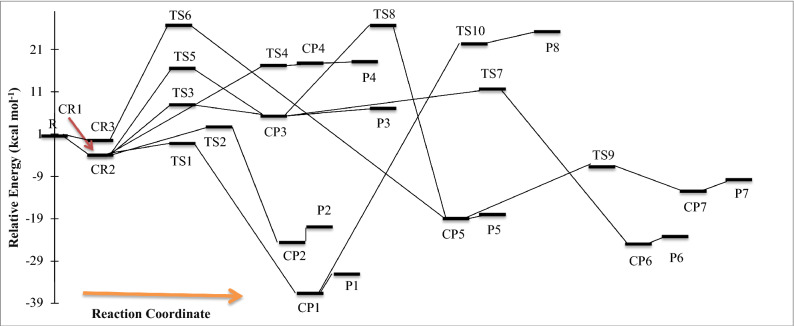
Ground state potential energy surface of the CH_3_SH + OH reaction sketched at the UCCSD(T)/aug-cc-pV(T + d)Z level.

In Fig. 3, It is shown that three collisions pre-reactive complexes CR1, CR2, and CR3 are formed resulting from the collision of two reactants. The CR1 complex has relative stability of 4.47 kcal/mol in comparison with the original reactants. This value is 1.16 kcal/mol lower than the energy of the corresponding structure in the Muino study. In this complex, the atom S interacts with the atom O, and the bond distance S…O is 2.012 Å. The formation of CR1 complexes is exothermic and nonspontaneous with standard enthalpy and Gibbs free energy changes of − 2.27 and 6.97 kcal/mol, respectively. The CR2 complex is more stable than the CR3 complex. In the structure of the CR2 complex, the atom 7H of the radical hydroxyl is close to the sulfur atom of the methyl sulfide. The distance 7H–5S is 2.421 Å. The CR2 complex is formed without any barrier and has − 4.48 kcal/mol relative energy. The computed thermodynamic parameters, ∆*H*^0^ and ∆*G*^0^, for the CR2 complex are − 3.36 and 3.26 kcal/mol, respectively, that show the formation of CR2 is also an exothermic and nonspontaneous process. In the third initial complex, CR3, the atom O of hydroxyl radical interacts with the atom C of methyl sulfide, in which the distance O…C is 3.22 Å and has the relative energy of − 1.05 kcal/mol (see Table 2). Also, the formation of the CR3 is an exothermic and nonspontaneous process with ∆*H*^0^ = − 0.17 and ∆*G*^0^ = 3.52 kcal/mol. On the bases of the sketched PES, the reaction of methyl sulfide with hydroxyl radical is started with the three pre-reactive complexes on the doublet potential energy surface, and finally, 13 different products are predicted. The PES profile (relative energy versus reaction coordinate) is plotted in Fig. 4 at the ground state of all species based on the calculated energies at the UCCSD(T) level. The details of the reaction pathways are described in the next section.

**Table 2:** Relative energies of all species (in kcal/mol) in the CH_3_SH + OH reaction calculated on different computational levels.

Species	MP2 + ZPE		PMP2		M062X + ZPE		CCSD(T)		B3LYP + ZPE
	aug-cc-pVTZ	6–311 + + g(3df,3pd)	aug-cc-pVTZ	6–311 + + g(3df,3pd)	aug-cc-pVTZ	6–311 + + g(3df,3pd)	aug-cc-pV(T + d )Z	6–311 + + g(3df,3pd)	6–311 + + g(3df,3pd)
R	0.00	0.00	0.00	0.00	0.00	0.00	0.00	0.00	0.00
CR1	− 2.37	− 2.33	− 7.86	− 3.42	− 4.71	− 4.84	− 4.47	− 3.39	− 8.47
CR2	− 3.24	− 3.22	− 4.67	− 3.71	− 2.78	− 3.47	− 4.48	0.97	− 2.46
CR3	− 0.60	− 0.57	− 0.99	− 0.85	− 0.45	− 0.54	− 1.05	− 1.09	− 0.15
TS1	1.09	1.32	− 0.44	3.22	− 1.26	− 1.23	− 1.74	− 1.55	− 5.44
TS2	3.74	3.48	3.42	3.94	1.10	0.99	2.15	1.88	− 1.19
TS3	8.22	8.47	4.23	7.87	8.23	7.83	7.37	8.79	5.51
TS4	11.66	12.18	11.68	15.25	14.96	14.49	16.72	17.68	−
TS5	16.71	16.90	13.01	16.09	16.97	16.01	16.00	17.50	−
TS6	33.30	34.69	24.20	34.18	26.95	27.46	26.14	50.54	21.74
TS7	10.98	10.78	9.64	18.82	−	−	11.08	12.12	3.17
TS8	29.25	30.51	21.64	31.17	24.79	25.50	26.12	27.62	19.11
TS9	− 10.58	− 11.25	− 11.78	− 8.10	−	− 10.81	− 7.31	− 8.34	− 12.95
TS10	19.48	18.60	15.67	23.00	19.34	19.12	21.74	20.93	19.27
CP1	− 38.71	− 38.92	− 42.14	− 3.42	− 35.72	− 35.77	− 37.22	− 37.84	− 33.75
CP2	− 27.85	− 29.13	− 28.93	− 29.97	− 24.12	− 24.72	− 25.12	− 26.06	− 24.96
CP3	1.79	1.90	2.58	7.26	2.71	−	4.69	5.46	3.16
CP4	8.56	9.05	11.30	308.30	−	14.38	17.20	18.45	17.87
CP5	− 19.50	− 21.15	− 22.85	− 22.75	− 18.62	− 18.50	− 19.54	− 20.28	−
CP6	− 22.04	− 24.42	− 25.72	− 28.32	− 22.65	− 24.50	− 25.51	− 24.29	− 28.38
CP7	− 16.17	− 17.26	− 15.84	− 17.91	− 13.53	− 14.08	− 13.04	− 14.21	− 15.65
P1(CH_3_S + H_2_O)	− 35.77	− 35.87	− 37.39	− 36.30	− 31.19	− 30.76	− 32.58	− 33.08	− 31.88
P2(CH_2_SH + H_2_O)	− 25.84	− 27.28	− 25.24	− 25.09	− 22.26	− 22.61	− 21.43	− 22.28	− 24.36
P3(CH_3_ + HSOH)	2.91	3.04	4.41	5.98	4.49	4.41	6.50	7.34	3.28
P4(CH_3_OSH + H)	8.54	9.02	11.56	11.98	13.97	13.55	17.57	18.76	28.31
P5(CH_3_OH + SH)	− 20.02	− 20.56	− 21.90	− 21.76	− 18.06	− 17.87	− 18.58	− 19.60	− 18.37
P6(CH_4_ + HSO)	− 21.04	− 23.44	− 23.81	− 22.94	− 22.01	− 23.75	− 23.75	− 22.53	− 28.59
P7(CH_2_OH + H_2_S)	− 14.23	− 15.33	− 11.67	− 13.13	− 12.74	− 13.16	− 10.32	− 11.46	− 14.94
P8(CH_2_S + H + H_2_O)	11.43	10.57	18.38	17.40	20.54	20.37	24.69	23.58	20.77

The equilibrium constants of the CR1, CR2, and CR3 complexes are calculated in the range of 230–3000 K. The equilibrium constant of the CR1 is decreased until 900 K, and it remains roughly constant after this temperature. Also, the same behavior was observed for the CR2. At the mentioned temperature range, the equilibrium constant of the CR3 is increased. The computed equilibrium constants for the CR1, CR2, and CR3 complexes at room temperature are 7.86 × 10^–23^, 2.06 × 10^–21^, and 2.35 × 10^–22^ cm^−3^ molecule^−1^, respectively. On the basis of the calculated equilibrium constants at the UCSD(T)/aug-cc-pV(T + d)Z level, we conclude that the CR2 and CR1 are more stable than the CR3 in low temperatures, but at the moderate and high temperatures, the CR3 equilibrium constant is more than the others.

The topological analysis (AIM calculation) of the electron charge density in the mentioned complexes reveals the existence of bond critical points among the abovementioned interactions. In the CR1complex, the bond critical point between the S…O bond shows a noncovalent interaction with the charge density of *ρ* = − 0.1080 e bohr^−3^ and the charge density Laplacian of ∇^2^*ρ* = 0.1259 e bohr^−5^. The bond critical point in CR2 located between the atoms 7H and S (*ρ* = 0.01847 e bohr^−3^ and ∇^2^*ρ* = 0.0437 e bohr^−5^) indicates a van der Waals interaction. The interaction between atoms O and C of the third complex is also a van der Waals (*ρ* = 0.0040 e bohr^−3^ and ∇^2^*ρ* = 0.0204 e bohr^−5^), but it is weaker than the interaction of the CR2 complex.

### Pathways description

#### P1 (H_2_O + CH_3_S) and P2 (H_2_O + CH_2_SH) production pathways

The R1 and R2 pathways are H abstraction paths from SH and CH_3_ groups of CH_3_SH to generate the P1 and P2 products, respectively. The possible paths are as follows:

R1 $$R\left( {CH_{3} SH + OH} \right) \to CR1 \to TS1 \to CP1 \to P1$$

R2 $$R \to CR2 \to TS2 \to CP2 \to P2$$

The CR1 complex is transformed via TS1 to the CP1 product complex by passing through the barrier-less process with the relative energy − 1.74 kcal/mol. Also, this path was considered by Muino. The computed relative energy for a similar structure was − 2.029 kcal/mol at the G1 level. IRC calculation shows that the atom 8H is shifting from the atom S of CH_3_SH to the atom O of OH fragment. In the structure of the TS1, the H–S bond is breaking up between the atoms 8H and 5S with a length of 1.398 Å and, the O–H bond is forming between the atoms 6O and 8H with a length of 1.454 Å. The imaginary frequency of this saddle point in the mentioned conversion is 1051 cm^−1^ at the MP2/aug-cc-pVTZ level of theory. The CP1 complex without passing any transition state converts to the P1 as a final product. The reverse reaction has less important due to the need for a high energy barrier (35.48 kcal/mol). It may relate to the low electronegativity of the sulfur atom in comparison with the atom O. So, the bond between the atoms O and H (123.58 kcal/mol) in isolated water is stronger than the bond between the atoms S (91.00 kcal/mol) and H in methanethiol.

Regarding the above-mentioned path, the CR2 complex via TS2 by surmounting on the barrier of 6.63 kcal/mol converts to the CP2 product complex. The transition state TS2 is 2.15 kcal/mol higher than CH_3_SH + OH on the proposed PES. Then, CP2 yields the P2 product by breaking up the bonds between CH_2_SH + H_2_O moieties. The transition state 2 involves cleavages the bond between the atoms 3C and 5H with a length of 1.725 Å and also at the same time, it includes the formation bond between the atoms 1O and 5H with a length of 1.364 Å. The reverse reaction of this path same as the previous path requires high energy to occur (27.27 kcal/mol). Here the reason is different than the first path. As we know, the electronegativity of the carbon atom is lower than the oxygen atom, and also the stability of methyl radical is lower than OH radical. Because the high electronegativity of OH radical cause the radical electron roughly stable in comparison with that electron when it locates on the carbon atom.

The AIM calculations show that the optimized structure of the CP2 has a four-member ring structure with ring critical parameters ($\rho_{rcp}$ = 0. 0064 e bohr^−3^ and $\nabla^{2} \rho_{rcp}$ = 0.0312 e bohr^−5^). It contains a van der Waals interaction between the atoms O and 8H ($\rho$ = 0.0097 and $\nabla^{2} \rho$ = 0.0412) and between the atoms O and 3C ($\rho$ = 0.0071 and $\nabla^{2} \rho$ = 0.0313). Also, the same interaction is observed between the atoms H and C ($\rho$ = 0.0071 e bohr^−3^ and $\nabla^{2} \rho$ = 0.0076 e bohr^−5^).

On the basis of the collected data in Table 3, we can conclude that the P1 and P2 formation processes are thermodynamically favorable with the standard enthalpy of − 31.12 and − 22.09 kcal/mol and Gibbs free energy of − 32.03 and − 23.90 kcal/mol. This is an exothermic and spontaneous process. Our computed enthalpy is comparable with that values found by Muino ($\Delta H^{0}$ = − 33.90 kcal/mol), but the Gibbs free energies have a large difference ($\Delta G^{0}$ = − 34.01 kcal/mol).

**Table 3:** Thermodynamic parameters of all species (in kcal/mol) in the CH_3_SH reaction computed at the UCCSD(T)/aug-cc-pV(T + d)Z (total energies) + UMP2/aug-cc-pVTZ (thermodynamic corrections) level.

Species	*∆Eº*	*∆Hº*	*∆Gº*	*T∆Sº*
R	0.00	0.00	0.00	0.00
CR1	− 1.67	− 2.27	6.97	− 9.23
CR2	− 2.76	− 3.36	3.26	− 6.62
CR3	0.42	− 0.17	3.52	− 3.69
TS1	− 1.85	− 2.45	5.63	− 8.07
TS2	− 3.39	− 3.99	4.51	− 8.50
TS3	8.30	7.71	16.71	− 9.00
TS4	15.43	14.84	24.21	− 9.37
TS5	16.80	16.21	25.58	− 9.37
TS6	27.48	26.89	35.14	− 8.25
TS7	8.96	8.36	16.23	− 7.87
TS8	27.07	26.48	34.91	− 8.43
TS9	− 8.48	− 9.07	− 0.66	− 8.41
TS10	19.69	19.09	25.15	− 6.05
CP1	− 33.44	− 34.04	− 27.85	− 6.18
CP2	− 23.64	− 24.24	− 18.70	− 5.54
CP3	5.23	4.64	8.49	− 3.85
CP4	16.28	15.69	21.74	− 6.06
CP5	− 15.42	− 16.01	− 12.37	− 3.64
CP6	− 23.00	− 23.60	− 18.34	− 5.25
CP7	− 12.37	− 12.96	− 8.30	− 4.66
P1(CH_3_S + H_2_O)	− 31.12	− 31.12	− 32.03	0.92
P2(CH_2_SH + H_2_O)	− 22.09	− 22.09	− 23.90	1.81
P3(CH_3_ + HSOH)	5.22	5.22	3.64	1.58
P4(CH_3_OSH + H)	15.74	15.74	18.30	− 2.56
P5(CH_3_OH + SH)	− 16.98	− 16.98	− 16.93	− 0.05
P6(CH_4_ + HSO)	− 23.09	− 23.09	− 24.24	1.15
P7(CH_2_OH + H_2_S)	− 11.50	− 11.50	− 12.91	1.41
P8(CH_2_S + H + H_2_O)	19.76	20.35	12.21	8.14

#### P3 (CH_3_ + HSOH), P4 (H + CH_3_SOH) and P5 (SH + CH_3_OH) production pathways

The following schemes show the production pathways of P3, P4, and P5 adducts:

R3a $$R \to CR1 \to TS3 \to CP3 \to P3$$

R3b $$R \to CR1 \to TS5 \to CP3 \to P3$$

R4 $$R \to CR1 \to TS4 \to CP4 \to P4$$

R5a $$R \to CR3 \to TS6 \to CP5 \to P5$$

R5b $$R \to CR1 \to TS3 \to CP3 \to TS8 \to CP5 \to P5$$

R5c $$R \to CR1 \to TS5 \to CP3 \to TS8 \to CP5 \to P5$$

P3 adduct is produced by two paths. The R3a and R3b paths occur through only one transition state. The barrier energy of path R3a and path R3b are 11.84 and 20.46 kcal/mol, respectively. The CR1complex after passing the transition states TS3 and TS5 transforms into the CP5 complex. The mentioned transition states include methyl elimination from CH_3_SH and OH addition to the SH group. Firstly, the OH group contacts to the S atom. Secondly, the methyl group separates from CH_3_SH. The differences between the mentioned saddle points are related to the orientation of the methyl group and the distance of the atoms C and S. In the saddle point TS3, the angle C–S–O, and the distance C–O are 163.25° and 2.92 Å, but the corresponding angle in TS5 is 83.25° and 2.178 Å.

The CR1 is transformed into the CP4 by passing through TS4 with an energy height of 21.18 kcal/mol at the UCCSD(T) method. Then, without entrancing any barrier energy, the CP4product complex converts to the P4 as a final product. This path is the same as the P3 production paths, but the hydrogen atom is separated in this reaction. In other words, firstly, OH radical is closed to the atom S and crates a covalent bond and after that, the hydrogen atom of the SH functional group is separated. The final result of the R4 path is the replacement of the OH fragment with the H atom. This path is also investigated by Muino. He calculated relative energy for the same saddle point is 15.42 kcal/mol, which our result is 5.76 kcal/mol higher than that value.

From a geometrical point of view, the structure of TS4 involves the bond formation among the O and S atoms with a length of 1.690 Å and breaking up the bond between the atoms 1H and S atoms with a length of 2.053 Å. The imaginary frequency of TS4 at the UMP2 method is 668 cm^−1^ in the reaction coordinate. According to Fig. 4, between the separation of a methyl group and a hydrogen atom through P3–P5 paths, our results show that methyl elimination is energetically favorable than hydrogen elimination. These observations are related to the stability of the separated part. It is well known that the hydrogen atom is very unstable than many active atmospheric species. So, the CP4 product complex and also the P4 product are energetically unstable than other minimum structures. Therefore, it may back to reactants. The product complex CP3 and P3 product are also unstable than the original reactants, the reaction continuation from CP3 is favorable than the reverse reaction.

The product complex CP5 obtains via TS6 with the energy barrier of 27.19 kcal/mol relative to the CR3 complex. In this pathway, S_N_2 reaction has occurred, in which the OH functional group comes from one side and the SH functional group goes out from the other side. In the structure of TS6, the bond O–C with a length of 1.826 Å is weakening and the bond S–C with a length of 2.10 Å is forming. This reaction has a large energy barrier among the other discussed first step bimolecular reactions. So, in the kinetic point of view, it is slower than the other, but thermodynamically it is favorable than the products of the paths R3a–R4. Therefore the back reaction is unfavorable kinetically. The reason may relate to the stability of radical electron in the SH group, corresponding to the electron cloud distribution. Other paths for the generation of CP5 are the paths R5b and R5c. Until CP3 formation is discussed above. This complex by surmounting on the energy barrier of 21.42 kcal/mol through TS8 converts to CP5. Eventually, in the last step of the R3a–R5c paths, the P3, P4, and P5 products are generated without passing through an energy barrier from the corresponding product complexes.

The production processes of P3 and P4 in standard conditions are endothermic according to the computed enthalpies 5.22 and 3.84 kcal/mol and nonspontaneous concerning to the calculated free energies 15.74 and 18.30 kcal/mol, respectively. The large unstability of P4 may relate to the production of atomic hydrogen. But, the production process of P5 is exothermic with $\Delta H^{0}$ = − 6.98 kcal/mol and spontaneous with $\Delta G^{0}$ = − 16.93 kcal/mol.

#### P6 (CH_4_ + OSH), P7 (SH_2_ + CH_2_OH) and P8 (H_2_O + H + CH_2_S) production pathways

The following paths are designated to create the products P6, P7, and P8

R6a $$R \to CR1 \to TS3 \to CP3 \to TS7 \to CP6 \to P6$$

R6b $$R \to CR1 \to TS5 \to CP3 \to TS7 \to CP6 \to P6$$

R7a $$R \to CR1 \to TS3 \to CP3 \to TS8 \to CP5 \to TS9 \to CP7 \to P7$$

R7b $$R \to CR1 \to TS5 \to CP3 \to TS8 \to CP5 \to TS9 \to CP7 \to P7$$

R7c $$R \to CR3 \to TS6 \to CP5 \to TS9 \to CP7 \to P7$$

R8 $$R \to CR1 \to TS1 \to CP1 \to TS10 \to P8$$

The R6a and R6b paths are two-step reactions and so are occurring among two transition states (TS3 and TS7 or TS5 and TS7). Until CP3 generation was discussed above. The low-level transition state, TS7, with 6.39 kcal/mol height is a barrier between the CP5 and CP7. Also, the IRC calculation shows the CP6 complex product is created from the related transition state TS7. Abstracted hydrogen in the structure of the saddle point 7 has a weak bond with O atom with a length of 1.80 Å and a bond with C atom with a length of 1.305 Å.

There are two paths with three steps for the production of P7 (R7a and R7b) as shown above. The reaction mechanism until CP5 generation is investigated in the R5 pathway. The reaction is continued by surmounting on TS9 with an energy barrier of 12.23 kcal/mol. The IRC calculation shows that TS9 leads to the formation of CP7. From a geometrical point of view, to continue the reaction by TS9, the HS fragment in the CP5 is closed to the CH_3_OH fragment and gets a hydrogen atom from the methyl group of methanol. AIM calculations indicate the presence of a bond critical point between the atoms C and 4H ($\rho$ = 0.1196 e bohr^−3^ and $\nabla^{2} \rho$ = − 0.1558 e bohr^−5^) and the atoms S and 4H ($\rho$ = 0.1541 e bohr^−3^ and $\nabla^{2} \rho$ = − 0.3084 e bohr^−5^) in the TS9 structure, which shows the C–H covalent bond is weaker than that of in the CP5 ($\rho$ = 0.3028 e bohr^−3^ and $\nabla^{2} \rho$ = − 1.288 e bohr^−5^) and arising a weak covalent bond between the atoms S and 4H.

In the exit channels of the R6a–R7b paths, the CP6 and CP7 product complexes are turned to relevant the P6 and P7 products without passing any energy barrier. The relative energies of these complexes are − 25.51 and − 13.04 kcal/mol, respectively.

The final path (R8) leads to produce the H_2_O + H + CH_2_S species. This path is a bimolecular reaction with two steps. The first step is CP1 formation by path R1, and the second step is the generation of P8 by path R8. The CP1 complex may keep on the reaction by surmounting on TS10 and directly without entrancing complex product converts to P8. The barrier energy of TS10 is 58.96 kcal/mol.

According to the information of Table 3, the P6 and P7 formation processes with the standard enthalpies of − 23.09 and − 11.50 kcal/mol and Gibbs free energies of − 24.24 and − 12.91 kcal/mol are exothermic and spontaneous. These parameters for P8 are $\Delta H^{0}$ = 20.35 kcal/mol and $\Delta G^{0}$ = 12.21 kcal/mol which indicate an endothermic and nonspontaneous process.

### Rate constant calculation

#### High-pressure limit rate constant

For all possible elementary bimolecular reactions in the CH_3_SH + OH reaction, the temperature dependence of rate constants is computed by using both transition state theory (TST) and variational transition state theory (VTST) in conjunction with Eckert tunneling factor for rate constants improving. The suitable meta hybrid DFT method, UM06-2X/aug-cc-pVTZ, was performed for the extraction of experimental data from a theoretical investigation. The computed high-pressure limit rate constants at the mentioned levels in the temperature range of 150–3000 K are listed in Table 4. First of all, we study the kinetics of one step elementary reactions, which have only one transition state. Thus, in this part, the rates of CH_3_S + H_2_O and CH_2_SH + H_2_O pathways are computed. The rate constants of the products P1 (CH_3_S + H_2_O) through the R1 pathway by VTST theory at the UM06-2X/aug-cc-pVTZ level at temperatures of 150, 298.15, and 450 K are 6.74 × 10^–11^, 1.44 × 10^–11^, and 1.37 × 10^–11^ cm^−3^ molecule^−1^ s^−1^, respectively. The same values also are observed by TST theory (See Table S5) at this level. The rate constant of this path is also computed by TST theory at the UCCSD(T)/aug-cc-pV(T + d)Z (energies) + UMP2/aug-cc-pVTZ (partition functions and ZPEs) level. In temperatures of 150, 298.15, and 450 K, the calculated rate constants are 2.76 × 10^–10^, 2.09 × 10^–11^, and 1.18 × 10^–11^ cm^−3^ molecule^−1^ s^−1^, respectively. For the P2 product, The mentioned rate constants through the R2path and by using VTST theory at the UM06-2X/aug-cc-pVTZ level and the same temperatures are 5.36 × 10^–15^, 8.92 × 10^–14^, and 3.62 × 10^–13^ cm^−3^ molecule^−1^ s^−1^ and by using TST theory are 8.34 × 10^–14^, 2.36 × 10^–13^, and 5.77 × 10^–^13 cm^−3^ molecule^−1^ s^−1^, respectively. Our calculated rate expression k(T) = 1.893 × 10^–13^ (300/T)^−3.145^ exp[(1295.000 ± 6.300)/T] cm^3^ molecule^−1^ s^−1^ at 150–430 K temperature range by VTST and TST theories has a good agreement with the experimental rate constant measured by Atkinson et al.^13^ at 299–426 K (k(T) = 8.89 × 10^–12^ exp(790 ± 300)/RT), and Tyndall and Ravishankara^21^ in the 244–430 K range with the value of (3.3 ± 0.40) × 10^–11^ cm^3^ molecule^−1^ s^−1^. As mentioned above, the rate expression measured by the Atkinson group implies negative activation energy. This result is also observed in our computed rate constant for the R1 pathway (barrier less reaction). Also, our computed rate constant, 3.6 × 10^–11^ cm^3^ molecule^−1^ s^−1^, have excellent agreement with Jourdain et al. reported data. Their reported rate constant using the discharge flow—EPR—mass spectrometry method at temperatures of 293 K is (2.1 ± 0.2) × 10^–11^ cm^3^ molecule^−1^ s^−1^^14^. The experimental value of the title reaction rate at 298.15 K is measured with the value of k(298 K) = (9.04 ± 0.85) × 10^–11^ cm^3^ molecule^−1^ s^−1^ by Cox and Sheppard^15^. It is 6.71 times larger than the predicted value by the VTST rate (1.44 × 10^–11^ cm^3^ molecule^−1^ s^−1^). Also, good agreement is observed between the kinetic data of VTST theory, and Wine et al. have a reported rate expression, k(T) = (1.15 ± 0.39) × 10^–11^ exp[(338 ± 100)/T] cm^3^ molecule^−1^ s^−1^, over the temperature range 244–367 K. Also, they study the rate of reaction at 298 K and report the value of k = (3.37 ± 0.41) × 10^–11^ cm^3^ molecule^−1^ s^−1^^16^. On the other hand, Lee and Tang, Butkovskaya, and Wine et al. have measured the rate of CH_3_SH + OH reaction at room temperature at with the values of k = (2.56 ± 0.44) × 10^–11^, k = (3.3 ± 0.4) × 10^–11^, k = (3.3 ± 0.4) × 10^–11^, and k = (3.2–4.) × 10^–11^ cm^3^ molecule^−1^ s^−1^, respectively^17–19^, that have very excellent agreement with the computed value of rate in this temperature.

**Table 4:** High pressure limit rate constants (cm^3^ molecule^−1^ s^−1^) calculated by VTST theory at the UM06-2X/aug-cc-pVTZ level for the P1 and P2 adducts through the R1 and R2 pathways.

R1				R2			
T (K)	k	T(K)	k	T(K)	k	T(K)	k
150	6.74E − 11	580	1.67E − 11	150	5.36E − 15	580	8.37E − 13
160	5.29E − 11	590	1.71E − 11	160	7.33E − 15	590	8.85E − 13
170	4.30E − 11	600	1.74E − 11	170	9.71E − 15	600	9.35E − 13
180	3.60E − 11	610	1.77E − 11	180	1.25E − 14	610	9.88E − 13
190	3.09E − 11	620	1.81E − 11	190	1.58E − 14	620	1.04E − 12
200	2.71E − 11	630	1.85E − 11	200	1.96E − 14	630	1.10E − 12
210	2.42E − 11	640	1.88E − 11	210	2.39E − 14	640	1.15E − 12
220	2.19E − 11	650	1.92E − 11	220	2.88E − 14	650	1.21E − 12
230	2.02E − 11	660	1.96E − 11	230	3.43E − 14	660	1.27E − 12
240	1.87E − 11	670	2.01E − 11	240	4.04E − 14	670	1.33E − 12
250	1.76E − 11	680	2.05E − 11	250	4.71E − 14	680	1.39E − 12
260	1.67E − 11	690	2.09E − 11	260	5.44E − 14	690	1.46E − 12
270	1.59E − 11	700	2.14E − 11	270	6.25E − 14	700	1.53E − 12
280	1.53E − 11	710	2.18E − 11	280	7.13E − 14	710	1.60E − 12
290	1.48E − 11	720	2.23E − 11	290	8.09E − 14	720	1.67E − 12
298	1.44E − 11	730	2.28E − 11	298	8.91E − 14	730	1.75E − 12
298.15	1.44E − 11	740	2.33E − 11	298.15	8.92E − 14	740	1.82E − 12
300	1.44E − 11	750	2.38E − 11	300	9.12E − 14	750	1.90E − 12
310	1.40E − 11	760	2.43E − 11	310	1.02E − 13	760	1.98E − 12
320	1.38E − 11	770	2.49E − 11	320	1.14E − 13	770	2.07E − 12
330	1.35E − 11	780	2.54E − 11	330	1.27E − 13	780	2.15E − 12
340	1.34E − 11	790	2.60E − 11	340	1.41E − 13	790	2.24E − 12
350	1.33E − 11	800	2.65E − 11	350	1.56E − 13	800	2.33E − 12
360	1.32E − 11	900	3.30E − 11	360	1.71E − 13	900	3.40E − 12
370	1.31E − 11	1000	4.09E − 11	370	1.88E − 13	1000	4.78E − 12
380	1.31E − 11	1100	5.03E − 11	380	2.06E − 13	1100	6.51E − 12
390	1.31E − 11	1200	6.14E − 11	390	2.25E − 13	1200	8.64E − 12
400	1.32E − 11	1300	7.45E − 11	400	2.45E − 13	1300	1.12E − 11
410	1.32E − 11	1400	8.96E − 11	410	2.66E − 13	1400	1.43E − 11
420	1.33E − 11	1500	1.07E − 10	420	2.88E − 13	1500	1.80E − 11
430	1.34E − 11	1600	1.27E − 10	430	3.11E − 13	1600	2.23E − 11
440	1.35E − 11	1700	1.49E − 10	440	3.36E − 13	1700	2.73E − 11
450	1.37E − 11	1800	1.74E − 10	450	3.62E − 13	1800	3.31E − 11
460	1.38E − 11	1900	2.03E − 10	460	3.90E − 13	1900	3.97E − 11
470	1.40E − 11	2000	2.34E − 10	470	4.18E − 13	2000	4.71E − 11
480	1.42E − 11	2100	2.70E − 10	480	4.49E − 13	2100	5.55E − 11
490	1.44E − 11	2200	3.08E − 10	490	4.80E − 13	2200	6.50E − 11
500	1.46E − 11	2300	3.51E − 10	500	5.13E − 13	2300	7.55E − 11
510	1.48E − 11	2400	3.98E − 10	510	5.48E − 13	2400	8.72E − 11
520	1.50E − 11	2500	4.50E − 10	520	5.84E − 13	2500	1.00E − 10
530	1.53E − 11	2600	5.06E − 10	530	6.22E − 13	2600	1.14E − 10
540	1.56E − 11	2700	5.67E − 10	540	6.61E − 13	2700	1.30E − 10
550	1.58E − 11	2800	6.33E − 10	550	7.03E − 13	2800	1.47E − 10
560	1.61E − 11	2900	7.05E − 10	560	7.45E − 13	2900	1.66E − 10
570	1.64E − 11	3000	7.82E − 10	570	7.90E − 13	3000	1.86E − 10

Using electron pulsed laser photolysis-pulsed laser-induced fluorescence technique, Hynes and Wine have investigated the kinetics of OH reaction with CH_3_SH at the temperature of 300 K and a pressure of 700 Torr. They measured value is (3.27 ± 0.36) × 10^–11^ cm^3^ molecule^−1^ s^−1^ which has good agreement with the calculated rate constant at the mentioned temperature with the value of (1.44 ± 0.36) × 10^−11^ cm^3^ molecule^−1^ s^−1^^20^.

Masgrau et al. have calculated theoretically the rate constants of the R1 and R2 paths at the MCCM-CCSD(T) level^22^. The obtained results at this level show the rate constants of mentioned paths are 8.85 × 10^–12^ and 2.95 × 10^–14^ cm^3^ molecule^−1^ s^−1^ at 298 K and are 6.965 × 10^–12^ and 1.75 × 10^–13^ m^3^ molecule^−1^ s^−1^ at 500 K and are 1.03 × 10^–11^ and 1.04 × 10^–12^ cm^3^ molecule^−1^ s^−1^ at 1000 K, respectively. These values indicate that our computed rate constants agree well with the experimental results.

In summary, a comparison between our calculated rates by TST and VTST theories with the measured rates using several experimental techniques shows that our applied computational level, the UM06-2X/Aug-cc-pVTZ, is adequately precise in describing the kinetic of the CH_3_SH plus OH reaction.

#### Low-pressure limit rate constant and its behavior in the falloff regime

To study the pressure-dependent behavior of the rate constant for the CH_3_SH + OH reaction in the falloff range and low-pressure limit, the Rice–Ramsperger–Kassel–Marcus (RRKM) theory was applied with a weak collision approach. In the calculation of pressure-dependent rate constant the chemical activation mechanism was defined as follows:

1 $$CH_{3} SH + OH\mathop{\longrightarrow}^{K(T)}CR1$$

2 $$Cr1 + M\mathop{\longrightarrow}^{{k_{1} }}CR1* + M$$

3 $$Cr1* + M\mathop{\longrightarrow}^{{k_{ - 1} }}CR1 + M$$

4 $$Cr1*\mathop{\longrightarrow}^{{k_{2} }}CH_{3} S + H_{2} O$$

here M is (the third body) N_2_ molecule. Applying the steady-state approximation to the concentration of CR1*, the pressure-dependent rate of the CR1 → CH_3_S + H_2_O conversion is

5 $$k_{i} (T,p) = \frac{{k_{1} k_{2} [M]}}{{k_{ - 1} [M] + k_{2} }}$$

At high and low-pressure limits where [M] → ∞ and [M] → 0, k(T,p) is the first-order rate constant and the second-order rate constant, respectively. The expression ${k}_{i}(T,p)$ at the high and low-pressure limit is as follows:

6 $$k_{\infty } = \frac{{k_{1} k_{2} }}{{k_{ - 1} }}$$

7 $$k_{0} = k_{1} [M]$$

In Eq. (5), by a division of the nominator and the denominator into ${k}_{-1}\left[M\right]$ and substitute Eqs. (6) and (7) in it, gives:

8 $$k_{i} (T,p) = \frac{{k_{\infty } }}{{1 + \frac{{k_{\infty } }}{{k_{0} }}}}$$

Finally, to compute the pressure-dependent rate constant, the following equation is used:

9 $$k(T,p) = \kappa K(T)k_{i} (T,p)$$

where $\kappa$ and K(T) are tunneling correction and temperature dependent equilibrium constant, respectively.

The Eckart tunneling correction was used to achieve a reliable pressure-dependent rate constant. Nitrogen molecule is used as a third body because, in the atmosphere, the nitrogen molecule is the most abundant species. The average amount of energy transferred per collision $$\left\langle {\Delta E} \right\rangle$$, and Lenard Jones parameters including collision diameter, $$\sigma$$ (Å), and energy parameter, $${\raise0.7ex\hbox{$\varepsilon $} \!\mathord{\left/ {\vphantom {\varepsilon {k_{B} }}}\right.\kern-\nulldelimiterspace} \!\lower0.7ex\hbox{${k_{B} }$}}$$(K) are necessary for the calculation of the effect of pressure on the rate constant of reactions by the proposed theories. The amount of $$\left\langle {\Delta E} \right\rangle$$ during both up and down energy transfer collision for N2 bath gas is taken as 74 cm^−1^. Lenard-Jones parameters for CH_3_SH, OH, and N_2_ are 3.900 Å and 350.00 K for CH_3_SH, 2.750 Å, and 80.00 K for OH^55^. In the low-pressure limit where *P → 0*, k(T,p)/[N_2_] is called k_0_(T) that is a termolecular rate constant with the units of cm^6^ molecule^−2^ s^−1^. Also, $${k}_{0}$$(T) is called the pseudo-third-order rate constant. Our calculations show that k_0_(T) is 4.45 × 10^–27^, 4.44 × 10^–30^, 2.80 × 10^–31^, and 1.71 × 10^–31^ cm^−6^ molecule^−2^ s^−1^ at 150, 298, 500, and 700 K, respectively. Literature reviews show there are no experimental results for k_0_(T) of the CH_3_S + H_2_O production pathway.

**Figure 5:**
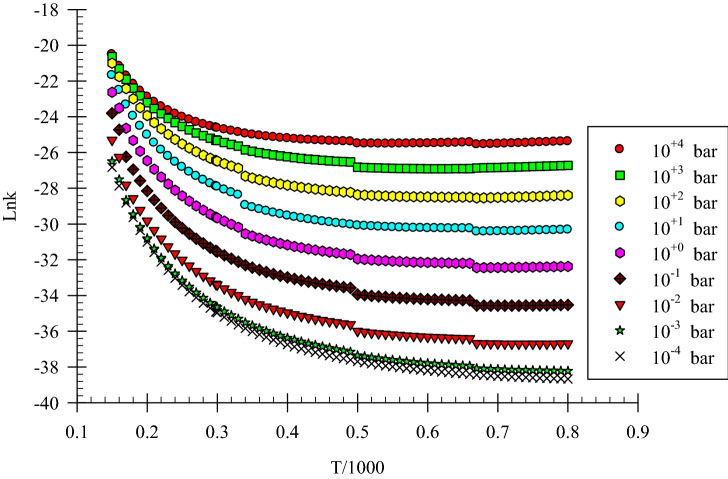
Pressure depended rate constant of the CH_3_SH + OH reaction for the R1 pathway.

Our computed rate constants in the temperature range of 150-800 K at the pressure range of 10^–4^–10^+4^ bar in the falloff regime are depicted in Fig. 5 and listed in Table S7. In the calculation of k(T,p), Eq. (8) shows that the ratio of k_∞_/k_0_ is important. Therefore, in the investigated pathway, with increasing pressure, the rate constant increases at a definite temperature in the mentioned temperature range (see Fig. 5). The k_∞_/k_0_ ratio is increasing with increasing temperature. For example, at the temperatures of 150, 298.15, 500, and 700 K, this ratio is 7.59 × 10^2^, 4.01 × 10^4^, 4.29 × 10^5^, and 1.30 × 10^6^, respectively. If the ratio of k(T,p)/k(T, 1 bar) is defined as the reduced rate constant, we can obtain better insight about the effect of pressure on this reaction at a constant temperature. The reduced values for P = 10^–3^, 0.1, 10 and 10^3^ bar are 2.09 × 10^–2^, 3.10 × 10^–1^, 257, and 7.32 at 150 K, and 6.79 × 10^–3^, 1.152 × 10^–1^, 5.66, and 75.20 at 298.15 K, respectively. These results showed that the rate constant increases with increasing pressure on the investigated temperature range.

### Fate of methanethiol in the atmosphere

As argued above, the pathways leading to form the CH_3_S + H_2_O and CH_2_SH + H_2_O products play key roles and act as the main sink for methanethiol. If the lifetime of methanethiol in the investigated elementary bimolecular title reaction is calculated, it can be predicted the fate of the CH_3_SH compound in good fashion. The computed lifetimes for methanethiol are listed in Table 5. The lifetime values are computed at 0–50 km height from the surface of the earth and in an environment of OH radicals. On one hand, as our results show, the rate constant of the reaction decreases with decreasing of pressure that is companied by altitude increasing. On the other hand, as Table 5 shows the concentration of hydroxyl radicals is independent of height. Therefore, the calculated lifetime in the atmosphere varies from 2.08 × 10^+6^ s^−1^ at 0 km to 2.25 × 10^+7^ s^−1^ at 50 km. The obtained lifetime is short. Thus, the hydrogen abstraction path from the SH group is a dominant mechanism in the atmosphere for the degradation of CH_3_SH species. The computed lifetime by the high-pressure limit rate constant is 2.26 × 10^+4^ s^−1^, which the lifetime obtained by the high-pressure limit rate is 100 times higher than the pressure-dependent rate constant at 0 km altitude.

**Table 5:** The rate constant of R1 pathway, OH concentration in different altitude, and lifetimes of CH_3_SH in atmospheric conditions.

*H* (km)^a^	*T* (K)	*P* (mbar)^a^	*k*^b^	[OH] (molecule (cm^−3^)^a^	Τ (s)^c^	$\frac{{k}_{\infty }}{k}$
0	290.2	1013	1.60E − 13	3.00E + 06	2.08E + 06	9.21E + 01
5	250.2	495.9	2.78E − 13	1.00E + 06	3.59E + 06	6.30E + 01
10	215.6	242.8	8.10E − 13	5.70E + 05	2.17E + 06	2.82E + 01
15	198.0	118.8	1.21E − 12	4.20E + 05	1.96E + 06	2.29E + 01
20	208.0	58.18	4.48E − 13	3.70E + 05	6.03E + 06	5.52E + 01
25	216.1	28.48	1.91E − 13	6.60E + 05	7.92E + 06	1.19E + 02
30	221.5	13.94	9.51E − 14	1.60E + 06	6.57E + 06	2.28E + 02
35	228.1	6.826	4.60E − 14	3.70E + 06	5.88E + 06	4.45E + 02
40	240.5	3.341	1.88E − 14	6.80E + 06	7.82E + 06	9.93E + 02
45	251.9	1.636	7.92E − 15	8.50E + 06	1.48E + 07	2.20E + 03
50	253.7	0.801	6.52E − 15	6.80E + 06	2.25E + 07	2.64E + 03

^a^The altitude (H), pressure (P), the temperature (T), and the OH concentration ([OH]) in this table are from Ref.^56^.

^b^k is the bimolecular rate constant in mentioned temperature and pressure.

^c^τ = $\frac{1}{k[OH]}$ is lifetime of CH_3_SH.

### Which path could be the main route?

**Figure 6:**
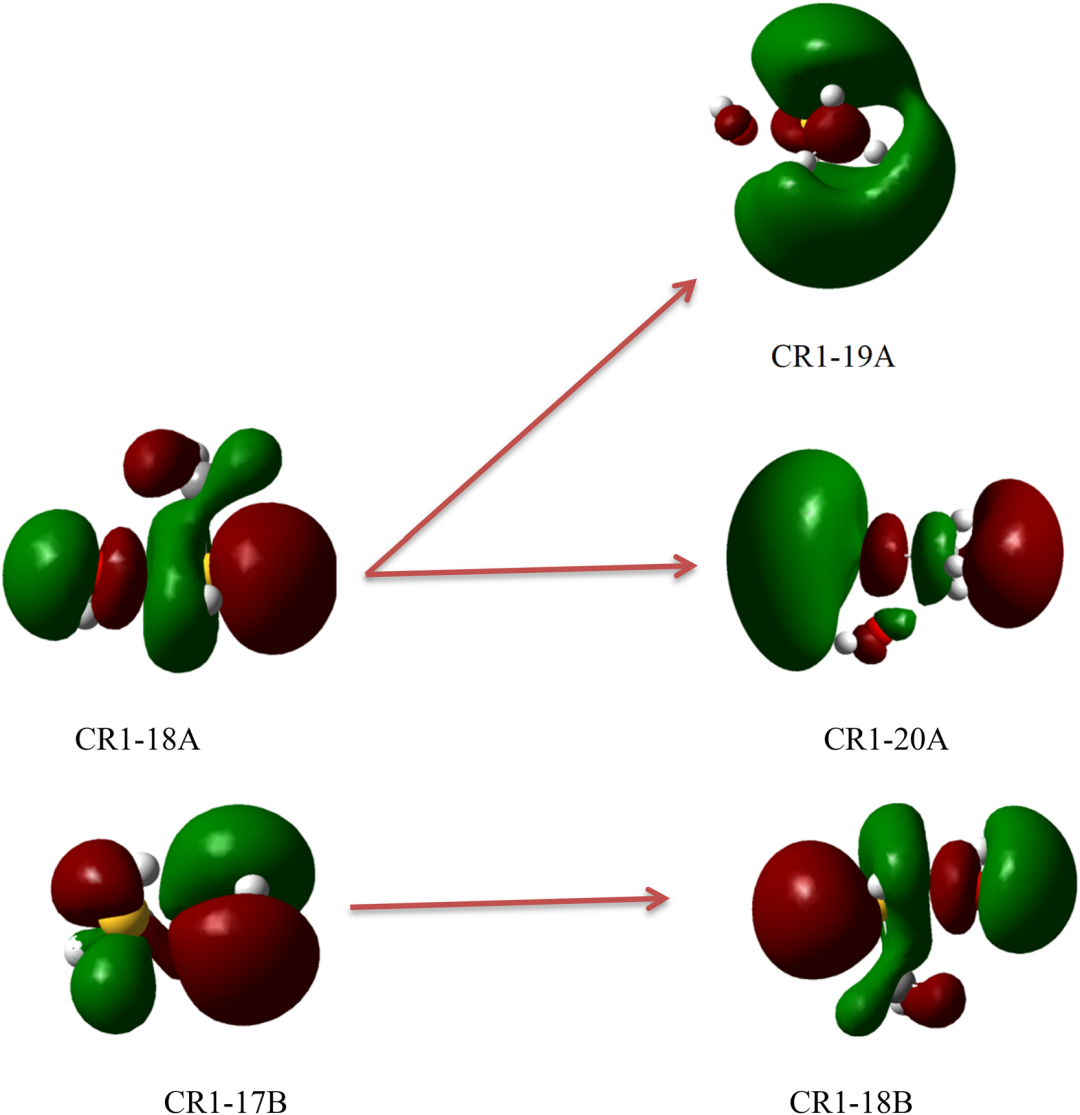
Ground state (17B and 18A) orbitals and the first excited state (18B, 19A, and 20A) orbitals of CR1.

**Figure 7:**
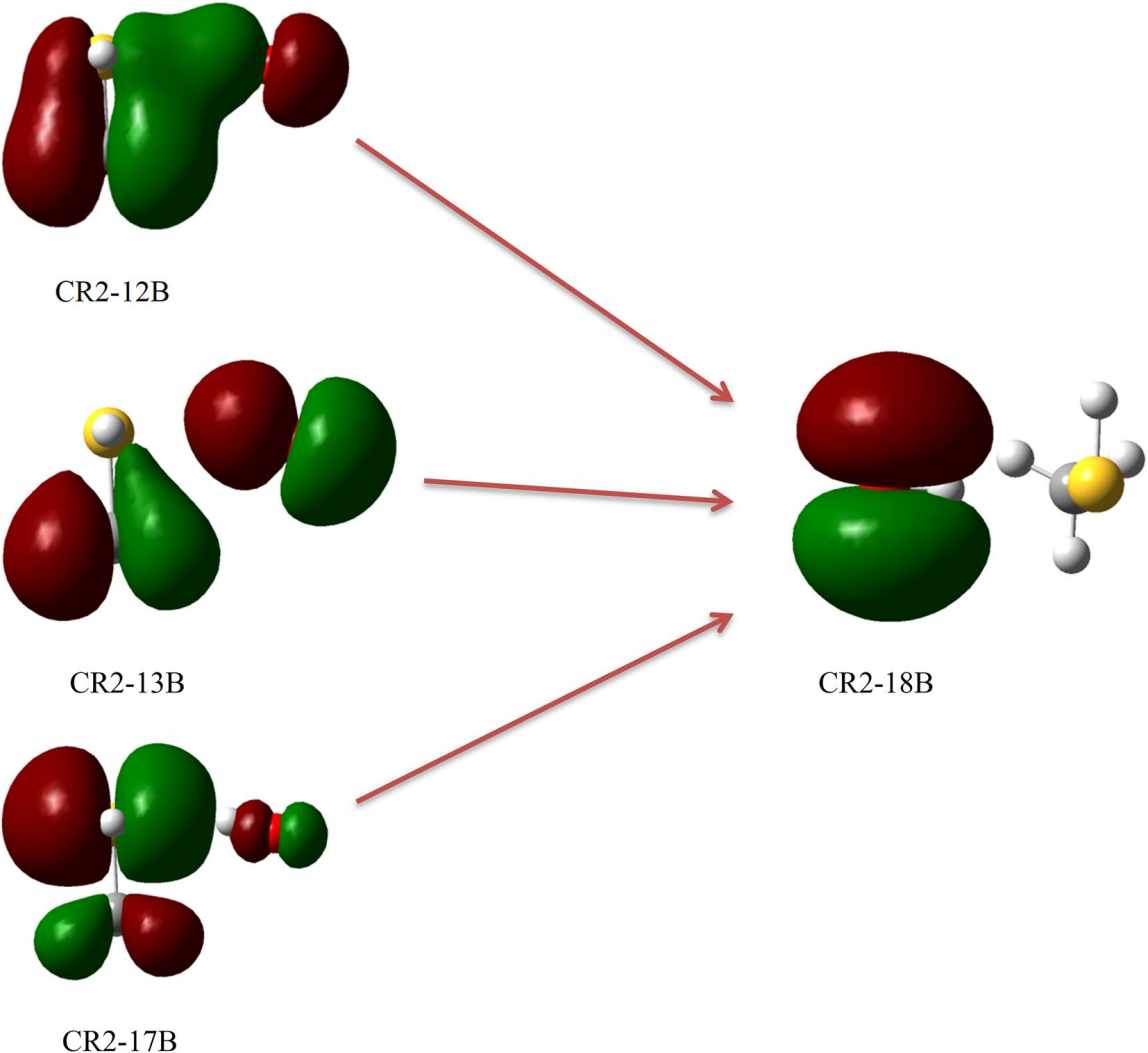
Ground state (12B, 13B, and 17B) and the second excited state (18B) orbital of CR2.

**Figure 8:**
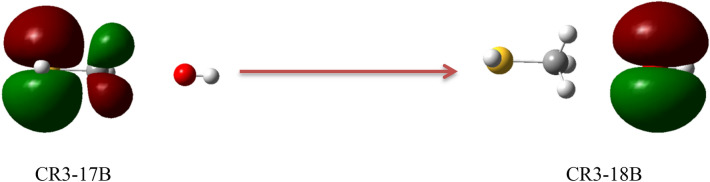
Ground state (17B) and the second excited state (18B) orbitals of CR3.

In this section, the photolysis of all complexes is argued, based on the TDDFT calculations in the gas phase. It is proved that the PBE0^57^ method has the most accurate results for the TDDFT calculations^58^. The geometries obtained at the MP2/aug-cc-pVTZ level are used for the TD-PBE0/aug-cc-pVTZ calculations. The obtained results are listed in Table 6. The ground state and excited state orbitals for prereacctive complexes are depicted in Figs. 6, 7 and 8 and for post-reactive complexes are shown in Figs. S2–S8. Also, we have performed similar calculations based on the TD-M06-2X/aug-cc-pVTZ//M06-2X/aug-cc-pVTZ level (see Table S8). The vertical excitation energy (*E*_*v*_), oscillator strength (*f*), and wavelength (λ) related to the first six excited states of all complexes are listed in Table 6. On the basis of Table 6, among the prereactive complexes, CR2 and CR3 are photolyzed more than CR1 due to comparing the first three excited state energies. Therefore, CR1 is more stable than CR2 and CR3 as a beginner of reactions in the atmosphere. It may relate to unpaired electrons of the sulfur atom and the radical electron of the oxygen atom. In comparison with S atom, the unpaired electrons (but radical electron) of OH radical due to the large electronegativity of the oxygen atom need for higher energy to excite. So, we only focus on the unpaired electrons of the S atom and radical electron. In the CR1 complex, some of the unpaired electrons of S atom are shared with the radical electron of OH moiety and they need to get large energy for excitation, but in other complexes, they are free for excitation. This result agrees with the computed relative energies of the mentioned complexes at the PMP2, Ub3lyp, UM06-2X, and UCCSD(T) methods in conjunction with augmented triple zeta basis sets. Because there is a bond between the oxygen atom of OH moiety and the atom S of CH_3_SH moiety with good strength and interaction (− 0.1080 e bohr^−3^ and 0.1259 e bohr^−5^). As we know, when excitation occurs, the electrons that are shared in a bond or an interaction can move to the upper layer, so the bond or interaction is broken. There is also good agreement with the kinetic results obtained from the experiments. The experimental results indicate that under the atmospheric conditions, especially under sunlight, the R1 pathway is the most probable path to happen. Therefore, we can conclude that the CR1 complex has the most possibility for the continuation of the reaction. Therefore, in the experiment the only production of the CH_3_S + H_2_O adduct is feasible.

**Table 6:** Excited state parameters of all pre-reactive complexes in the CH_3_SH + OH reaction computed at the TD-PBE0/aug-cc-pvtz level of theory.

Excited state number	CR1			CR2			CR3		
	E_v_	λ	*f*	E_v_	λ	*f*	E_v_	λ	*f*
1	3.21	386.29	0.0013	0.07	17,919.00	0.0000	0.06	21,559.22	0.0000
2	3.27	379.39	0.0046	2.35	527.18	0.0008	1.37	903.74	0.0040
3	3.75	330.22	0.0125	4.42	280.74	0.0013	3.89	322.13	0.0000
4	4.48	276.49	0.0083	5.10	243.26	0.0000	4.32	286.92	0.0009
5	4.73	261.90	0.0046	5.18	239.41	0.0002	4.81	257.61	0.0000
6	4.99	248.53	0.0037	5.57	222.69	0.0069	5.20	238.48	0.0068

Excited state number	Cp1			Cp2			Cp3		
	E_v_	λ	*f*	E_v_	λ	*f*	E_v_	λ	*f*
1	0.32	3917.49	0.0000	3.71	334.42	0.0032	3.14	394.92	0.0002
2	3.66	339.19	0.0015	4.34	285.88	0.0047	4.13	299.91	0.0008
3	3.86	321.44	0.0020	4.62	268.16	0.0368	4.87	254.79	0.0010
4	5.49	225.74	0.0009	4.82	256.99	0.0146	4.87	254.42	0.0064
5	5.76	215.30	0.0003	4.86	255.15	0.0335	5.29	234.35	0.1192
6	5.85	212.08	0.0187	5.21	237.81	0.0027	5.49	226.02	0.0031

Excited state number	Cp4			Cp5			Cp6		
	E_v_	λ	f	E_v_	λ	f	E_v_	λ	f
1	3.38	366.33	0.0000	0.06	20,647.38	0.0000	2.27	545.80	0.0005
2	4.30	288.21	0.0008	2.46	500.95	0.0030	4.98	249.02	0.0001
3	5.02	247.20	0.0022	4.00	310.06	0.0014	5.29	234.47	0.0391
4	5.22	237.63	0.0036	4.13	300.33	0.0009	5.56	222.94	0.0055
5	5.34	232.26	0.0002	5.64	219.89	0.0014	5.76	215.23	0.0026
6	5.52	224.73	0.0006	6.17	201.08	0.0000	5.80	213.90	0.0003

Excited state number	Cp7								
	E_v_	λ	f						
1	3.83	323.94	0.0030						
2	4.68	265.07	0.0158						
3	4.90	253.32	0.0004						
4	5.40	229.80	0.0003						
5	5.47	226.74	0.0003						
6	5.49	225.70	0.0161						

The units of vertical excitation energies (*E*_*v*_) and wavelength (λ) are eV and nm, respectively.

This does not mean that other reactions do not occur. On the other hand, hydrogen abstraction from the methyl group is somewhat kinetically feasible, under certain conditions, such as collisions with suitable energy and orientation, they may occur. For the second step reactions occurring from CP1 and CP3, CP1 is unstable than CP3, relating to the calculated vertical excitation energies. This unstability is related to both the radical electron and unpaired electrons of the atom S in CH_3_S. They require smaller energy for excitation (especially radical electron on S atom). So, the reaction between HSOH and CH_3_ is feasible than the CH_3_S + H_2_O reaction. Another reason for the possibility of the HSOH + CH_3_ reaction is the energy barrier of this reaction. For the reaction between CH_3_OH and HS (production of H_2_S + CH_2_OH, which starts from CP5), the Cp5 complex is also an unstable complex under the sunlight of the atmosphere. In the presence of sun rays, the electrons of the atom S in HS become excited, so the HS…CH_3_OH complex is splintered. Finally, for a bimolecular reaction with a high energy barrier and for multi-step reactions in which prereactive, post reactive, and intermediate complexes have low stability due to excitation, the sun's solar radiation not only has no important role in the supplying activation energy of that reaction but also prevent continuing the reaction. But, in the reactions with stable wells, sunlight acts as a significant source for activation energy.

For first step reactions with unstable prereactive complex, a collision with suitable energy and orientation may influence the reaction occurring, but for multi-step reactions with the same feature, the strong collisions with suitable orientations may lead to the reaction occurring. From the above statements, we concluded that the photo-oxidation of sulfur-containing compounds is important to the sulfur chemistry of the atmosphere. And we also concluded that many of the above-discussed paths, but the P1 production pathway, may occur under a condition that they complex is stable or be under certain collisions.

## Conclusion

The PES of the CH_3_SH + OH reaction was described on the doublet state in more detail using the single point calculations at the UCCSD(T)/aug-cc-pV(T + d)Z level, and the geometries, thermodynamic corrections, and zero points corrections at the MP2/aug-cc-pVTZ level. Therefore, the mechanisms and kinetics of the reaction were cleared using the mentioned levels. Our results showed that conventional hydrogen abstraction reaction from the SH functional group has more contribution than the others in the atmospheric degradation of methanethiol. Thus, on one hand, its pathway kinetically plays an important role. In another word, this path is a kinetic pathway. On the other hand, the obtained product from this path has more negative standard Gibbs free energy ($\Delta G^{0}$ = − 32.03 kcal/mol) than the others.

The electronic structure calculations along with the kinetic calculation showed that another favorable path after R1 was R2 production, which was involved hydrogen abstraction from the methyl functional group of methanethiol compound by hydroxyl radical.

On the basis of the computed PES, it was concluded that the main path of the reaction was barrier-less, so, this system is ideal to investigate pressure effect. Up to now pressure effect on the title reaction had remained obscure, so our results clear it. The chemical activation mechanism for the R1 pathway was confined to the P1(CH_3_S + H_2_O) reaction product. It was confirmed that the overall rate of title reaction depends on not only the temperature but also the pressure. Therefore, the importance of variation of pressure on the gas-phase reaction of CH_3_SH + OH was proved in the atmospheric condition. The negative temperature dependent rate constant was observed for barrierless pathways and positive for another, while the pressure-dependent rate constant for the main pathway was positive. A comparison of the computed rate constants with experimental results indicated that the UM06-2X method with the aug-cc-pVTZ basis set was a good case to obtain reliable pressure and temperature dependent rate constants.

The fate methyl mercaptan in the atmosphere was determined by using the computed pressure-dependent rate constants in different altitudes and also using the computed high-pressure limit rate constant.

Finally, It was shown that the excitations of complexes in the beginning and in the course of reactions could play a very important role in the atmospheric reactions.

## Supplementary information

Supplementary Information.
