# Supplementary material for: Atmospheric reaction of methyl mercaptan with hydroxyl radical as an acid rain primary agent
Source: Sci Rep. 2020 Oct 22;10:18081. doi: 10.1038/s41598-020-74767-6 (PMC7583294; doi:10.1038/s41598-020-74767-6)
Supplement: Supplementary file 1 — Supplementary Information. [file 41598_2020_74767_MOESM1_ESM.docx]

**Atmospheric reaction of methyl mercaptan with hydroxyl radical as an acid rain primary agent**

Hamed Douroudgari^*^, Morteza Vahedpour^*^, Samane Mohammadi

Department of Chemistry, University of Zanjan, PO Box 38791-45371, Zanjan, Iran

E-mail:  [douroudgari@znu.ac.ir](mailto:%20douroudgari@znu.ac.ir)

E-mail:  [vahed@znu.ac.ir](mailto:%20vahed@znu.ac.ir)

**Table S1**. Equilibrium constants (cm^3^ molecule^-1^) of initial complexes calculated at the UM06-2X/aug-cc-pVTZ + ZPE level in the temperature range of 150-3000 K.

**Table S2.** Total energies of all species in the CH_3_SH + OH reaction computed at different levels.

**Table** **S3**. Thermodynamic corrections of all species computed at the UMP2/aug-cc-pVTZ level.

**Table S4.** T1 diagnostic and < S^2^> values derived from the UCCSD(T)/aug-cc-pV(T+d)Z//UMP2/aug-cc-pVTZ and UMP2/aug-cc-pVTZ levels, respectively.

**Table S5**. High pressure limit rate constants (cm^3^ molecule^-1^ s^-1^) calculated by TST theory at the UM06-2X/aug-cc-pVTZ level for the P1 and P2 adducts through the R1 and R2 pathways.

**Table S6**. High pressure limit rate constants (cm^3^ molecule^-1^ s^-1^) calculated by TST theory at the UCCSD(T)/aug-cc-pV(T+d)Z (energies) + UMP2/aug-cc-pVTZ (partition functions and ZPEs) level for the P1 and P2 adducts through the R1 and R2 pathways.

**Table S7**. Pressure dependent rate constants calculated at the UM06-2X/aug-cc-pVTZ for R1 pathway.

**Table S8**. Excited state parameters of all complexes in the CH_3_SH + OH reaction computed at the TD-M06-2X/aug-cc-pVTZ level. (The geometries of all complexes obtained at the same level of theory, but the geometry of CP4 obtained at the M06-2X/6-311++g(3df,3pd)). The units of vertical excitation energies (*E_v_*) and wavelength (λ) are eV and nm, respectively.

**Figure S1**. Geometrical parameters of all species in the CH_3_SH + OH reaction calculated at the MP2/6-311++g(3df,3pd) level of theory (Bond lengths are in angstrom and angles are in degree).

**Figure S2.** Ground state and the second and third excited state orbitals of CP1.

**Figure S3.** Ground state and the first excited state orbitals of CP2.

**Figure S4.** Ground state and the first excited state orbitals of CP3.

**Figure S5.** Ground state and the second excited state orbitals of CP4.

**Figure S6.** Ground state and the second and third excited state orbitals of CP5.

**Figure S7.** Ground state and the first excited state orbitals of CP6.

**Figure S8.** Ground state and the first excited state orbitals of CP7.

| CR1 | | | | CR2 | | | | CR3 | | | |
| --- | --- | --- | --- | --- | --- | --- | --- | --- | --- | --- | --- |
| T/K | K | T/K | K | T/K | K | T/K | K | T/K | K | T/K | K |
| 150 | 1.10E-18 | 580 | 1.33E-23 | 150 | 6.48E-21 | 580 | 1.96E-23 | 150 | 1.30E-22 | 580 | 2.77E-22 |
| 160 | 3.83E-19 | 590 | 1.28E-23 | 160 | 3.53E-21 | 590 | 1.95E-23 | 160 | 1.23E-22 | 590 | 2.84E-22 |
| 170 | 1.52E-19 | 600 | 1.23E-23 | 170 | 2.08E-21 | 600 | 1.94E-23 | 170 | 1.18E-22 | 600 | 2.92E-22 |
| 180 | 6.72E-20 | 610 | 1.18E-23 | 180 | 1.30E-21 | 610 | 1.93E-23 | 180 | 1.15E-22 | 610 | 3.00E-22 |
| 190 | 3.25E-20 | 620 | 1.14E-23 | 190 | 8.65E-22 | 620 | 1.92E-23 | 190 | 1.12E-22 | 620 | 3.08E-22 |
| 200 | 1.70E-20 | 630 | 1.10E-23 | 200 | 6.01E-22 | 630 | 1.91E-23 | 200 | 1.11E-22 | 630 | 3.16E-22 |
| 210 | 9.45E-21 | 640 | 1.07E-23 | 210 | 4.34E-22 | 640 | 1.91E-23 | 210 | 1.11E-22 | 640 | 3.25E-22 |
| 220 | 5.57E-21 | 650 | 1.03E-23 | 220 | 3.25E-22 | 650 | 1.91E-23 | 220 | 1.11E-22 | 650 | 3.33E-22 |
| 230 | 3.45E-21 | 660 | 1.00E-23 | 230 | 2.50E-22 | 660 | 1.90E-23 | 230 | 1.12E-22 | 660 | 3.42E-22 |
| 240 | 2.23E-21 | 670 | 9.76E-24 | 240 | 1.98E-22 | 670 | 1.90E-23 | 240 | 1.13E-22 | 670 | 3.51E-22 |
| 250 | 1.50E-21 | 680 | 9.52E-24 | 250 | 1.60E-22 | 680 | 1.91E-23 | 250 | 1.14E-22 | 680 | 3.60E-22 |
| 260 | 1.04E-21 | 690 | 9.29E-24 | 260 | 1.32E-22 | 690 | 1.91E-23 | 260 | 1.16E-22 | 690 | 3.69E-22 |
| 270 | 7.47E-22 | 700 | 9.07E-24 | 270 | 1.11E-22 | 700 | 1.91E-23 | 270 | 1.18E-22 | 700 | 3.78E-22 |
| 280 | 5.50E-22 | 710 | 8.88E-24 | 280 | 9.46E-23 | 710 | 1.92E-23 | 280 | 1.21E-22 | 710 | 3.88E-22 |
| 290 | 4.14E-22 | 720 | 8.70E-24 | 290 | 8.19E-23 | 720 | 1.92E-23 | 290 | 1.23E-22 | 720 | 3.97E-22 |
| 298 | 3.35E-22 | 730 | 8.53E-24 | 298 | 7.36E-23 | 730 | 1.93E-23 | 298 | 1.26E-22 | 730 | 4.07E-22 |
| 298.15 | 3.34E-22 | 740 | 8.38E-24 | 298.15 | 7.35E-23 | 740 | 1.94E-23 | 298.15 | 1.26E-22 | 740 | 4.17E-22 |
| 300 | 3.19E-22 | 750 | 8.24E-24 | 300 | 7.18E-23 | 750 | 1.95E-23 | 300 | 1.26E-22 | 750 | 4.27E-22 |
| 310 | 2.50E-22 | 760 | 8.10E-24 | 310 | 6.37E-23 | 760 | 1.96E-23 | 310 | 1.29E-22 | 760 | 4.37E-22 |
| 320 | 2.00E-22 | 770 | 7.98E-24 | 320 | 5.71E-23 | 770 | 1.97E-23 | 320 | 1.33E-22 | 770 | 4.48E-22 |
| 330 | 1.62E-22 | 780 | 7.87E-24 | 330 | 5.16E-23 | 780 | 1.98E-23 | 330 | 1.36E-22 | 780 | 4.58E-22 |
| 340 | 1.34E-22 | 790 | 7.76E-24 | 340 | 4.71E-23 | 790 | 2.00E-23 | 340 | 1.40E-22 | 790 | 4.69E-22 |
| 350 | 1.11E-22 | 800 | 7.67E-24 | 350 | 4.33E-23 | 800 | 2.01E-23 | 350 | 1.44E-22 | 800 | 4.80E-22 |
| 360 | 9.41E-23 | 900 | 7.04E-24 | 360 | 4.00E-23 | 900 | 2.19E-23 | 360 | 1.48E-22 | 900 | 5.98E-22 |
| 370 | 8.04E-23 | 1000 | 6.82E-24 | 370 | 3.73E-23 | 1000 | 2.44E-23 | 370 | 1.52E-22 | 1000 | 7.34E-22 |
| 380 | 6.93E-23 | 1100 | 6.85E-24 | 380 | 3.49E-23 | 1100 | 2.75E-23 | 380 | 1.56E-22 | 1100 | 8.87E-22 |
| 390 | 6.04E-23 | 1200 | 7.06E-24 | 390 | 3.29E-23 | 1200 | 3.11E-23 | 390 | 1.61E-22 | 1200 | 1.06E-21 |
| 400 | 5.30E-23 | 1300 | 7.39E-24 | 400 | 3.12E-23 | 1300 | 3.53E-23 | 400 | 1.66E-22 | 1300 | 1.25E-21 |
| 410 | 4.70E-23 | 1400 | 7.84E-24 | 410 | 2.96E-23 | 1400 | 4.02E-23 | 410 | 1.71E-22 | 1400 | 1.46E-21 |
| 420 | 4.19E-23 | 1500 | 8.38E-24 | 420 | 2.83E-23 | 1500 | 4.57E-23 | 420 | 1.76E-22 | 1500 | 1.69E-21 |
| 430 | 3.76E-23 | 1600 | 9.01E-24 | 430 | 2.71E-23 | 1600 | 5.18E-23 | 430 | 1.81E-22 | 1600 | 1.95E-21 |
| 440 | 3.40E-23 | 1700 | 9.73E-24 | 440 | 2.61E-23 | 1700 | 5.87E-23 | 440 | 1.86E-22 | 1700 | 2.22E-21 |
| 450 | 3.09E-23 | 1800 | 1.05E-23 | 450 | 2.52E-23 | 1800 | 6.63E-23 | 450 | 1.92E-22 | 1800 | 2.52E-21 |
| 460 | 2.83E-23 | 1900 | 1.14E-23 | 460 | 2.44E-23 | 1900 | 7.47E-23 | 460 | 1.97E-22 | 1900 | 2.84E-21 |
| 470 | 2.60E-23 | 2000 | 1.24E-23 | 470 | 2.37E-23 | 2000 | 8.39E-23 | 470 | 2.03E-22 | 2000 | 3.18E-21 |
| 480 | 2.40E-23 | 2100 | 1.35E-23 | 480 | 2.30E-23 | 2100 | 9.40E-23 | 480 | 2.09E-22 | 2100 | 3.55E-21 |
| 490 | 2.23E-23 | 2200 | 1.46E-23 | 490 | 2.25E-23 | 2200 | 1.05E-22 | 490 | 2.15E-22 | 2200 | 3.94E-21 |
| 500 | 2.08E-23 | 2300 | 1.59E-23 | 500 | 2.20E-23 | 2300 | 1.17E-22 | 500 | 2.21E-22 | 2300 | 4.36E-21 |
| 510 | 1.94E-23 | 2400 | 1.72E-23 | 510 | 2.15E-23 | 2400 | 1.30E-22 | 510 | 2.28E-22 | 2400 | 4.80E-21 |
| 520 | 1.82E-23 | 2500 | 1.87E-23 | 520 | 2.11E-23 | 2500 | 1.44E-22 | 520 | 2.34E-22 | 2500 | 5.27E-21 |
| 530 | 1.72E-23 | 2600 | 2.02E-23 | 530 | 2.08E-23 | 2600 | 1.59E-22 | 530 | 2.41E-22 | 2600 | 5.77E-21 |
| 540 | 1.62E-23 | 2700 | 2.19E-23 | 540 | 2.05E-23 | 2700 | 1.76E-22 | 540 | 2.48E-22 | 2700 | 6.29E-21 |
| 550 | 1.54E-23 | 2800 | 2.37E-23 | 550 | 2.02E-23 | 2800 | 1.94E-22 | 550 | 2.55E-22 | 2800 | 6.84E-21 |
| 560 | 1.46E-23 | 2900 | 2.55E-23 | 560 | 2.00E-23 | 2900 | 2.12E-22 | 560 | 2.62E-22 | 2900 | 7.42E-21 |
| 570 | 1.39E-23 | 3000 | 2.76E-23 | 570 | 1.98E-23 | 3000 | 2.33E-22 | 570 | 2.69E-22 | 3000 | 8.03E-21 |

**Table S1**. Equilibrium constants (cm^3^ molecule^-1^) of initial complexes calculated at the UM06-2X/aug-cc-pVTZ + ZPE level in the temperature range of 150-3000 K.

| Species | MP2 | | PMP2 | | M062X | | CCSD(T) | | B3lyp |
| --- | --- | --- | --- | --- | --- | --- | --- | --- | --- |
|  | Aug-cc-pVTZ | 6-311++ g(3df,3pd) | Aug-cc-pVTZ | 6-311++ g(3df,3pd) | Aug-cc-pVTZ | 6-311++ g(3df,3pd) | Aug-cc-pVT(Z+ d) | 6-311++ g(3df,3pd) | 6-311++ g(3df,3pd) |
| OH | -75.6263 | -75.6208 | -75.6283 | -75.6227 | -75.7338 | -75.7303 | -75.6456 | -75.6208 | -75.7662 |
| CH_3_SH | -438.1282 | -438.1160 | -438.1282 | -438.1160 | -438.6901 | -438.6861 | -438.1803 | -438.1160 | -438.7536 |
| R | -513.7546 | -513.7368 | -513.7566 | -513.7386 | -514.4239 | -514.4164 | -513.8259 | -513.7368 | -514.5199 |
| CR1 | -513.7635 | -513.7456 | -513.7691 | -513.7441 | -514.4362 | -514.4290 | -513.8330 | -513.7456 | -514.5371 |
| CR2 | -513.7620 | -513.7443 | -513.7640 | -513.7445 | -514.4316 | -514.4244 | -513.8330 | -513.7443 | -514.5264 |
| CR3 | -513.7562 | -513.7384 | -513.7581 | -513.7400 | -514.4253 | -514.4181 | -513.8276 | -513.7384 | -514.5205 |
| TS1 | -513.7529 | -513.7346 | -513.7573 | -513.7335 | -514.4859 | -514.4201 | -513.8287 | -513.7346 | -514.5302 |
| TS2 | -513.7465 | -513.7291 | -513.7511 | -513.7324 | -514.4651 | -514.4145 | -513.8225 | -513.7291 | -514.5231 |
| TS3 | -513.7434 | -513.7252 | -513.7498 | -513.7261 | -514.4202 | -514.4066 | -513.8142 | -513.7252 | -514.5120 |
| TS4 | -513.7346 | -513.7159 | -513.7380 | -513.7143 | - | -514.3926 | -513.7993 | -513.7159 | - |
| TS5 | -513.7301 | -513.7120 | -513.7358 | -513.7130 | -514.4589 | -514.3940 | -513.8004 | -513.7120 | - |
| TS6 | -513.7037 | -513.6837 | -513.7180 | -513.6842 | -514.4629 | -514.3753 | -513.7842 | -513.6837 | -514.4869 |
| TS7 | -513.7341 | -513.7166 | -513.7412 | -513.7086 | -514.4469 | - | -513.8082 | -513.7166 | -514.5111 |
| TS8 | -513.7098 | -513.6898 | -513.7221 | -513.6889 | -514.4276 | -514.3774 | -513.7843 | -513.6898 | -514.4901 |
| TS9 | -513.7701 | -513.7533 | -513.7753 | -513.7515 | -514.4218 | -514.4335 | -513.8376 | -513.7533 | -514.5393 |
| TS10 | -513.7191 | -513.7028 | -513.7316 | -513.7020 | -514.4133 | -514.3828 | -513.7913 | -513.7028 | -514.4840 |
| CP1 | -513.8215 | -513.8038 | -513.8237 | -513.7441 | -514.3994 | -514.4782 | -513.8852 | -513.8038 | -514.5786 |
| CP2 | -513.7998 | -513.7841 | -513.8027 | -513.7864 | -514.4000 | -514.4586 | -513.8659 | -513.7841 | -514.5607 |
| CP3 | -513.7505 | -513.7325 | -513.7525 | -513.7271 | -514.3837 | - | -513.8184 | -513.7325 | -514.5138 |
| CP4 | -513.7386 | -513.7199 | -513.7386 | -513.2473 | - | -514.3932 | -513.7985 | -513.7199 | -514.4893 |
| CP5 | -513.7908 | -513.7735 | -513.7930 | -513.7749 | -514.3860 | -514.4514 | -513.8570 | -513.7735 | - |
| CP6 | -513.7924 | -513.7782 | -513.7976 | -513.7838 | - | -514.4584 | -513.8666 | -513.7782 | -514.5665 |
| CP7 | -513.7798 | -513.7638 | -513.7818 | -513.7672 | -514.3898 | -514.4403 | -513.8467 | -513.7638 | -514.5440 |
| P1(CH_3_S+H_2_O) | -513.8139 | -513.7962 | -513.8162 | -513.7965 | -514.4783 | -514.4702 | -513.8778 | -513.7962 | -514.5730 |
| P2(CH_2_SH+H_2_O) | -513.7939 | -513.7782 | -513.7968 | -513.7786 | -514.4585 | -514.4516 | -513.8601 | -513.7782 | -514.5570 |
| P3(CH_3_+HSOH) | -513.7476 | -513.7295 | -513.7495 | -513.7291 | -514.4150 | -514.4077 | -513.8155 | -513.7295 | -514.5125 |
| P4(CH_3_OSH+H) | -513.7381 | -513.7195 | -513.7381 | -513.7195 | -514.4001 | -514.3933 | -513.7979 | -513.7195 | -514.4709 |
| P5(CH_3_OH+SH) | -513.7893 | -513.7723 | -513.7915 | -513.7733 | -514.4566 | -514.4489 | -513.8555 | -513.7723 | -514.5520 |
| P6(CH_4_+HSO) | -513.7894 | -513.7752 | -513.7945 | -513.7752 | -514.4604 | -514.4556 | -513.8638 | -513.7752 | -514.5660 |
| P7(CH_2_OH+H_2_S) | -513.7752 | -513.7591 | -513.7752 | -513.7595 | -514.4431 | -514.4362 | -513.8423 | -513.7591 | -514.5415 |
| P8(CH_2_S+H+H_2_O) | -513.7273 | -513.7109 | -513.7273 | -513.7109 | -514.3835 | -514.3764 | -513.7866 | -513.7109 | -514.4784 |
| CH_3_S | -437.4849 | -437.4719 | -437.4872 | -437.4722 | -438.0483 | -438.0432 | -437.5355 | -437.4719 | -438.1085 |
| H_2_O | -76.3290 | -76.3243 | -76.3290 | -76.3243 | -76.4301 | -76.4270 | -76.3423 | -76.3243 | -76.4645 |
| CH_2_SH | -437.4649 | -437.4539 | -437.4678 | -437.4543 | -438.0284 | -438.0246 | -437.5177 | -437.4539 | -438.0925 |
| CH_3_ | -39.7383 | -39.7362 | -39.7403 | -39.7358 | -39.8254 | -39.8239 | -39.7636 | -39.7362 | -39.8584 |
| HSOH | -474.0092 | -473.9933 | -474.0092 | -473.9933 | -474.5896 | -474.5838 | -474.0519 | -473.9933 | -474.6541 |
| CH_3_OSH | -513.2383 | -513.2197 | -513.2383 | -513.2197 | -513.9018 | -513.8951 | -513.2981 | -513.2197 | -513.9687 |
| H | -0.4998 | -0.4998 | -0.4998 | -0.4998 | -0.4982 | -0.4982 | -0.4998 | -0.4998 | -0.5023 |
| CH_3_OH | -115.5290 | -115.5220 | -115.5290 | -115.5220 | -115.7178 | -115.7140 | -115.5624 | -115.5220 | -115.7743 |
| SH | -398.2603 | -398.2503 | -398.2625 | -398.2513 | -398.7388 | -398.7349 | -398.2932 | -398.2503 | -398.7777 |
| CH_4_ | -40.4145 | -40.4120 | -40.4145 | -40.4120 | -40.5017 | -40.5005 | -40.4409 | -40.4120 | -40.5374 |
| HSO | -473.3749 | -473.3632 | -473.3801 | -473.3632 | -473.9587 | -473.9551 | -473.4229 | -473.3632 | -474.0286 |
| CH_2_OH | -114.8663 | -114.8597 | -114.8663 | -114.8601 | -115.0561 | -115.0523 | -114.8989 | -114.8597 | -115.1117 |
| H_2_S | -398.9088 | -398.8994 | -398.9088 | -398.8994 | -399.3869 | -399.3840 | -398.9435 | -398.8994 | -399.4298 |
| CH_2_S | -436.8985 | -436.8868 | -436.8985 | -436.8868 | -437.4552 | -437.4512 | -436.9444 | -436.8868 | -437.5117 |

**Table S2.** Total energies of all species in the CH_3_SH + OH reaction computed at different levels.

| Species | *Eº*(Hartree) | *Hº*(Hartree) | *Gº*(Hartree) | *Sº*(cal/Kmol) |
| --- | --- | --- | --- | --- |
| R | 0.0110 | 0.0119 | -0.0083 | 42.5580 |
| CR1 | 0.0503 | 0.0513 | 0.0225 | 60.5110 |
| CR2 | 0.0613 | 0.0632 | 0.0143 | 103.0690 |
| CR3 | 0.0658 | 0.0667 | 0.0325 | 72.0980 |
| TS1 | 0.0641 | 0.0650 | 0.0266 | 80.8780 |
| TS2 | 0.0637 | 0.0646 | 0.0215 | 90.7000 |
| TS3 | 0.0612 | 0.0621 | 0.0260 | 75.9950 |
| TS4 | 0.0587 | 0.0596 | 0.0242 | 74.5550 |
| TS5 | 0.0628 | 0.0638 | 0.0291 | 72.8730 |
| TS6 | 0.0593 | 0.0602 | 0.0262 | 71.6380 |
| TS7 | 0.0626 | 0.0636 | 0.0295 | 71.6470 |
| TS8 | 0.0635 | 0.0644 | 0.0286 | 75.3830 |
| TS9 | 0.0579 | 0.0589 | 0.0225 | 76.6760 |
| TS10 | 0.0629 | 0.0638 | 0.0283 | 74.8060 |
| CP1 | 0.0595 | 0.0604 | 0.0248 | 74.8610 |
| CP2 | 0.0581 | 0.0590 | 0.0197 | 82.7680 |
| CP3 | 0.0674 | 0.0683 | 0.0292 | 82.3290 |
| CP4 | 0.0637 | 0.0646 | 0.0245 | 84.4860 |
| CP5 | 0.0622 | 0.0631 | 0.0203 | 90.1630 |
| CP6 | 0.0599 | 0.0608 | 0.0215 | 82.7540 |
| CP7 | 0.0679 | 0.0688 | 0.0257 | 90.8640 |
| P1(CH_3_S+H_2_O) | 0.0653 | 0.0663 | 0.0257 | 85.4480 |
| P2(CH_2_SH+H_2_O) | 0.0624 | 0.0633 | 0.0218 | 87.4250 |
| P3(CH_3_+HSOH) | 0.0637 | 0.0656 | 0.0151 | 106.1460 |
| P4(CH_3_OSH+H) | 0.0603 | 0.0622 | 0.0103 | 109.1430 |
| P5(CH_3_OH+SH) | 0.0593 | 0.0612 | 0.0097 | 108.3510 |
| P6(CH_4_+HSO) | 0.0584 | 0.0603 | 0.0154 | 94.4970 |
| P7(CH_2_OH+H_2_S) | 0.0639 | 0.0658 | 0.0169 | 102.8890 |
| P8(CH_2_S+H+H_2_O) | 0.0624 | 0.0643 | 0.0135 | 106.9180 |

**Table** **S3**. Thermodynamic corrections of all species computed at the UMP2/aug-cc-pVTZ level.

| Species | < S2>_MP2_ | T1 diagnostic |
| --- | --- | --- |
| CR1 | 0.79 | 0.0210 |
| CR2 | 0.76 | 0.0101 |
| CR3 | 0.76 | 0.0098 |
| TS1 | 0.78 | 0.0274 |
| TS2 | 0.78 | 0.0259 |
| TS3 | 0.82 | 0.0207 |
| TS4 | 0.81 | 0.0183 |
| TS5 | 0.91 | 0.0199 |
| TS6 | 0.80 | 0.0306 |
| TS7 | 0.90 | 0.0258 |
| TS8 | 0.79 | 0.0257 |
| TS9 | 0.79 | 0.0169 |
| TS10 | 0.10 | 0.0365 |
| CP1 | 0.76 | 0.0120 |
| CP2 | 0.77 | 0.0173 |
| CP3 | 0.76 | 0.0122 |
| CP4 | 0.75 | 0.0123 |
| CP5 | 0.76 | 0.0105 |
| CP6 | 0.78 | 0.0208 |
| CP7 | 0.76 | 0.0143 |

**Table S4.** T1 diagnostic and < S^2^> values derived from the UCCSD(T)/aug-cc-pV(T+d)Z//UMP2/aug-cc-pVTZ and UMP2/aug-cc-pVTZ levels, respectively.

| R1 | | | | R2 | | | |
| --- | --- | --- | --- | --- | --- | --- | --- |
| T/K | k | T/K | k | T/K | k | T/K | k |
| 150 | 6.74E-11 | 580 | 1.67E-11 | 150 | 8.34E-14 | 580 | 1.10E-12 |
| 160 | 5.29E-11 | 590 | 1.71E-11 | 160 | 8.97E-14 | 590 | 1.16E-12 |
| 170 | 4.30E-11 | 600 | 1.74E-11 | 170 | 9.65E-14 | 600 | 1.21E-12 |
| 180 | 3.60E-11 | 610 | 1.77E-11 | 180 | 1.04E-13 | 610 | 1.27E-12 |
| 190 | 3.09E-11 | 620 | 1.81E-11 | 190 | 1.12E-13 | 620 | 1.33E-12 |
| 200 | 2.71E-11 | 630 | 1.85E-11 | 200 | 1.20E-13 | 630 | 1.39E-12 |
| 210 | 2.42E-11 | 640 | 1.88E-11 | 210 | 1.29E-13 | 640 | 1.45E-12 |
| 220 | 2.19E-11 | 650 | 1.92E-11 | 220 | 1.39E-13 | 650 | 1.51E-12 |
| 230 | 2.02E-11 | 660 | 1.96E-11 | 230 | 1.49E-13 | 660 | 1.58E-12 |
| 240 | 1.87E-11 | 670 | 2.01E-11 | 240 | 1.60E-13 | 670 | 1.65E-12 |
| 250 | 1.76E-11 | 680 | 2.05E-11 | 250 | 1.71E-13 | 680 | 1.72E-12 |
| 260 | 1.67E-11 | 690 | 2.09E-11 | 260 | 1.83E-13 | 690 | 1.79E-12 |
| 270 | 1.59E-11 | 700 | 2.14E-11 | 270 | 1.96E-13 | 700 | 1.87E-12 |
| 280 | 1.53E-11 | 710 | 2.18E-11 | 280 | 2.10E-13 | 710 | 1.95E-12 |
| 290 | 1.48E-11 | 720 | 2.23E-11 | 290 | 2.24E-13 | 720 | 2.03E-12 |
| 298 | 1.44E-11 | 730 | 2.28E-11 | 298 | 2.36E-13 | 730 | 2.12E-12 |
| 298.15 | 1.44E-11 | 740 | 2.33E-11 | 298.15 | 2.36E-13 | 740 | 2.20E-12 |
| 300 | 1.44E-11 | 750 | 2.38E-11 | 300 | 2.39E-13 | 750 | 2.29E-12 |
| 310 | 1.40E-11 | 760 | 2.43E-11 | 310 | 2.55E-13 | 760 | 2.38E-12 |
| 320 | 1.38E-11 | 770 | 2.49E-11 | 320 | 2.71E-13 | 770 | 2.48E-12 |
| 330 | 1.35E-11 | 780 | 2.54E-11 | 330 | 2.89E-13 | 780 | 2.58E-12 |
| 340 | 1.34E-11 | 790 | 2.60E-11 | 340 | 3.07E-13 | 790 | 2.68E-12 |
| 350 | 1.33E-11 | 800 | 2.65E-11 | 350 | 3.27E-13 | 800 | 2.78E-12 |
| 360 | 1.32E-11 | 900 | 3.30E-11 | 360 | 3.47E-13 | 900 | 4.00E-12 |
| 370 | 1.31E-11 | 1000 | 4.09E-11 | 370 | 3.68E-13 | 1000 | 5.60E-12 |
| 380 | 1.31E-11 | 1100 | 5.03E-11 | 380 | 3.90E-13 | 1100 | 7.64E-12 |
| 390 | 1.31E-11 | 1200 | 6.14E-11 | 390 | 4.14E-13 | 1200 | 1.02E-11 |
| 400 | 1.32E-11 | 1300 | 7.45E-11 | 400 | 4.38E-13 | 1300 | 1.34E-11 |
| 410 | 1.32E-11 | 1400 | 8.96E-11 | 410 | 4.63E-13 | 1400 | 1.72E-11 |
| 420 | 1.33E-11 | 1500 | 1.07E-10 | 420 | 4.90E-13 | 1500 | 2.18E-11 |
| 430 | 1.34E-11 | 1600 | 1.27E-10 | 430 | 5.18E-13 | 1600 | 2.73E-11 |
| 440 | 1.35E-11 | 1700 | 1.49E-10 | 440 | 5.47E-13 | 1700 | 3.37E-11 |
| 450 | 1.37E-11 | 1800 | 1.74E-10 | 450 | 5.77E-13 | 1800 | 4.12E-11 |
| 460 | 1.38E-11 | 1900 | 2.03E-10 | 460 | 6.09E-13 | 1900 | 4.99E-11 |
| 470 | 1.40E-11 | 2000 | 2.34E-10 | 470 | 6.42E-13 | 2000 | 5.98E-11 |
| 480 | 1.42E-11 | 2100 | 2.70E-10 | 480 | 6.76E-13 | 2100 | 7.11E-11 |
| 490 | 1.44E-11 | 2200 | 3.08E-10 | 490 | 7.12E-13 | 2200 | 8.39E-11 |
| 500 | 1.46E-11 | 2300 | 3.51E-10 | 500 | 7.49E-13 | 2300 | 9.83E-11 |
| 510 | 1.48E-11 | 2400 | 3.98E-10 | 510 | 7.87E-13 | 2400 | 1.14E-10 |
| 520 | 1.50E-11 | 2500 | 4.50E-10 | 520 | 8.28E-13 | 2500 | 1.32E-10 |
| 530 | 1.53E-11 | 2600 | 5.06E-10 | 530 | 8.70E-13 | 2600 | 1.52E-10 |
| 540 | 1.56E-11 | 2700 | 5.67E-10 | 540 | 9.13E-13 | 2700 | 1.74E-10 |
| 550 | 1.58E-11 | 2800 | 6.33E-10 | 550 | 9.58E-13 | 2800 | 1.99E-10 |
| 560 | 1.61E-11 | 2900 | 7.05E-10 | 560 | 1.01E-12 | 2900 | 2.25E-10 |
| 570 | 1.64E-11 | 3000 | 7.82E-10 | 570 | 1.05E-12 | 3000 | 2.54E-10 |

**Table S5**. High pressure limit rate constants (cm^3^ molecule^-1^ s^-1^) calculated by TST theory at the UM06-2X/aug-cc-pVTZ level for the P1 and P2 adducts through the R1 and R2 pathways.

| R1 | | | | R2 | | | |
| --- | --- | --- | --- | --- | --- | --- | --- |
| T/K | k | T/K | k | T/K | k | T/K | k |
| 150 | 2.76E-10 | 580 | 1.08E-11 | 150 | 2.10E-13 | 580 | 7.90E-13 |
| 160 | 1.93E-10 | 590 | 1.08E-11 | 160 | 2.13E-13 | 590 | 8.15E-13 |
| 170 | 1.42E-10 | 600 | 1.08E-11 | 170 | 2.17E-13 | 600 | 8.41E-13 |
| 180 | 1.08E-10 | 610 | 1.08E-11 | 180 | 2.21E-13 | 610 | 8.68E-13 |
| 190 | 8.55E-11 | 620 | 1.08E-11 | 190 | 2.26E-13 | 620 | 8.95E-13 |
| 200 | 6.93E-11 | 630 | 1.09E-11 | 200 | 2.32E-13 | 630 | 9.23E-13 |
| 210 | 5.74E-11 | 640 | 1.09E-11 | 210 | 2.38E-13 | 640 | 9.52E-13 |
| 220 | 4.86E-11 | 650 | 1.09E-11 | 220 | 2.44E-13 | 650 | 9.81E-13 |
| 230 | 4.19E-11 | 660 | 1.10E-11 | 230 | 2.51E-13 | 660 | 1.01E-12 |
| 240 | 3.66E-11 | 670 | 1.10E-11 | 240 | 2.59E-13 | 670 | 1.04E-12 |
| 250 | 3.24E-11 | 680 | 1.11E-11 | 250 | 2.67E-13 | 680 | 1.07E-12 |
| 260 | 2.91E-11 | 690 | 1.12E-11 | 260 | 2.75E-13 | 690 | 1.11E-12 |
| 270 | 2.64E-11 | 700 | 1.12E-11 | 270 | 2.84E-13 | 700 | 1.14E-12 |
| 280 | 2.41E-11 | 710 | 1.13E-11 | 280 | 2.93E-13 | 710 | 1.17E-12 |
| 290 | 2.22E-11 | 720 | 1.14E-11 | 290 | 3.02E-13 | 720 | 1.21E-12 |
| 298 | 2.09E-11 | 730 | 1.14E-11 | 298 | 3.10E-13 | 730 | 1.24E-12 |
| 298.15 | 2.09E-11 | 740 | 1.15E-11 | 298.15 | 3.10E-13 | 740 | 1.28E-12 |
| 300 | 2.07E-11 | 750 | 1.16E-11 | 300 | 3.12E-13 | 750 | 1.32E-12 |
| 310 | 1.93E-11 | 760 | 1.17E-11 | 310 | 3.23E-13 | 760 | 1.35E-12 |
| 320 | 1.82E-11 | 770 | 1.18E-11 | 320 | 3.33E-13 | 770 | 1.39E-12 |
| 330 | 1.72E-11 | 780 | 1.19E-11 | 330 | 3.45E-13 | 780 | 1.43E-12 |
| 340 | 1.64E-11 | 790 | 1.20E-11 | 340 | 3.56E-13 | 790 | 1.47E-12 |
| 350 | 1.56E-11 | 800 | 1.21E-11 | 350 | 3.69E-13 | 800 | 1.51E-12 |
| 360 | 1.50E-11 | 900 | 1.33E-11 | 360 | 3.81E-13 | 900 | 1.98E-12 |
| 370 | 1.44E-11 | 1000 | 1.48E-11 | 370 | 3.94E-13 | 1000 | 2.53E-12 |
| 380 | 1.39E-11 | 1100 | 1.66E-11 | 380 | 4.08E-13 | 1100 | 3.20E-12 |
| 390 | 1.35E-11 | 1200 | 1.86E-11 | 390 | 4.22E-13 | 1200 | 3.97E-12 |
| 400 | 1.31E-11 | 1300 | 2.08E-11 | 400 | 4.36E-13 | 1300 | 4.87E-12 |
| 410 | 1.28E-11 | 1400 | 2.33E-11 | 410 | 4.51E-13 | 1400 | 5.90E-12 |
| 420 | 1.25E-11 | 1500 | 2.61E-11 | 420 | 4.67E-13 | 1500 | 7.06E-12 |
| 430 | 1.22E-11 | 1600 | 2.91E-11 | 430 | 4.83E-13 | 1600 | 8.37E-12 |
| 440 | 1.20E-11 | 1700 | 3.23E-11 | 440 | 4.99E-13 | 1700 | 9.84E-12 |
| 450 | 1.18E-11 | 1800 | 3.58E-11 | 450 | 5.16E-13 | 1800 | 1.15E-11 |
| 460 | 1.16E-11 | 1900 | 3.95E-11 | 460 | 5.34E-13 | 1900 | 1.32E-11 |
| 470 | 1.15E-11 | 2000 | 4.35E-11 | 470 | 5.52E-13 | 2000 | 1.52E-11 |
| 480 | 1.14E-11 | 2100 | 4.77E-11 | 480 | 5.71E-13 | 2100 | 1.73E-11 |
| 490 | 1.12E-11 | 2200 | 5.23E-11 | 490 | 5.90E-13 | 2200 | 1.97E-11 |
| 500 | 1.11E-11 | 2300 | 5.71E-11 | 500 | 6.10E-13 | 2300 | 2.22E-11 |
| 510 | 1.11E-11 | 2400 | 6.21E-11 | 510 | 6.30E-13 | 2400 | 2.49E-11 |
| 520 | 1.10E-11 | 2500 | 6.75E-11 | 520 | 6.51E-13 | 2500 | 2.78E-11 |
| 530 | 1.09E-11 | 2600 | 7.32E-11 | 530 | 6.73E-13 | 2600 | 3.09E-11 |
| 540 | 1.09E-11 | 2700 | 7.91E-11 | 540 | 6.95E-13 | 2700 | 3.42E-11 |
| 550 | 1.08E-11 | 2800 | 8.53E-11 | 550 | 7.18E-13 | 2800 | 3.78E-11 |
| 560 | 1.08E-11 | 2900 | 9.19E-11 | 560 | 7.41E-13 | 2900 | 4.15E-11 |
| 570 | 1.08E-11 | 3000 | 9.87E-11 | 570 | 7.65E-13 | 3000 | 4.55E-11 |

**Table S6**. High pressure limit rate constants (cm^3^ molecule^-1^ s^-1^) calculated by TST theory at the UCCSD(T)/aug-cc-pV(T+d)Z (energies) + UMP2/aug-cc-pVTZ (partition functions and ZPEs) level for the P1 and P2 adducts through the R1 and R2 pathways.

| T/K | P/bar | k (cm^3^molecule^−1^ s ^−1^) | T/K | P/bar | k (cm^3^molecule^−1^ s ^−1^) |
| --- | --- | --- | --- | --- | --- |
| 150 | 0.00$E$+00 | 4.45E-27 | 150 | 1.00$E$-06 | 2.16E-12 |
| 160 | 0.00$E$+00 |  | 160 | 1.00$E$-06 | 7.54E-13 |
| 170 | 0.00$E$+00 |  | 170 | 1.00$E$-06 | 3.00E-13 |
| 180 | 0.00$E$+00 |  | 180 | 1.00$E$-06 | 1.32E-13 |
| 190 | 0.00$E$+00 |  | 190 | 1.00$E$-06 | 6.40E-14 |
| 200 | 0.00$E$+00 |  | 200 | 1.00$E$-06 | 3.34E-14 |
| 210 | 0.00$E$+00 |  | 210 | 1.00$E$-06 | 1.86E-14 |
| 220 | 0.00$E$+00 |  | 220 | 1.00$E$-06 | 1.10E-14 |
| 230 | 0.00$E$+00 |  | 230 | 1.00$E$-06 | 6.80E-15 |
| 240 | 0.00$E$+00 |  | 240 | 1.00$E$-06 | 4.40E-15 |
| 250 | 0.00$E$+00 |  | 250 | 1.00$E$-06 | 2.96E-15 |
| 260 | 0.00$E$+00 |  | 260 | 1.00$E$-06 | 2.05E-15 |
| 270 | 0.00$E$+00 |  | 270 | 1.00$E$-06 | 1.47E-15 |
| 280 | 0.00$E$+00 |  | 280 | 1.00$E$-06 | 1.08E-15 |
| 290 | 0.00$E$+00 |  | 290 | 1.00$E$-06 | 8.15E-16 |
| 298 | 0.00$E$+00 | 4.44E-30 | 298 | 1.00$E$-06 | 6.60E-16 |
| 298.15 | 0.00$E$+00 |  | 298.15 | 1.00$E$-06 | 6.58E-16 |
| 300 | 0.00$E$+00 |  | 300 | 1.00$E$-06 | 6.28E-16 |
| 310 | 0.00$E$+00 |  | 310 | 1.00$E$-06 | 4.93E-16 |
| 320 | 0.00$E$+00 |  | 320 | 1.00$E$-06 | 3.94E-16 |
| 330 | 0.00$E$+00 |  | 330 | 1.00$E$-06 | 3.19E-16 |
| 340 | 0.00$E$+00 |  | 340 | 1.00$E$-06 | 2.63E-16 |
| 350 | 0.00$E$+00 |  | 350 | 1.00$E$-06 | 2.19E-16 |
| 360 | 0.00$E$+00 |  | 360 | 1.00$E$-06 | 1.85E-16 |
| 370 | 0.00$E$+00 |  | 370 | 1.00$E$-06 | 1.58E-16 |
| 380 | 0.00$E$+00 |  | 380 | 1.00$E$-06 | 1.37E-16 |
| 390 | 0.00$E$+00 |  | 390 | 1.00$E$-06 | 1.19E-16 |
| 400 | 0.00$E$+00 |  | 400 | 1.00$E$-06 | 1.04E-16 |
| 410 | 0.00$E$+00 |  | 410 | 1.00$E$-06 | 9.25E-17 |
| 420 | 0.00$E$+00 |  | 420 | 1.00$E$-06 | 8.25E-17 |
| 430 | 0.00$E$+00 |  | 430 | 1.00$E$-06 | 7.41E-17 |
| 440 | 0.00$E$+00 |  | 440 | 1.00$E$-06 | 6.70E-17 |
| 450 | 0.00$E$+00 |  | 450 | 1.00$E$-06 | 6.09E-17 |
| 460 | 0.00$E$+00 |  | 460 | 1.00$E$-06 | 5.57E-17 |
| 470 | 0.00$E$+00 |  | 470 | 1.00$E$-06 | 5.12E-17 |
| 480 | 0.00$E$+00 |  | 480 | 1.00$E$-06 | 4.73E-17 |
| 490 | 0.00$E$+00 |  | 490 | 1.00$E$-06 | 4.39E-17 |
| 500 | 0.00$E$+00 | 2.80E-31 | 500 | 1.00$E$-06 | 4.09E-17 |
| 510 | 0.00$E$+00 |  | 510 | 1.00$E$-06 | 3.82E-17 |
| 520 | 0.00$E$+00 |  | 520 | 1.00$E$-06 | 3.59E-17 |
| 530 | 0.00$E$+00 |  | 530 | 1.00$E$-06 | 3.38E-17 |
| 540 | 0.00$E$+00 |  | 540 | 1.00$E$-06 | 3.19E-17 |
| 550 | 0.00$E$+00 |  | 550 | 1.00$E$-06 | 3.03E-17 |
| 560 | 0.00$E$+00 |  | 560 | 1.00$E$-06 | 2.88E-17 |
| 570 | 0.00$E$+00 |  | 570 | 1.00$E$-06 | 2.75E-17 |
| 580 | 0.00$E$+00 |  | 580 | 1.00$E$-06 | 2.62E-17 |
| 590 | 0.00$E$+00 |  | 590 | 1.00$E$-06 | 2.51E-17 |
| 600 | 0.00$E$+00 |  | 600 | 1.00$E$-06 | 2.41E-17 |
| 610 | 0.00$E$+00 |  | 610 | 1.00$E$-06 | 2.32E-17 |
| 620 | 0.00$E$+00 |  | 620 | 1.00$E$-06 | 2.24E-17 |
| 630 | 0.00$E$+00 |  | 630 | 1.00$E$-06 | 2.17E-17 |
| 640 | 0.00$E$+00 |  | 640 | 1.00$E$-06 | 2.10E-17 |
| 650 | 0.00$E$+00 |  | 650 | 1.00$E$-06 | 2.03E-17 |
| 660 | 0.00$E$+00 |  | 660 | 1.00$E$-06 | 1.98E-17 |
| 670 | 0.00$E$+00 |  | 670 | 1.00$E$-06 | 1.92E-17 |
| 680 | 0.00$E$+00 |  | 680 | 1.00$E$-06 | 1.87E-17 |
| 690 | 0.00$E$+00 |  | 690 | 1.00$E$-06 | 1.83E-17 |
| 700 | 0.00$E$+00 | 1.71E-31 | 700 | 1.00$E$-06 | 1.79E-17 |
| 710 | 0.00$E$+00 |  | 710 | 1.00$E$-06 | 1.75E-17 |
| 720 | 0.00$E$+00 |  | 720 | 1.00$E$-06 | 1.71E-17 |
| 730 | 0.00$E$+00 |  | 730 | 1.00$E$-06 | 1.68E-17 |
| 740 | 0.00$E$+00 |  | 740 | 1.00$E$-06 | 1.65E-17 |
| 750 | 0.00$E$+00 |  | 750 | 1.00$E$-06 | 1.62E-17 |
| 760 | 0.00$E$+00 |  | 760 | 1.00$E$-06 | 1.60E-17 |
| 770 | 0.00$E$+00 |  | 770 | 1.00$E$-06 | 1.57E-17 |
| 780 | 0.00$E$+00 |  | 780 | 1.00$E$-06 | 1.55E-17 |
| 790 | 0.00$E$+00 |  | 790 | 1.00$E$-06 | 1.53E-17 |
| 800 | 0.00$E$+00 |  | 800 | 1.00$E$-06 | 1.51E-17 |
| T/K | P/bar | k (cm^3^molecule^−1^ s ^−1^) | T/K | P/bar | k (cm^3^molecule^−1^ s ^−1^) |
| 150 | 1.00$E$-05 | 2.17E-12 | 150 | 1.00$E$-04 | 2.25E-12 |
| 160 | 1.00E-05 | 7.58E-13 | 160 | 1.00$E$-04 | 7.92E-13 |
| 170 | 1.00E-05 | 3.00E-13 | 170 | 1.00$E$-04 | 3.05E-13 |
| 180 | 1.00E-05 | 1.33E-13 | 180 | 1.00$E$-04 | 1.35E-13 |
| 190 | 1.00E-05 | 6.41E-14 | 190 | 1.00$E$-04 | 6.54E-14 |
| 200 | 1.00E-05 | 3.35E-14 | 200 | 1.00$E$-04 | 3.42E-14 |
| 210 | 1.00E-05 | 1.86E-14 | 210 | 1.00$E$-04 | 1.91E-14 |
| 220 | 1.00E-05 | 1.10E-14 | 220 | 1.00$E$-04 | 1.13E-14 |
| 230 | 1.00E-05 | 6.82E-15 | 230 | 1.00$E$-04 | 7.00E-15 |
| 240 | 1.00E-05 | 4.41E-15 | 240 | 1.00$E$-04 | 4.54E-15 |
| 250 | 1.00E-05 | 2.96E-15 | 250 | 1.00$E$-04 | 3.06E-15 |
| 260 | 1.00E-05 | 2.06E-15 | 260 | 1.00$E$-04 | 2.13E-15 |
| 270 | 1.00E-05 | 1.48E-15 | 270 | 1.00$E$-04 | 1.53E-15 |
| 280 | 1.00E-05 | 1.09E-15 | 280 | 1.00$E$-04 | 1.12E-15 |
| 290 | 1.00E-05 | 8.18E-16 | 290 | 1.00$E$-04 | 8.48E-16 |
| 298 | 1.00E-05 | 6.63E-16 | 298 | 1.00$E$-04 | 6.88E-16 |
| 298.15 | 1.00E-05 | 6.60E-16 | 298.15 | 1.00$E$-04 | 6.85E-16 |
| 300 | 1.00E-05 | 6.30E-16 | 300 | 1.00$E$-04 | 6.54E-16 |
| 310 | 1.00E-05 | 4.95E-16 | 310 | 1.00$E$-04 | 5.14E-16 |
| 320 | 1.00E-05 | 3.95E-16 | 320 | 1.00$E$-04 | 4.11E-16 |
| 330 | 1.00E-05 | 3.21E-16 | 330 | 1.00$E$-04 | 3.34E-16 |
| 340 | 1.00E-05 | 2.64E-16 | 340 | 1.00$E$-04 | 2.74E-16 |
| 350 | 1.00E-05 | 2.20E-16 | 350 | 1.00$E$-04 | 2.29E-16 |
| 360 | 1.00E-05 | 1.86E-16 | 360 | 1.00$E$-04 | 1.94E-16 |
| 370 | 1.00E-05 | 1.59E-16 | 370 | 1.00$E$-04 | 1.66E-16 |
| 380 | 1.00E-05 | 1.37E-16 | 380 | 1.00$E$-04 | 1.43E-16 |
| 390 | 1.00E-05 | 1.19E-16 | 390 | 1.00$E$-04 | 1.25E-16 |
| 400 | 1.00E-05 | 1.05E-16 | 400 | 1.00$E$-04 | 1.10E-16 |
| 410 | 1.00E-05 | 9.30E-17 | 410 | 1.00$E$-04 | 9.76E-17 |
| 420 | 1.00E-05 | 8.29E-17 | 420 | 1.00$E$-04 | 8.72E-17 |
| 430 | 1.00E-05 | 7.45E-17 | 430 | 1.00$E$-04 | 7.84E-17 |
| 440 | 1.00E-05 | 6.74E-17 | 440 | 1.00$E$-04 | 7.10E-17 |
| 450 | 1.00E-05 | 6.13E-17 | 450 | 1.00$E$-04 | 6.47E-17 |
| 460 | 1.00E-05 | 5.60E-17 | 460 | 1.00$E$-04 | 5.92E-17 |
| 470 | 1.00E-05 | 5.15E-17 | 470 | 1.00$E$-04 | 5.45E-17 |
| 480 | 1.00E-05 | 4.76E-17 | 480 | 1.00$E$-04 | 5.04E-17 |
| 490 | 1.00E-05 | 4.41E-17 | 490 | 1.00$E$-04 | 4.68E-17 |
| 500 | 1.00E-05 | 4.10E-17 | 500 | 1.00$E$-04 | 4.28E-17 |
| 510 | 1.00E-05 | 3.84E-17 | 510 | 1.00$E$-04 | 4.01E-17 |
| 520 | 1.00E-05 | 3.60E-17 | 520 | 1.00$E$-04 | 3.77E-17 |
| 530 | 1.00E-05 | 3.40E-17 | 530 | 1.00$E$-04 | 3.55E-17 |
| 540 | 1.00E-05 | 3.21E-17 | 540 | 1.00$E$-04 | 3.36E-17 |
| 550 | 1.00E-05 | 3.04E-17 | 550 | 1.00$E$-04 | 3.19E-17 |
| 560 | 1.00E-05 | 2.89E-17 | 560 | 1.00$E$-04 | 3.04E-17 |
| 570 | 1.00E-05 | 2.76E-17 | 570 | 1.00$E$-04 | 2.90E-17 |
| 580 | 1.00E-05 | 2.64E-17 | 580 | 1.00$E$-04 | 2.77E-17 |
| 590 | 1.00E-05 | 2.53E-17 | 590 | 1.00$E$-04 | 2.66E-17 |
| 600 | 1.00E-05 | 2.43E-17 | 600 | 1.00$E$-04 | 2.56E-17 |
| 610 | 1.00E-05 | 2.34E-17 | 610 | 1.00$E$-04 | 2.47E-17 |
| 620 | 1.00E-05 | 2.25E-17 | 620 | 1.00$E$-04 | 2.38E-17 |
| 630 | 1.00E-05 | 2.18E-17 | 630 | 1.00$E$-04 | 2.30E-17 |
| 640 | 1.00E-05 | 2.11E-17 | 640 | 1.00$E$-04 | 2.23E-17 |
| 650 | 1.00E-05 | 2.05E-17 | 650 | 1.00$E$-04 | 2.17E-17 |
| 660 | 1.00E-05 | 1.99E-17 | 660 | 1.00$E$-04 | 2.11E-17 |
| 670 | 1.00E-05 | 1.93E-17 | 670 | 1.00$E$-04 | 2.02E-17 |
| 680 | 1.00E-05 | 1.88E-17 | 680 | 1.00$E$-04 | 1.97E-17 |
| 690 | 1.00E-05 | 1.84E-17 | 690 | 1.00$E$-04 | 1.93E-17 |
| 700 | 1.00E-05 | 1.80E-17 | 700 | 1.00$E$-04 | 1.88E-17 |
| 710 | 1.00E-05 | 1.76E-17 | 710 | 1.00$E$-04 | 1.85E-17 |
| 720 | 1.00E-05 | 1.72E-17 | 720 | 1.00$E$-04 | 1.81E-17 |
| 730 | 1.00E-05 | 1.69E-17 | 730 | 1.00$E$-04 | 1.78E-17 |
| 740 | 1.00E-05 | 1.66E-17 | 740 | 1.00$E$-04 | 1.75E-17 |
| 750 | 1.00E-05 | 1.63E-17 | 750 | 1.00$E$-04 | 1.72E-17 |
| 760 | 1.00E-05 | 1.60E-17 | 760 | 1.00$E$-04 | 1.69E-17 |
| 770 | 1.00E-05 | 1.58E-17 | 770 | 1.00$E$-04 | 1.67E-17 |
| 780 | 1.00E-05 | 1.56E-17 | 780 | 1.00$E$-04 | 1.65E-17 |
| 790 | 1.00E-05 | 1.54E-17 | 790 | 1.00$E$-04 | 1.63E-17 |
| 800 | 1.00E-05 | 1.52E-17 | 800 | 1.00$E$-04 | 1.61E-17 |
| T/K | P/bar | k (cm^3^molecule^−1^ s ^−1^) | T/K | P/bar | k (cm^3^molecule^−1^ s ^−1^) |
| 150 | 1.00$E$-03 | 3.12E-12 | 150 | 1.00$E$-02 | 1.03E-11 |
| 160 | 1.00$E$-03 | 1.13E-12 | 160 | 1.00$E$-02 | 3.94E-12 |
| 170 | 1.00$E$-03 | 3.56E-13 | 170 | 1.00$E$-02 | 8.30E-13 |
| 180 | 1.00$E$-03 | 1.60E-13 | 180 | 1.00$E$-02 | 3.92E-13 |
| 190 | 1.00$E$-03 | 7.87E-14 | 190 | 1.00$E$-02 | 2.02E-13 |
| 200 | 1.00$E$-03 | 4.17E-14 | 200 | 1.00$E$-02 | 1.11E-13 |
| 210 | 1.00$E$-03 | 2.36E-14 | 210 | 1.00$E$-02 | 6.55E-14 |
| 220 | 1.00$E$-03 | 1.41E-14 | 220 | 1.00$E$-02 | 4.06E-14 |
| 230 | 1.00$E$-03 | 8.87E-15 | 230 | 1.00$E$-02 | 2.64E-14 |
| 240 | 1.00$E$-03 | 5.82E-15 | 240 | 1.00$E$-02 | 1.78E-14 |
| 250 | 1.00$E$-03 | 3.96E-15 | 250 | 1.00$E$-02 | 1.25E-14 |
| 260 | 1.00$E$-03 | 2.79E-15 | 260 | 1.00$E$-02 | 8.99E-15 |
| 270 | 1.00$E$-03 | 2.02E-15 | 270 | 1.00$E$-02 | 6.67E-15 |
| 280 | 1.00$E$-03 | 1.50E-15 | 280 | 1.00$E$-02 | 5.07E-15 |
| 290 | 1.00$E$-03 | 1.14E-15 | 290 | 1.00$E$-02 | 3.93E-15 |
| 298 | 1.00$E$-03 | 9.33E-16 | 298 | 1.00$E$-02 | 3.26E-15 |
| 298.15 | 1.00$E$-03 | 9.30E-16 | 298.15 | 1.00$E$-02 | 3.25E-15 |
| 300 | 1.00$E$-03 | 8.89E-16 | 300 | 1.00$E$-02 | 3.12E-15 |
| 310 | 1.00$E$-03 | 7.04E-16 | 310 | 1.00$E$-02 | 2.51E-15 |
| 320 | 1.00$E$-03 | 5.68E-16 | 320 | 1.00$E$-02 | 2.06E-15 |
| 330 | 1.00$E$-03 | 4.65E-16 | 330 | 1.00$E$-02 | 1.71E-15 |
| 340 | 1.00$E$-03 | 3.78E-16 | 340 | 1.00$E$-02 | 1.38E-15 |
| 350 | 1.00$E$-03 | 3.19E-16 | 350 | 1.00$E$-02 | 1.19E-15 |
| 360 | 1.00$E$-03 | 2.72E-16 | 360 | 1.00$E$-02 | 1.03E-15 |
| 370 | 1.00$E$-03 | 2.35E-16 | 370 | 1.00$E$-02 | 9.10E-16 |
| 380 | 1.00$E$-03 | 2.05E-16 | 380 | 1.00$E$-02 | 8.08E-16 |
| 390 | 1.00$E$-03 | 1.81E-16 | 390 | 1.00$E$-02 | 7.23E-16 |
| 400 | 1.00$E$-03 | 1.60E-16 | 400 | 1.00$E$-02 | 6.53E-16 |
| 410 | 1.00$E$-03 | 1.44E-16 | 410 | 1.00$E$-02 | 5.93E-16 |
| 420 | 1.00$E$-03 | 1.29E-16 | 420 | 1.00$E$-02 | 5.43E-16 |
| 430 | 1.00$E$-03 | 1.17E-16 | 430 | 1.00$E$-02 | 4.99E-16 |
| 440 | 1.00$E$-03 | 1.07E-16 | 440 | 1.00$E$-02 | 4.62E-16 |
| 450 | 1.00$E$-03 | 9.85E-17 | 450 | 1.00$E$-02 | 4.30E-16 |
| 460 | 1.00$E$-03 | 9.10E-17 | 460 | 1.00$E$-02 | 4.02E-16 |
| 470 | 1.00$E$-03 | 8.44E-17 | 470 | 1.00$E$-02 | 3.78E-16 |
| 480 | 1.00$E$-03 | 7.87E-17 | 480 | 1.00$E$-02 | 3.56E-16 |
| 490 | 1.00$E$-03 | 7.37E-17 | 490 | 1.00$E$-02 | 3.38E-16 |
| 500 | 1.00$E$-03 | 6.02E-17 | 500 | 1.00$E$-02 | 2.33E-16 |
| 510 | 1.00$E$-03 | 5.68E-17 | 510 | 1.00$E$-02 | 2.22E-16 |
| 520 | 1.00$E$-03 | 5.38E-17 | 520 | 1.00$E$-02 | 2.13E-16 |
| 530 | 1.00$E$-03 | 5.11E-17 | 530 | 1.00$E$-02 | 2.05E-16 |
| 540 | 1.00$E$-03 | 4.87E-17 | 540 | 1.00$E$-02 | 1.98E-16 |
| 550 | 1.00$E$-03 | 4.66E-17 | 550 | 1.00$E$-02 | 1.92E-16 |
| 560 | 1.00$E$-03 | 4.46E-17 | 560 | 1.00$E$-02 | 1.86E-16 |
| 570 | 1.00$E$-03 | 4.29E-17 | 570 | 1.00$E$-02 | 1.81E-16 |
| 580 | 1.00$E$-03 | 4.14E-17 | 580 | 1.00$E$-02 | 1.77E-16 |
| 590 | 1.00$E$-03 | 4.00E-17 | 590 | 1.00$E$-02 | 1.72E-16 |
| 600 | 1.00$E$-03 | 3.87E-17 | 600 | 1.00$E$-02 | 1.69E-16 |
| 610 | 1.00$E$-03 | 3.75E-17 | 610 | 1.00$E$-02 | 1.66E-16 |
| 620 | 1.00$E$-03 | 3.65E-17 | 620 | 1.00$E$-02 | 1.63E-16 |
| 630 | 1.00$E$-03 | 3.56E-17 | 630 | 1.00$E$-02 | 1.60E-16 |
| 640 | 1.00$E$-03 | 3.47E-17 | 640 | 1.00$E$-02 | 1.58E-16 |
| 650 | 1.00$E$-03 | 3.39E-17 | 650 | 1.00$E$-02 | 1.56E-16 |
| 660 | 1.00$E$-03 | 3.32E-17 | 660 | 1.00$E$-02 | 1.54E-16 |
| 670 | 1.00$E$-03 | 2.91E-17 | 670 | 1.00$E$-02 | 1.18E-16 |
| 680 | 1.00$E$-03 | 2.86E-17 | 680 | 1.00$E$-02 | 1.17E-16 |
| 690 | 1.00$E$-03 | 2.81E-17 | 690 | 1.00$E$-02 | 1.16E-16 |
| 700 | 1.00$E$-03 | 2.77E-17 | 700 | 1.00$E$-02 | 1.16E-16 |
| 710 | 1.00$E$-03 | 2.73E-17 | 710 | 1.00$E$-02 | 1.15E-16 |
| 720 | 1.00$E$-03 | 2.69E-17 | 720 | 1.00$E$-02 | 1.15E-16 |
| 730 | 1.00$E$-03 | 2.66E-17 | 730 | 1.00$E$-02 | 1.15E-16 |
| 740 | 1.00$E$-03 | 2.63E-17 | 740 | 1.00$E$-02 | 1.15E-16 |
| 750 | 1.00$E$-03 | 2.61E-17 | 750 | 1.00$E$-02 | 1.15E-16 |
| 760 | 1.00$E$-03 | 2.59E-17 | 760 | 1.00$E$-02 | 1.15E-16 |
| 770 | 1.00$E$-03 | 2.57E-17 | 770 | 1.00$E$-02 | 1.15E-16 |
| 780 | 1.00$E$-03 | 2.55E-17 | 780 | 1.00$E$-02 | 1.15E-16 |
| 790 | 1.00$E$-03 | 2.54E-17 | 790 | 1.00$E$-02 | 1.16E-16 |
| 800 | 1.00$E$-03 | 2.52E-17 | 800 | 1.00$E$-02 | 1.16E-16 |
| T/K | P/bar | k (cm^3^molecule^−1^ s ^−1^) | T/K | P/bar | k (cm^3^molecule^−1^ s ^−1^) |
| 150 | 1.00$E$-01 | 4.62E-11 | 150 | 1.00$E$-00 | 1.49E-10 |
| 160 | 1.00$E$-01 | 1.83E-11 | 160 | 1.00$E$-00 | 6.16E-11 |
| 170 | 1.00$E$-01 | 4.02E-12 | 170 | 1.00$E$-00 | 1.99E-11 |
| 180 | 1.00$E$-01 | 1.97E-12 | 180 | 1.00$E$-00 | 1.01E-11 |
| 190 | 1.00$E$-01 | 1.05E-12 | 190 | 1.00$E$-00 | 5.52E-12 |
| 200 | 1.00$E$-01 | 5.96E-13 | 200 | 1.00$E$-00 | 3.23E-12 |
| 210 | 1.00$E$-01 | 3.60E-13 | 210 | 1.00$E$-00 | 2.00E-12 |
| 220 | 1.00$E$-01 | 2.28E-13 | 220 | 1.00$E$-00 | 1.30E-12 |
| 230 | 1.00$E$-01 | 1.51E-13 | 230 | 1.00$E$-00 | 8.82E-13 |
| 240 | 1.00$E$-01 | 1.04E-13 | 240 | 1.00$E$-00 | 6.20E-13 |
| 250 | 1.00$E$-01 | 7.43E-14 | 250 | 1.00$E$-00 | 4.51E-13 |
| 260 | 1.00$E$-01 | 5.45E-14 | 260 | 1.00$E$-00 | 3.37E-13 |
| 270 | 1.00$E$-01 | 4.10E-14 | 270 | 1.00$E$-00 | 2.59E-13 |
| 280 | 1.00$E$-01 | 3.16E-14 | 280 | 1.00$E$-00 | 2.03E-13 |
| 290 | 1.00$E$-01 | 2.49E-14 | 290 | 1.00$E$-00 | 1.62E-13 |
| 298 | 1.00$E$-01 | 2.09E-14 | 298 | 1.00$E$-00 | 1.38E-13 |
| 298.15 | 1.00$E$-01 | 2.08E-14 | 298.15 | 1.00$E$-00 | 1.37E-13 |
| 300 | 1.00$E$-01 | 2.00E-14 | 300 | 1.00$E$-00 | 1.32E-13 |
| 310 | 1.00$E$-01 | 1.63E-14 | 310 | 1.00$E$-00 | 1.10E-13 |
| 320 | 1.00$E$-01 | 1.35E-14 | 320 | 1.00$E$-00 | 9.21E-14 |
| 330 | 1.00$E$-01 | 1.13E-14 | 330 | 1.00$E$-00 | 7.85E-14 |
| 340 | 1.00$E$-01 | 9.35E-15 | 340 | 1.00$E$-00 | 5.43E-14 |
| 350 | 1.00$E$-01 | 8.15E-15 | 350 | 1.00$E$-00 | 4.78E-14 |
| 360 | 1.00$E$-01 | 7.18E-15 | 360 | 1.00$E$-00 | 4.26E-14 |
| 370 | 1.00$E$-01 | 6.39E-15 | 370 | 1.00$E$-00 | 3.82E-14 |
| 380 | 1.00$E$-01 | 5.73E-15 | 380 | 1.00$E$-00 | 3.46E-14 |
| 390 | 1.00$E$-01 | 5.18E-15 | 390 | 1.00$E$-00 | 3.16E-14 |
| 400 | 1.00$E$-01 | 4.72E-15 | 400 | 1.00$E$-00 | 2.90E-14 |
| 410 | 1.00$E$-01 | 4.33E-15 | 410 | 1.00$E$-00 | 2.68E-14 |
| 420 | 1.00$E$-01 | 4.00E-15 | 420 | 1.00$E$-00 | 2.49E-14 |
| 430 | 1.00$E$-01 | 3.71E-15 | 430 | 1.00$E$-00 | 2.33E-14 |
| 440 | 1.00$E$-01 | 3.46E-15 | 440 | 1.00$E$-00 | 2.18E-14 |
| 450 | 1.00$E$-01 | 3.25E-15 | 450 | 1.00$E$-00 | 2.06E-14 |
| 460 | 1.00$E$-01 | 3.06E-15 | 460 | 1.00$E$-00 | 1.95E-14 |
| 470 | 1.00$E$-01 | 2.90E-15 | 470 | 1.00$E$-00 | 1.86E-14 |
| 480 | 1.00$E$-01 | 2.75E-15 | 480 | 1.00$E$-00 | 1.77E-14 |
| 490 | 1.00$E$-01 | 2.62E-15 | 490 | 1.00$E$-00 | 1.70E-14 |
| 500 | 1.00$E$-01 | 1.81E-15 | 500 | 1.00$E$-00 | 1.33E-14 |
| 510 | 1.00$E$-01 | 1.74E-15 | 510 | 1.00$E$-00 | 1.29E-14 |
| 520 | 1.00$E$-01 | 1.69E-15 | 520 | 1.00$E$-00 | 1.26E-14 |
| 530 | 1.00$E$-01 | 1.63E-15 | 530 | 1.00$E$-00 | 1.22E-14 |
| 540 | 1.00$E$-01 | 1.59E-15 | 540 | 1.00$E$-00 | 1.20E-14 |
| 550 | 1.00$E$-01 | 1.54E-15 | 550 | 1.00$E$-00 | 1.17E-14 |
| 560 | 1.00$E$-01 | 1.51E-15 | 560 | 1.00$E$-00 | 1.15E-14 |
| 570 | 1.00$E$-01 | 1.47E-15 | 570 | 1.00$E$-00 | 1.13E-14 |
| 580 | 1.00$E$-01 | 1.44E-15 | 580 | 1.00$E$-00 | 1.11E-14 |
| 590 | 1.00$E$-01 | 1.42E-15 | 590 | 1.00$E$-00 | 1.10E-14 |
| 600 | 1.00$E$-01 | 1.39E-15 | 600 | 1.00$E$-00 | 1.09E-14 |
| 610 | 1.00$E$-01 | 1.37E-15 | 610 | 1.00$E$-00 | 1.08E-14 |
| 620 | 1.00$E$-01 | 1.36E-15 | 620 | 1.00$E$-00 | 1.07E-14 |
| 630 | 1.00$E$-01 | 1.34E-15 | 630 | 1.00$E$-00 | 1.06E-14 |
| 640 | 1.00$E$-01 | 1.33E-15 | 640 | 1.00$E$-00 | 1.05E-14 |
| 650 | 1.00$E$-01 | 1.32E-15 | 650 | 1.00$E$-00 | 1.05E-14 |
| 660 | 1.00$E$-01 | 1.31E-15 | 660 | 1.00$E$-00 | 1.04E-14 |
| 670 | 1.00$E$-01 | 9.77E-16 | 670 | 1.00$E$-00 | 8.16E-15 |
| 680 | 1.00$E$-01 | 9.74E-16 | 680 | 1.00$E$-00 | 8.17E-15 |
| 690 | 1.00$E$-01 | 9.72E-16 | 690 | 1.00$E$-00 | 8.18E-15 |
| 700 | 1.00$E$-01 | 9.72E-16 | 700 | 1.00$E$-00 | 8.20E-15 |
| 710 | 1.00$E$-01 | 9.72E-16 | 710 | 1.00$E$-00 | 8.23E-15 |
| 720 | 1.00$E$-01 | 9.73E-16 | 720 | 1.00$E$-00 | 8.26E-15 |
| 730 | 1.00$E$-01 | 9.75E-16 | 730 | 1.00$E$-00 | 8.30E-15 |
| 740 | 1.00$E$-01 | 9.78E-16 | 740 | 1.00$E$-00 | 8.35E-15 |
| 750 | 1.00$E$-01 | 9.82E-16 | 750 | 1.00$E$-00 | 8.40E-15 |
| 760 | 1.00$E$-01 | 9.86E-16 | 760 | 1.00$E$-00 | 8.46E-15 |
| 770 | 1.00$E$-01 | 9.91E-16 | 770 | 1.00$E$-00 | 8.53E-15 |
| 780 | 1.00$E$-01 | 9.97E-16 | 780 | 1.00$E$-00 | 8.60E-15 |
| 790 | 1.00$E$-01 | 1.00E-15 | 790 | 1.00$E$-00 | 8.67E-15 |
| 800 | 1.00$E$-01 | 1.01E-15 | 800 | 1.00$E$-00 | 8.75E-15 |
| T/K | P/bar | k (cm^3^molecule^−1^ s ^−1^) | T/K | P/bar | k (cm^3^molecule^−1^ s ^−1^) |
| 150 | 1.00$E$+01 | 3.83E-10 | 150 | 1.00$E$+02 | 7.56E-10 |
| 160 | 1.00$E$+01 | 1.68E-10 | 160 | 1.00$E$+02 | 3.56E-10 |
| 170 | 1.00$E$+01 | 7.38E-11 | 170 | 1.00$E$+02 | 1.82E-10 |
| 180 | 1.00$E$+01 | 3.92E-11 | 180 | 1.00$E$+02 | 1.03E-10 |
| 190 | 1.00$E$+01 | 2.24E-11 | 190 | 1.00$E$+02 | 6.28E-11 |
| 200 | 1.00$E$+01 | 1.37E-11 | 200 | 1.00$E$+02 | 4.04E-11 |
| 210 | 1.00$E$+01 | 8.80E-12 | 210 | 1.00$E$+02 | 2.73E-11 |
| 220 | 1.00$E$+01 | 5.93E-12 | 220 | 1.00$E$+02 | 1.93E-11 |
| 230 | 1.00$E$+01 | 4.15E-12 | 230 | 1.00$E$+02 | 1.41E-11 |
| 240 | 1.00$E$+01 | 3.02E-12 | 240 | 1.00$E$+02 | 1.06E-11 |
| 250 | 1.00$E$+01 | 2.26E-12 | 250 | 1.00$E$+02 | 8.25E-12 |
| 260 | 1.00$E$+01 | 1.73E-12 | 260 | 1.00$E$+02 | 6.56E-12 |
| 270 | 1.00$E$+01 | 1.37E-12 | 270 | 1.00$E$+02 | 5.33E-12 |
| 280 | 1.00$E$+01 | 1.10E-12 | 280 | 1.00$E$+02 | 4.41E-12 |
| 290 | 1.00$E$+01 | 9.00E-13 | 290 | 1.00$E$+02 | 3.71E-12 |
| 298 | 1.00$E$+01 | 7.77E-13 | 298 | 1.00$E$+02 | 3.27E-12 |
| 298.15 | 1.00$E$+01 | 7.75E-13 | 298.15 | 1.00$E$+02 | 3.26E-12 |
| 300 | 1.00$E$+01 | 7.50E-13 | 300 | 1.00$E$+02 | 3.17E-12 |
| 310 | 1.00$E$+01 | 6.35E-13 | 310 | 1.00$E$+02 | 2.75E-12 |
| 320 | 1.00$E$+01 | 5.45E-13 | 320 | 1.00$E$+02 | 2.41E-12 |
| 330 | 1.00$E$+01 | 4.74E-13 | 330 | 1.00$E$+02 | 2.14E-12 |
| 340 | 1.00$E$+01 | 2.67E-13 | 340 | 1.00$E$+02 | 1.37E-12 |
| 350 | 1.00$E$+01 | 2.37E-13 | 350 | 1.00$E$+02 | 1.23E-12 |
| 360 | 1.00$E$+01 | 2.13E-13 | 360 | 1.00$E$+02 | 1.12E-12 |
| 370 | 1.00$E$+01 | 1.93E-13 | 370 | 1.00$E$+02 | 1.03E-12 |
| 380 | 1.00$E$+01 | 1.75E-13 | 380 | 1.00$E$+02 | 9.49E-13 |
| 390 | 1.00$E$+01 | 1.61E-13 | 390 | 1.00$E$+02 | 8.82E-13 |
| 400 | 1.00$E$+01 | 1.49E-13 | 400 | 1.00$E$+02 | 8.24E-13 |
| 410 | 1.00$E$+01 | 1.38E-13 | 410 | 1.00$E$+02 | 7.74E-13 |
| 420 | 1.00$E$+01 | 1.29E-13 | 420 | 1.00$E$+02 | 7.31E-13 |
| 430 | 1.00$E$+01 | 1.21E-13 | 430 | 1.00$E$+02 | 6.94E-13 |
| 440 | 1.00$E$+01 | 1.14E-13 | 440 | 1.00$E$+02 | 6.61E-13 |
| 450 | 1.00$E$+01 | 1.08E-13 | 450 | 1.00$E$+02 | 6.32E-13 |
| 460 | 1.00$E$+01 | 1.03E-13 | 460 | 1.00$E$+02 | 6.07E-13 |
| 470 | 1.00$E$+01 | 9.79E-14 | 470 | 1.00$E$+02 | 5.85E-13 |
| 480 | 1.00$E$+01 | 9.38E-14 | 480 | 1.00$E$+02 | 5.65E-13 |
| 490 | 1.00$E$+01 | 9.01E-14 | 490 | 1.00$E$+02 | 5.48E-13 |
| 500 | 1.00$E$+01 | 8.57E-14 | 500 | 1.00$E$+02 | 4.81E-13 |
| 510 | 1.00$E$+01 | 8.36E-14 | 510 | 1.00$E$+02 | 4.71E-13 |
| 520 | 1.00$E$+01 | 8.18E-14 | 520 | 1.00$E$+02 | 4.63E-13 |
| 530 | 1.00$E$+01 | 8.02E-14 | 530 | 1.00$E$+02 | 4.55E-13 |
| 540 | 1.00$E$+01 | 7.88E-14 | 540 | 1.00$E$+02 | 4.49E-13 |
| 550 | 1.00$E$+01 | 7.76E-14 | 550 | 1.00$E$+02 | 4.43E-13 |
| 560 | 1.00$E$+01 | 7.65E-14 | 560 | 1.00$E$+02 | 4.38E-13 |
| 570 | 1.00$E$+01 | 7.56E-14 | 570 | 1.00$E$+02 | 4.34E-13 |
| 580 | 1.00$E$+01 | 7.49E-14 | 580 | 1.00$E$+02 | 4.31E-13 |
| 590 | 1.00$E$+01 | 7.43E-14 | 590 | 1.00$E$+02 | 4.28E-13 |
| 600 | 1.00$E$+01 | 7.37E-14 | 600 | 1.00$E$+02 | 4.26E-13 |
| 610 | 1.00$E$+01 | 7.33E-14 | 610 | 1.00$E$+02 | 4.24E-13 |
| 620 | 1.00$E$+01 | 7.30E-14 | 620 | 1.00$E$+02 | 4.23E-13 |
| 630 | 1.00$E$+01 | 7.28E-14 | 630 | 1.00$E$+02 | 4.22E-13 |
| 640 | 1.00$E$+01 | 7.26E-14 | 640 | 1.00$E$+02 | 4.21E-13 |
| 650 | 1.00$E$+01 | 7.26E-14 | 650 | 1.00$E$+02 | 4.21E-13 |
| 660 | 1.00$E$+01 | 7.26E-14 | 660 | 1.00$E$+02 | 4.21E-13 |
| 670 | 1.00$E$+01 | 6.07E-14 | 670 | 1.00$E$+02 | 4.02E-13 |
| 680 | 1.00$E$+01 | 6.09E-14 | 680 | 1.00$E$+02 | 4.05E-13 |
| 690 | 1.00$E$+01 | 6.12E-14 | 690 | 1.00$E$+02 | 4.09E-13 |
| 700 | 1.00$E$+01 | 6.16E-14 | 700 | 1.00$E$+02 | 4.13E-13 |
| 710 | 1.00$E$+01 | 6.20E-14 | 710 | 1.00$E$+02 | 4.17E-13 |
| 720 | 1.00$E$+01 | 6.25E-14 | 720 | 1.00$E$+02 | 4.21E-13 |
| 730 | 1.00$E$+01 | 6.30E-14 | 730 | 1.00$E$+02 | 4.26E-13 |
| 740 | 1.00$E$+01 | 6.36E-14 | 740 | 1.00$E$+02 | 4.31E-13 |
| 750 | 1.00$E$+01 | 6.42E-14 | 750 | 1.00$E$+02 | 4.36E-13 |
| 760 | 1.00$E$+01 | 6.48E-14 | 760 | 1.00$E$+02 | 4.42E-13 |
| 770 | 1.00$E$+01 | 6.55E-14 | 770 | 1.00$E$+02 | 4.48E-13 |
| 780 | 1.00$E$+01 | 6.62E-14 | 780 | 1.00$E$+02 | 4.54E-13 |
| 790 | 1.00$E$+01 | 6.69E-14 | 790 | 1.00$E$+02 | 4.60E-13 |
| 800 | 1.00$E$+01 | 6.77E-14 | 800 | 1.00$E$+02 | 4.66E-13 |
| T/K | P/bar | k (cm^3^molecule^−1^ s ^−1^) | T/K | P/bar | k (cm^3^molecule^−1^ s ^−1^) |
| 150 | 1.00$E$+03 | 1.09E-09 | 150 | 1.00$E$+04 | 1.23E-09 |
| 160 | 1.00$E$+03 | 5.54E-10 | 160 | 1.00$E$+04 | 6.42E-10 |
| 170 | 1.00$E$+03 | 3.09E-10 | 170 | 1.00$E$+04 | 3.74E-10 |
| 180 | 1.00$E$+03 | 1.88E-10 | 180 | 1.00$E$+04 | 2.36E-10 |
| 190 | 1.00$E$+03 | 1.22E-10 | 190 | 1.00$E$+04 | 1.60E-10 |
| 200 | 1.00$E$+03 | 8.36E-11 | 200 | 1.00$E$+04 | 1.15E-10 |
| 210 | 1.00$E$+03 | 5.99E-11 | 210 | 1.00$E$+04 | 8.59E-11 |
| 220 | 1.00$E$+03 | 4.47E-11 | 220 | 1.00$E$+04 | 6.68E-11 |
| 230 | 1.00$E$+03 | 3.44E-11 | 230 | 1.00$E$+04 | 5.36E-11 |
| 240 | 1.00$E$+03 | 2.72E-11 | 240 | 1.00$E$+04 | 4.42E-11 |
| 250 | 1.00$E$+03 | 2.20E-11 | 250 | 1.00$E$+04 | 3.72E-11 |
| 260 | 1.00$E$+03 | 1.82E-11 | 260 | 1.00$E$+04 | 3.20E-11 |
| 270 | 1.00$E$+03 | 1.53E-11 | 270 | 1.00$E$+04 | 2.79E-11 |
| 280 | 1.00$E$+03 | 1.31E-11 | 280 | 1.00$E$+04 | 2.48E-11 |
| 290 | 1.00$E$+03 | 1.14E-11 | 290 | 1.00$E$+04 | 2.22E-11 |
| 298 | 1.00$E$+03 | 1.03E-11 | 298 | 1.00$E$+04 | 2.05E-11 |
| 298.15 | 1.00$E$+03 | 1.03E-11 | 298.15 | 1.00$E$+04 | 2.05E-11 |
| 300 | 1.00$E$+03 | 1.00E-11 | 300 | 1.00$E$+04 | 2.02E-11 |
| 310 | 1.00$E$+03 | 8.94E-12 | 310 | 1.00$E$+04 | 1.85E-11 |
| 320 | 1.00$E$+03 | 8.04E-12 | 320 | 1.00$E$+04 | 1.71E-11 |
| 330 | 1.00$E$+03 | 7.30E-12 | 330 | 1.00$E$+04 | 1.59E-11 |
| 340 | 1.00$E$+03 | 5.95E-12 | 340 | 1.00$E$+04 | 1.46E-11 |
| 350 | 1.00$E$+03 | 5.49E-12 | 350 | 1.00$E$+04 | 1.38E-11 |
| 360 | 1.00$E$+03 | 5.09E-12 | 360 | 1.00$E$+04 | 1.31E-11 |
| 370 | 1.00$E$+03 | 4.76E-12 | 370 | 1.00$E$+04 | 1.25E-11 |
| 380 | 1.00$E$+03 | 4.47E-12 | 380 | 1.00$E$+04 | 1.20E-11 |
| 390 | 1.00$E$+03 | 4.23E-12 | 390 | 1.00$E$+04 | 1.15E-11 |
| 400 | 1.00$E$+03 | 4.02E-12 | 400 | 1.00$E$+04 | 1.12E-11 |
| 410 | 1.00$E$+03 | 3.83E-12 | 410 | 1.00$E$+04 | 1.08E-11 |
| 420 | 1.00$E$+03 | 3.67E-12 | 420 | 1.00$E$+04 | 1.05E-11 |
| 430 | 1.00$E$+03 | 3.54E-12 | 430 | 1.00$E$+04 | 1.03E-11 |
| 440 | 1.00$E$+03 | 3.42E-12 | 440 | 1.00$E$+04 | 1.01E-11 |
| 450 | 1.00$E$+03 | 3.31E-12 | 450 | 1.00$E$+04 | 9.89E-12 |
| 460 | 1.00$E$+03 | 3.22E-12 | 460 | 1.00$E$+04 | 9.74E-12 |
| 470 | 1.00$E$+03 | 3.14E-12 | 470 | 1.00$E$+04 | 9.61E-12 |
| 480 | 1.00$E$+03 | 3.07E-12 | 480 | 1.00$E$+04 | 9.50E-12 |
| 490 | 1.00$E$+03 | 3.01E-12 | 490 | 1.00$E$+04 | 9.41E-12 |
| 500 | 1.00$E$+03 | 2.25E-12 | 500 | 1.00$E$+04 | 8.42E-12 |
| 510 | 1.00$E$+03 | 2.21E-12 | 510 | 1.00$E$+04 | 8.38E-12 |
| 520 | 1.00$E$+03 | 2.18E-12 | 520 | 1.00$E$+04 | 8.34E-12 |
| 530 | 1.00$E$+03 | 2.16E-12 | 530 | 1.00$E$+04 | 8.32E-12 |
| 540 | 1.00$E$+03 | 2.13E-12 | 540 | 1.00$E$+04 | 8.32E-12 |
| 550 | 1.00$E$+03 | 2.11E-12 | 550 | 1.00$E$+04 | 8.32E-12 |
| 560 | 1.00$E$+03 | 2.10E-12 | 560 | 1.00$E$+04 | 8.33E-12 |
| 570 | 1.00$E$+03 | 2.09E-12 | 570 | 1.00$E$+04 | 8.35E-12 |
| 580 | 1.00$E$+03 | 2.08E-12 | 580 | 1.00$E$+04 | 8.39E-12 |
| 590 | 1.00$E$+03 | 2.07E-12 | 590 | 1.00$E$+04 | 8.43E-12 |
| 600 | 1.00$E$+03 | 2.06E-12 | 600 | 1.00$E$+04 | 8.48E-12 |
| 610 | 1.00$E$+03 | 2.06E-12 | 610 | 1.00$E$+04 | 8.53E-12 |
| 620 | 1.00$E$+03 | 2.06E-12 | 620 | 1.00$E$+04 | 8.60E-12 |
| 630 | 1.00$E$+03 | 2.06E-12 | 630 | 1.00$E$+04 | 8.67E-12 |
| 640 | 1.00$E$+03 | 2.07E-12 | 640 | 1.00$E$+04 | 8.75E-12 |
| 650 | 1.00$E$+03 | 2.07E-12 | 650 | 1.00$E$+04 | 8.83E-12 |
| 660 | 1.00$E$+03 | 2.08E-12 | 660 | 1.00$E$+04 | 8.93E-12 |
| 670 | 1.00$E$+03 | 2.15E-12 | 670 | 1.00$E$+04 | 7.98E-12 |
| 680 | 1.00$E$+03 | 2.17E-12 | 680 | 1.00$E$+04 | 8.07E-12 |
| 690 | 1.00$E$+03 | 2.19E-12 | 690 | 1.00$E$+04 | 8.16E-12 |
| 700 | 1.00$E$+03 | 2.21E-12 | 700 | 1.00$E$+04 | 8.26E-12 |
| 710 | 1.00$E$+03 | 2.23E-12 | 710 | 1.00$E$+04 | 8.36E-12 |
| 720 | 1.00$E$+03 | 2.26E-12 | 720 | 1.00$E$+04 | 8.46E-12 |
| 730 | 1.00$E$+03 | 2.28E-12 | 730 | 1.00$E$+04 | 8.58E-12 |
| 740 | 1.00$E$+03 | 2.31E-12 | 740 | 1.00$E$+04 | 8.69E-12 |
| 750 | 1.00$E$+03 | 2.33E-12 | 750 | 1.00$E$+04 | 8.82E-12 |
| 760 | 1.00$E$+03 | 2.36E-12 | 760 | 1.00$E$+04 | 8.94E-12 |
| 770 | 1.00$E$+03 | 2.39E-12 | 770 | 1.00$E$+04 | 9.07E-12 |
| 780 | 1.00$E$+03 | 2.42E-12 | 780 | 1.00$E$+04 | 9.21E-12 |
| 790 | 1.00$E$+03 | 2.45E-12 | 790 | 1.00$E$+04 | 9.35E-12 |
| 800 | 1.00$E$+03 | 2.48E-12 | 800 | 1.00$E$+04 | 9.50E-12 |
| T/K | P/bar | k (cm^3^molecule^−1^ s ^−1^) | T/K | P/bar | k (cm^3^molecule^−1^ s ^−1^) |
| 150 | 1.00$E$+05 | 1.25E-09 | 150 | 1.00E+06 | 1.25E-09 |
| 160 | 1.00$E$+05 | 6.58E-10 | 160 | 1.00E+06 | 6.60E-10 |
| 170 | 1.00$E$+05 | 3.86E-10 | 170 | 1.00E+06 | 3.87E-10 |
| 180 | 1.00$E$+05 | 2.47E-10 | 180 | 1.00E+06 | 2.48E-10 |
| 190 | 1.00$E$+05 | 1.69E-10 | 190 | 1.00E+06 | 1.70E-10 |
| 200 | 1.00$E$+05 | 1.22E-10 | 200 | 1.00E+06 | 1.23E-10 |
| 210 | 1.00$E$+05 | 9.28E-11 | 210 | 1.00E+06 | 9.37E-11 |
| 220 | 1.00$E$+05 | 7.32E-11 | 220 | 1.00E+06 | 7.40E-11 |
| 230 | 1.00$E$+05 | 5.96E-11 | 230 | 1.00E+06 | 6.04E-11 |
| 240 | 1.00$E$+05 | 4.99E-11 | 240 | 1.00E+06 | 5.07E-11 |
| 250 | 1.00$E$+05 | 4.27E-11 | 250 | 1.00E+06 | 4.35E-11 |
| 260 | 1.00$E$+05 | 3.73E-11 | 260 | 1.00E+06 | 3.81E-11 |
| 270 | 1.00$E$+05 | 3.31E-11 | 270 | 1.00E+06 | 3.39E-11 |
| 280 | 1.00$E$+05 | 2.98E-11 | 280 | 1.00E+06 | 3.06E-11 |
| 290 | 1.00$E$+05 | 2.72E-11 | 290 | 1.00E+06 | 2.80E-11 |
| 298 | 1.00$E$+05 | 2.55E-11 | 298 | 1.00E+06 | 2.63E-11 |
| 298.15 | 1.00$E$+05 | 2.55E-11 | 298.15 | 1.00E+06 | 2.63E-11 |
| 300 | 1.00$E$+05 | 2.51E-11 | 300 | 1.00E+06 | 2.60E-11 |
| 310 | 1.00$E$+05 | 2.34E-11 | 310 | 1.00E+06 | 2.43E-11 |
| 320 | 1.00$E$+05 | 2.20E-11 | 320 | 1.00E+06 | 2.29E-11 |
| 330 | 1.00$E$+05 | 2.08E-11 | 330 | 1.00E+06 | 2.18E-11 |
| 340 | 1.00$E$+05 | 1.98E-11 | 340 | 1.00E+06 | 2.08E-11 |
| 350 | 1.00$E$+05 | 1.90E-11 | 350 | 1.00E+06 | 2.00E-11 |
| 360 | 1.00$E$+05 | 1.83E-11 | 360 | 1.00E+06 | 1.94E-11 |
| 370 | 1.00$E$+05 | 1.77E-11 | 370 | 1.00E+06 | 1.88E-11 |
| 380 | 1.00$E$+05 | 1.73E-11 | 380 | 1.00E+06 | 1.84E-11 |
| 390 | 1.00$E$+05 | 1.68E-11 | 390 | 1.00E+06 | 1.80E-11 |
| 400 | 1.00$E$+05 | 1.65E-11 | 400 | 1.00E+06 | 1.77E-11 |
| 410 | 1.00$E$+05 | 1.62E-11 | 410 | 1.00E+06 | 1.75E-11 |
| 420 | 1.00$E$+05 | 1.60E-11 | 420 | 1.00E+06 | 1.73E-11 |
| 430 | 1.00$E$+05 | 1.58E-11 | 430 | 1.00E+06 | 1.72E-11 |
| 440 | 1.00$E$+05 | 1.57E-11 | 440 | 1.00E+06 | 1.71E-11 |
| 450 | 1.00$E$+05 | 1.56E-11 | 450 | 1.00E+06 | 1.71E-11 |
| 460 | 1.00$E$+05 | 1.55E-11 | 460 | 1.00E+06 | 1.70E-11 |
| 470 | 1.00$E$+05 | 1.54E-11 | 470 | 1.00E+06 | 1.70E-11 |
| 480 | 1.00$E$+05 | 1.54E-11 | 480 | 1.00E+06 | 1.71E-11 |
| 490 | 1.00$E$+05 | 1.54E-11 | 490 | 1.00E+06 | 1.71E-11 |
| 500 | 1.00$E$+05 | 1.52E-11 | 500 | 1.00E+06 | 1.72E-11 |
| 510 | 1.00$E$+05 | 1.53E-11 | 510 | 1.00E+06 | 1.73E-11 |
| 520 | 1.00$E$+05 | 1.54E-11 | 520 | 1.00E+06 | 1.75E-11 |
| 530 | 1.00$E$+05 | 1.54E-11 | 530 | 1.00E+06 | 1.76E-11 |
| 540 | 1.00$E$+05 | 1.55E-11 | 540 | 1.00E+06 | 1.78E-11 |
| 550 | 1.00$E$+05 | 1.57E-11 | 550 | 1.00E+06 | 1.80E-11 |
| 560 | 1.00$E$+05 | 1.58E-11 | 560 | 1.00E+06 | 1.82E-11 |
| 570 | 1.00$E$+05 | 1.59E-11 | 570 | 1.00E+06 | 1.84E-11 |
| 580 | 1.00$E$+05 | 1.61E-11 | 580 | 1.00E+06 | 1.86E-11 |
| 590 | 1.00$E$+05 | 1.63E-11 | 590 | 1.00E+06 | 1.89E-11 |
| 600 | 1.00$E$+05 | 1.64E-11 | 600 | 1.00E+06 | 1.92E-11 |
| 610 | 1.00$E$+05 | 1.66E-11 | 610 | 1.00E+06 | 1.94E-11 |
| 620 | 1.00$E$+05 | 1.69E-11 | 620 | 1.00E+06 | 1.97E-11 |
| 630 | 1.00$E$+05 | 1.71E-11 | 630 | 1.00E+06 | 2.00E-11 |
| 640 | 1.00$E$+05 | 1.73E-11 | 640 | 1.00E+06 | 2.04E-11 |
| 650 | 1.00$E$+05 | 1.75E-11 | 650 | 1.00E+06 | 2.07E-11 |
| 660 | 1.00$E$+05 | 1.78E-11 | 660 | 1.00E+06 | 2.11E-11 |
| 670 | 1.00$E$+05 | 1.76E-11 | 670 | 1.00E+06 | 2.14E-11 |
| 680 | 1.00$E$+05 | 1.79E-11 | 680 | 1.00E+06 | 2.17E-11 |
| 690 | 1.00$E$+05 | 1.82E-11 | 690 | 1.00E+06 | 2.21E-11 |
| 700 | 1.00$E$+05 | 1.85E-11 | 700 | 1.00E+06 | 2.25E-11 |
| 710 | 1.00$E$+05 | 1.88E-11 | 710 | 1.00E+06 | 2.30E-11 |
| 720 | 1.00$E$+05 | 1.91E-11 | 720 | 1.00E+06 | 2.34E-11 |
| 730 | 1.00$E$+05 | 1.94E-11 | 730 | 1.00E+06 | 2.38E-11 |
| 740 | 1.00$E$+05 | 1.98E-11 | 740 | 1.00E+06 | 2.43E-11 |
| 750 | 1.00$E$+05 | 2.01E-11 | 750 | 1.00E+06 | 2.47E-11 |
| 760 | 1.00$E$+05 | 2.05E-11 | 760 | 1.00E+06 | 2.52E-11 |
| 770 | 1.00$E$+05 | 2.09E-11 | 770 | 1.00E+06 | 2.57E-11 |
| 780 | 1.00$E$+05 | 2.12E-11 | 780 | 1.00E+06 | 2.62E-11 |
| 790 | 1.00$E$+05 | 2.16E-11 | 790 | 1.00E+06 | 2.67E-11 |
| 800 | 1.00$E$+05 | 2.20E-11 | 800 | 1.00$E$+06 | 2.73E-11 |

**Table S7**. Pressure dependent rate constants calculated at the UM06-2X/aug-cc-pVTZ for R1 pathway.

| Excited state number | CR1 | | | CR2 | | | CR3 | | |
| --- | --- | --- | --- | --- | --- | --- | --- | --- | --- |
|  | E_v_ | λ | *f* | E_v_ | λ | *f* | E_v_ | λ | *f* |
| 1 | 1.19 | 1045.21 | 0.0002 | 0.08 | 15851.76 | 0.0000 | 0.05 | 26995.27 | 0.0000 |
| 2 | 4.05 | 306.19 | 0.2091 | 2.79 | 444.62 | 0.0016 | 3.02 | 410.43 | 0.0034 |
| 3 | 4.38 | 283.2 | 0.0001 | 4.32 | 287.27 | 0.0016 | 3.56 | 348.71 | 0.0007 |
| 4 | 4.96 | 250.15 | 0.0040 | 5.19 | 238.79 | 0.0000 | 4.93 | 251.54 | 0.0000 |
| 5 | 5.25 | 236.16 | 0.321 | 5.47 | 226.72 | 0.0048 | 5.16 | 240.18 | 0.0066 |
| 6 | 5.79 | 214.07 | 0.0181 | 5.84 | 212.27 | 0.0000 | 5.47 | 226.61 | 0.0002 |
| Excited state number | Cp1 | | | Cp2 | | | Cp3 | | |
|  | E_v_ | λ | *f* | E_v_ | λ | *f* | E_v_ | λ | *f* |
| 1 | 0.19 | 6450.70 | 0.0000 | 3.73 | 331.95 | 0.0025 | 3.46 | 358.62 | 0.0001 |
| 2 | 3.51 | 352.91 | 0.0021 | 4.40 | 281.89 | 0.0180 | 4.36 | 284.35 | 0.0020 |
| 3 | 5.37 | 231.06 | 0.0006 | 4.48 | 276.71 | 0.0575 | 5.15 | 240.97 | 0.0075 |
| 4 | 5.61 | 221.05 | 0.0053 | 4.74 | 261.40 | 0.0063 | 5.34 | 232.37 | 0.0004 |
| 5 | 5.79 | 214.00 | 0.0203 | 4.84 | 256.36 | 0.0014 | 5.51 | 224.91 | 0.0326 |
| 6 | 6.12 | 202.47 | 0.0003 | 5.13 | 241.51 | 0.0036 | 5.72 | 216.76 | 0.0064 |
| Excited state number | Cp4 | | | Cp5 | | | Cp6 | | |
|  | E_v_ | λ | f | E_v_ | λ | f | E_v_ | λ | f |
| 1 | 0.21 | 5979.73 | 0.0000 | -0.13 | -9682.82 | -0.0000 | 1.61 | 771.16 | 0.0002 |
| 2 | 2.82 | 438.93 | 0.0014 | 3.63 | 341.26 | 0.0020 | 4.69 | 264.13 | 0.0001 |
| 3 | 3.58 | 346.74 | 0.0057 | 3.99 | 311.07 | 0.0021 | 4.99 | 248.63 | 0.0007 |
| 4 | 3.65 | 339.48 | 0.0007 | 5.30 | 233.74 | 0.0033 | 5.08 | 244.01 | 0.0429 |
| 5 | 3.77 | 328.52 | 0.0003 | 5.57 | 222.50 | 0.0057 | 5.85 | 211.90 | 0.0065 |
| 6 | 4.35 | 285.35 | 0.0018 | 6.11 | 202.82 | 0.0236 | 5.85 | 211.87 | 0.0003 |
| Excited state number | Cp7 | | |  |  |  |  |  |  |
|  | E_v_ | λ | f |  |  |  |  |  |  |
| 1 | 3.73 | 332.04 | 0.0026 |  |  |  |  |  |  |
| 2 | 4.71 | 263.44 | 0.0139 |  |  |  |  |  |  |
| 3 | 5.09 | 243.42 | 0.0007 |  |  |  |  |  |  |
| 4 | 5.50 | 225.27 | 0.0120 |  |  |  |  |  |  |
| 5 | 5.55 | 223.23 | 0.0007 |  |  |  |  |  |  |
| 6 | 5.85 | 211.88 | 0.0251 |  |  |  |  |  |  |

**Table S8**. Excited state parameters of all complexes in the CH_3_SH + OH reaction computed at the TD-M06-2X/aug-cc-pVTZ level. (The geometries of all complexes obtained at the same level of theory, but the geometry of CP4 obtained at the M06-2X/6-311++g(3df,3pd)). The units of vertical excitation energies (*E_v_*) and wavelength (λ) are eV and nm, respectively.


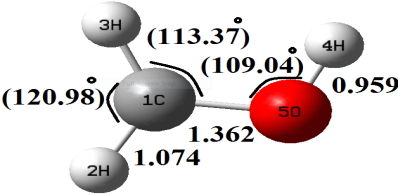

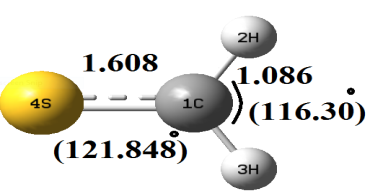

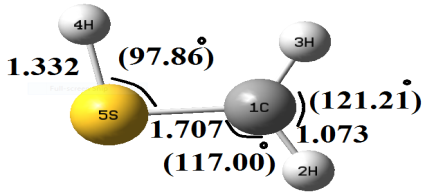


CH_2_OH CH_2_S CH_2_SH


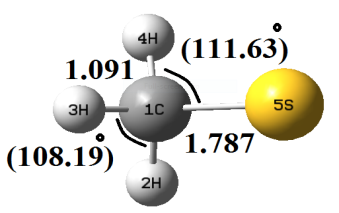

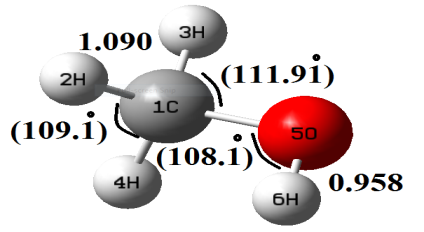

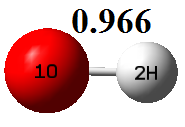


CH_3_S CH_3_OH OH


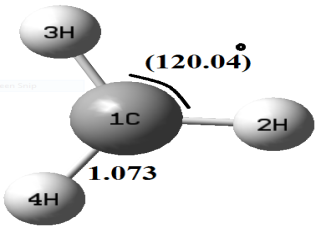

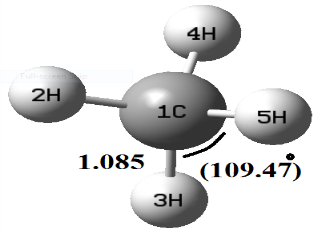

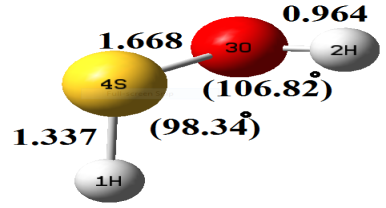


CH_3_ CH_4_ HSOH


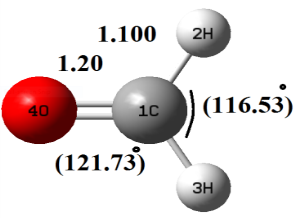

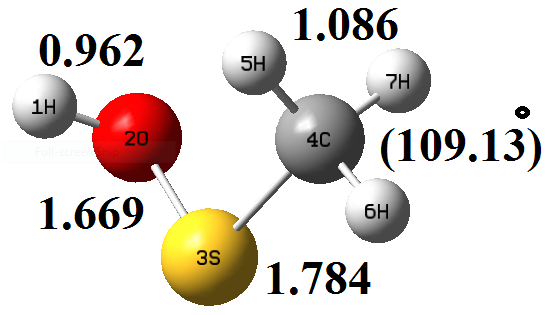

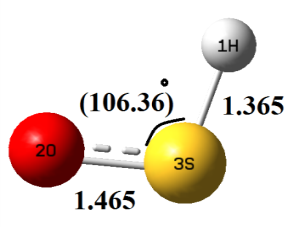


CH_2_O CH_3_SOH HSO

_
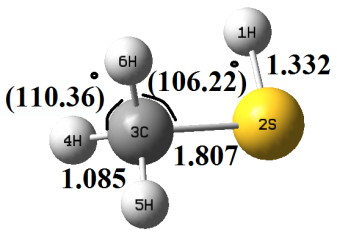

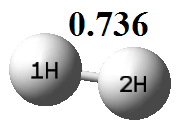

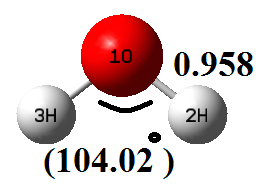
_
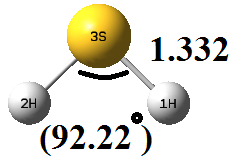

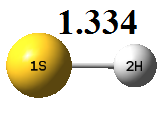


CH_3_SH H_2_ H_2_O SH_2_ SH


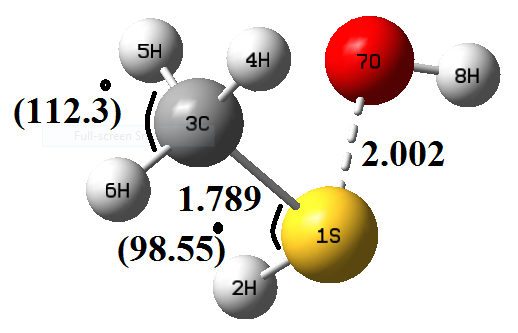

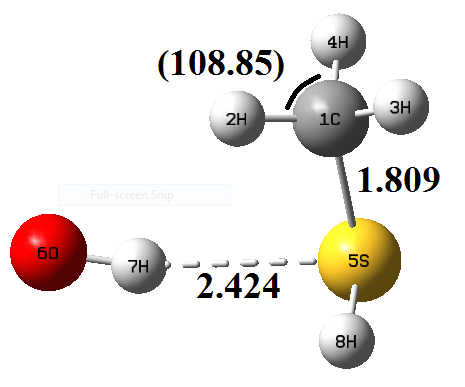

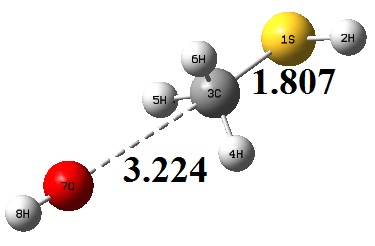


CR 1 CR2 CR 3


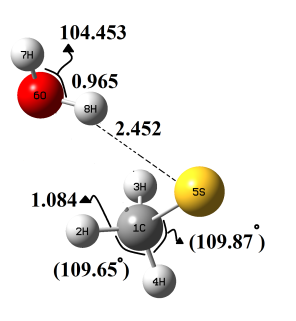

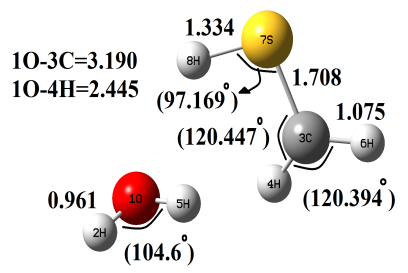

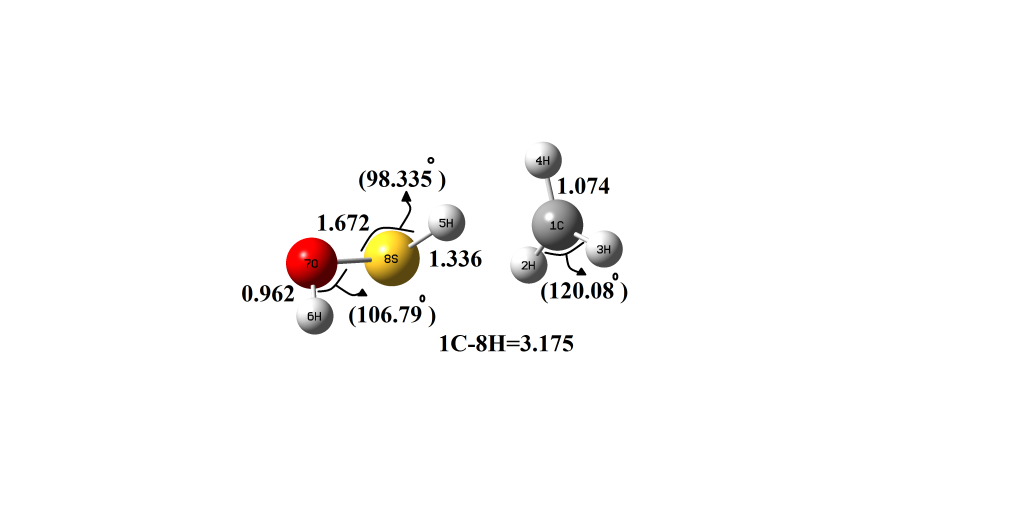


CP2 CP3 CP4


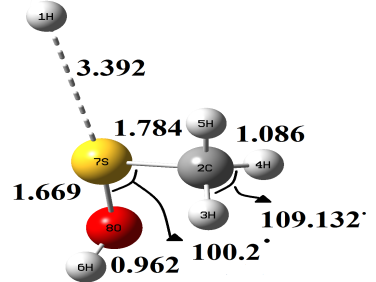

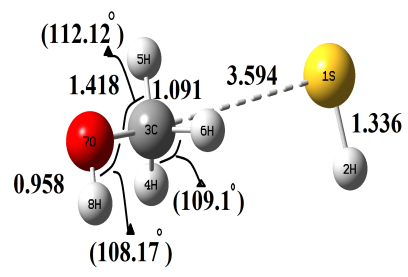

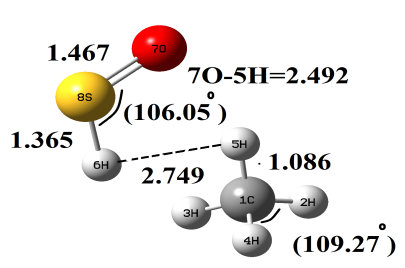


CP4 CP5 CP6


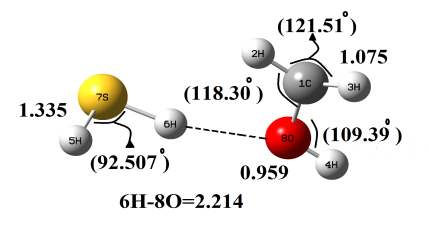

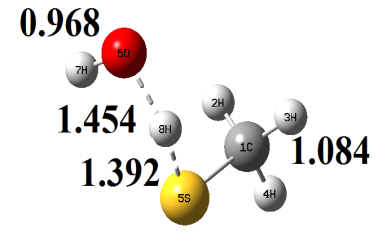

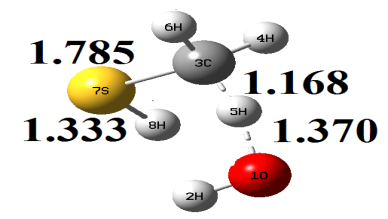


CP7 TS1 TS2


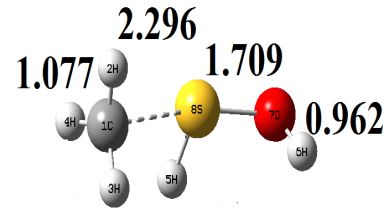

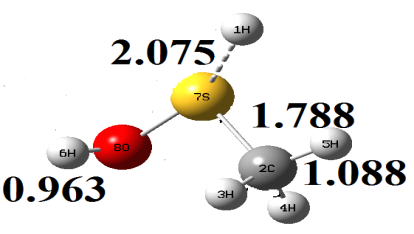

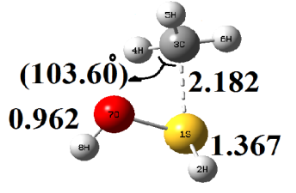


TS3 TS4 TS5


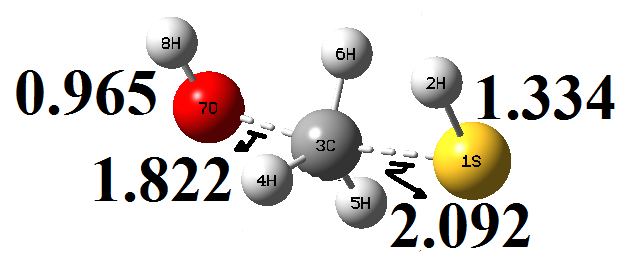

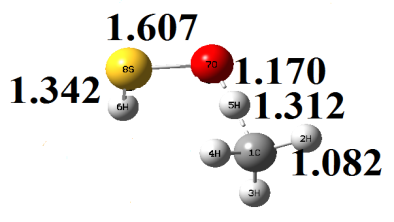

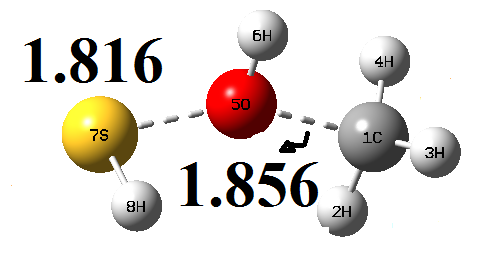


TS6 TS7 TS8
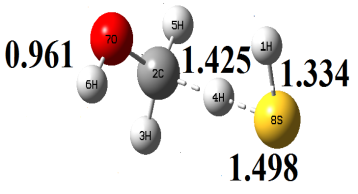

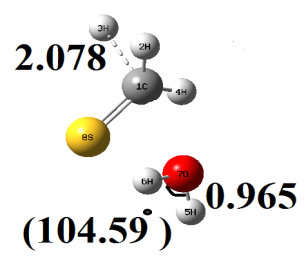


TS9 TS10

**Figure S1**. Geometrical parameters of all species in the CH_3_SH + OH reaction calculated at the MP2/6-311++g(3df,3pd) level of theory (Bond lengths are in angstrom and angles are in degree).


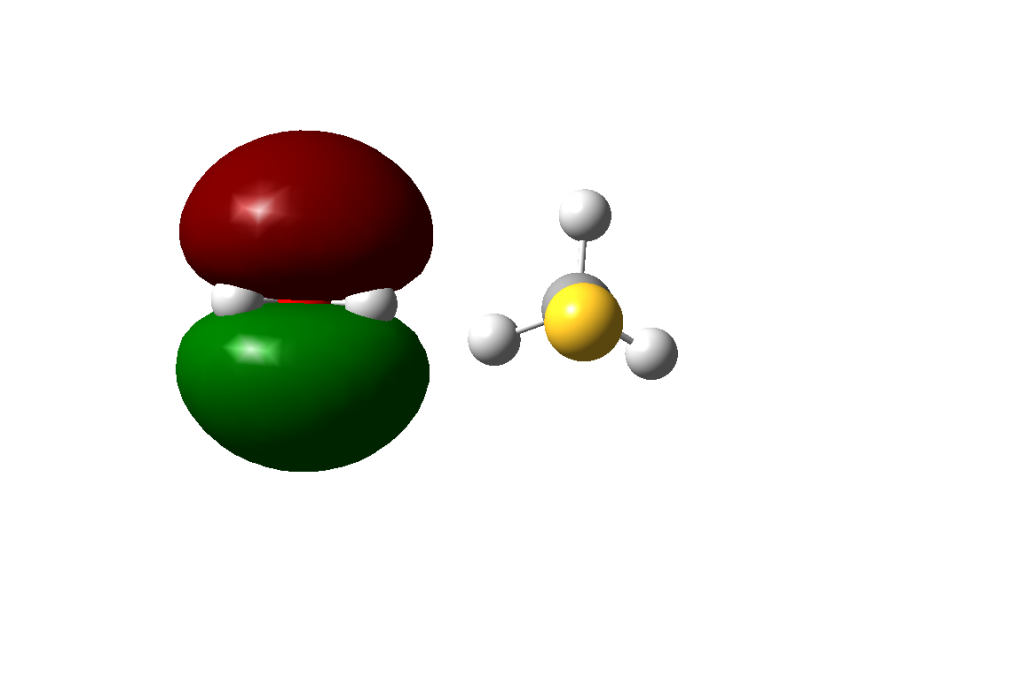

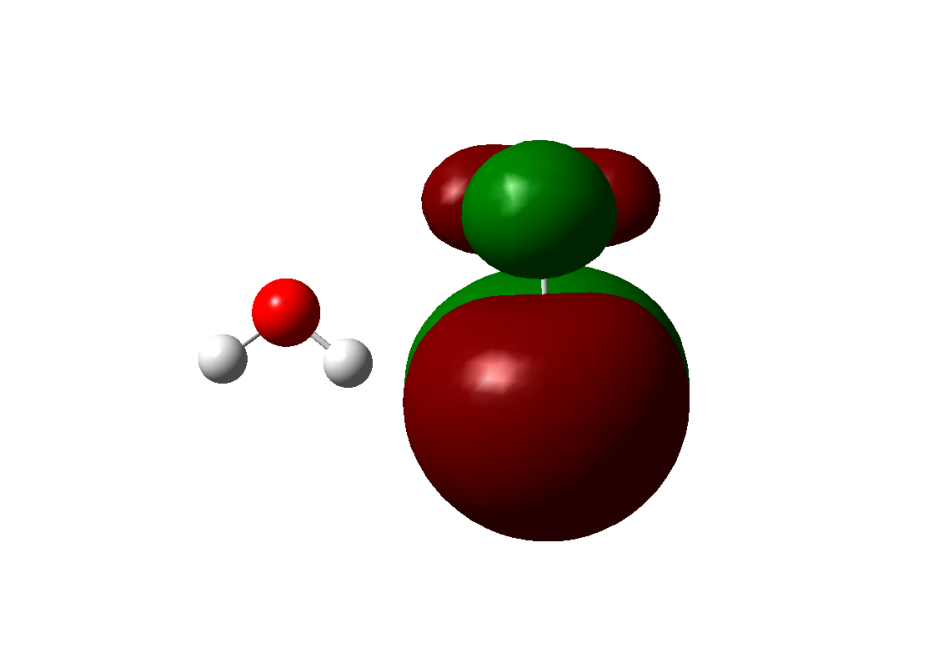


CP1-16B CP1-18B

Second excited sate


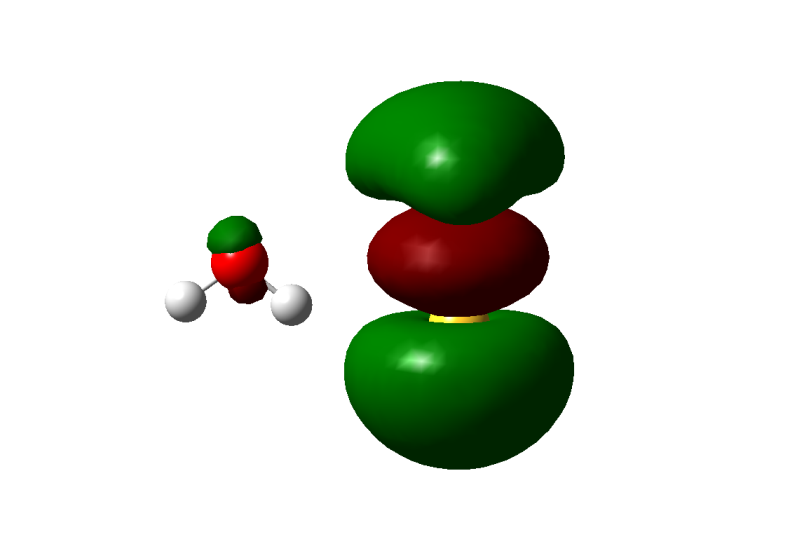

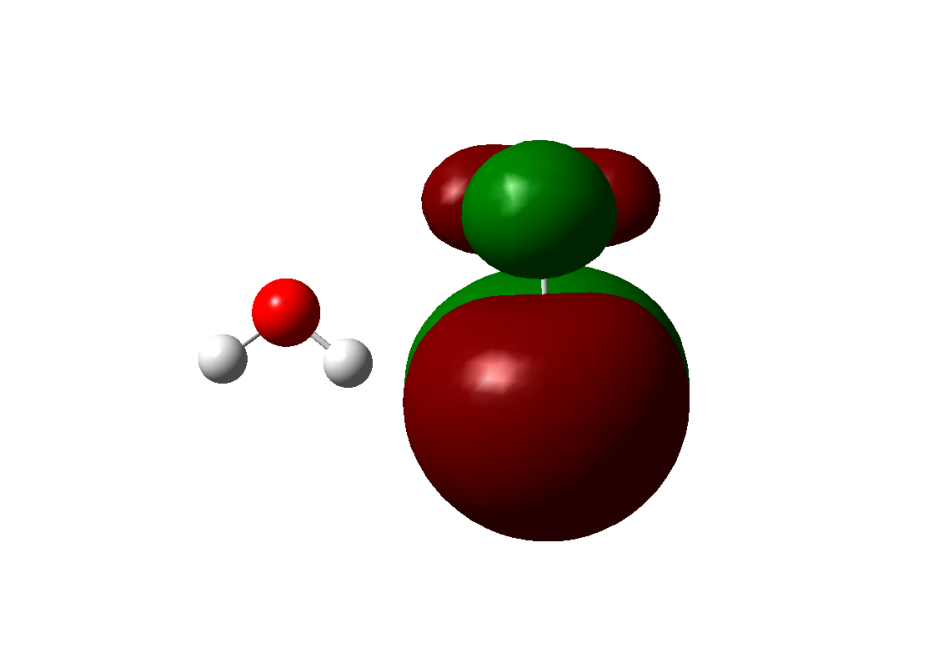


CP1-15B CP1-18B

Third excited state

**Figure S2.** Ground state and the second and third excited state orbitals of CP1.


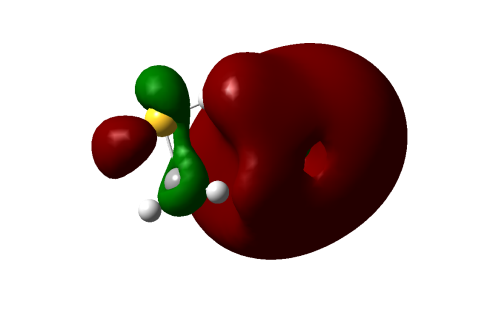


CP2-19A


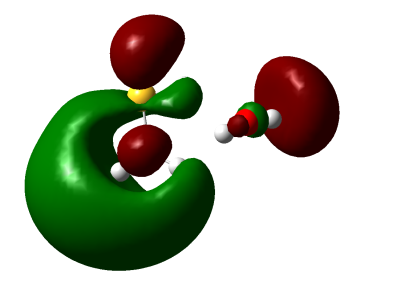


CP2-20A


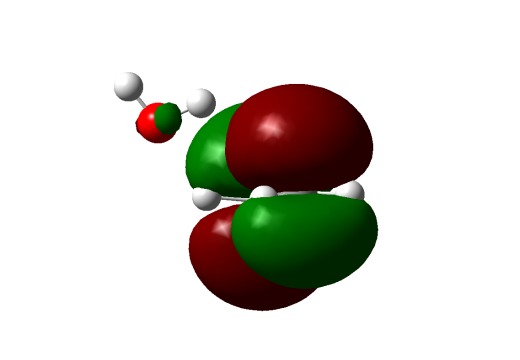

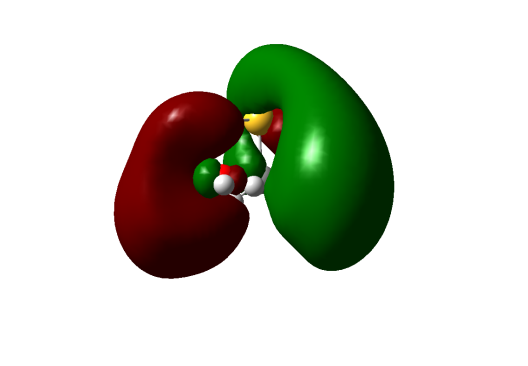


CP2-18A CP2-22A


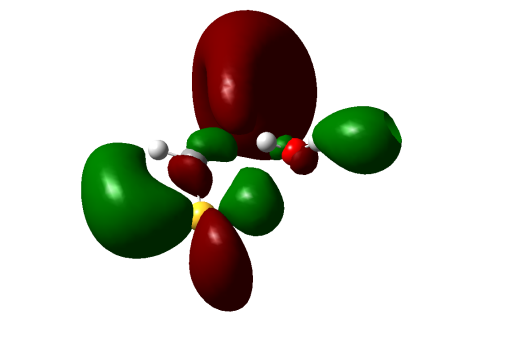


CP2-23A


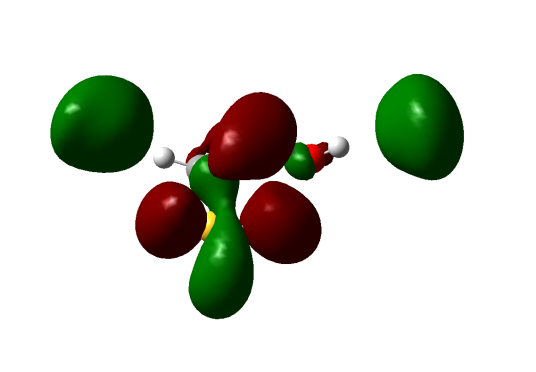


CP2-24A

**Figure S3.** Ground state and the first excited state orbitals of CP2.


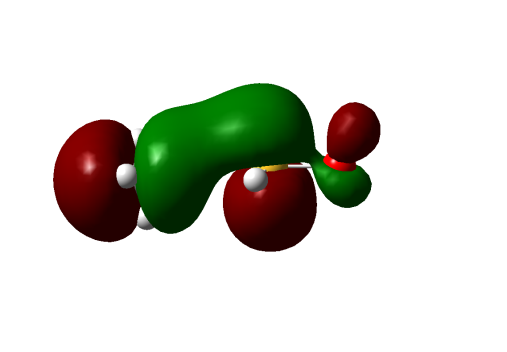

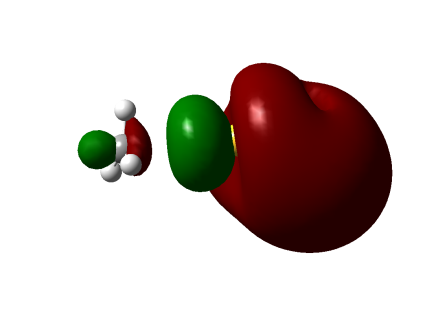


CP3-17A CP3-19A


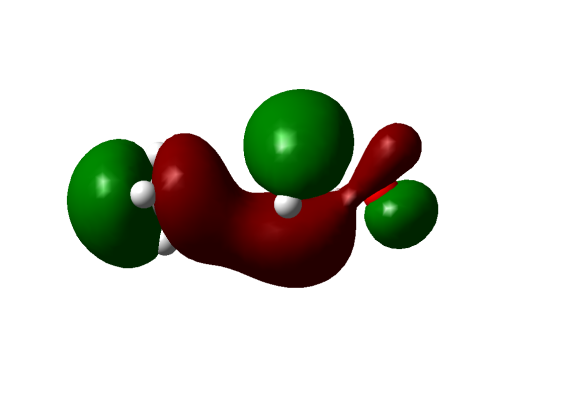


CP3-18A
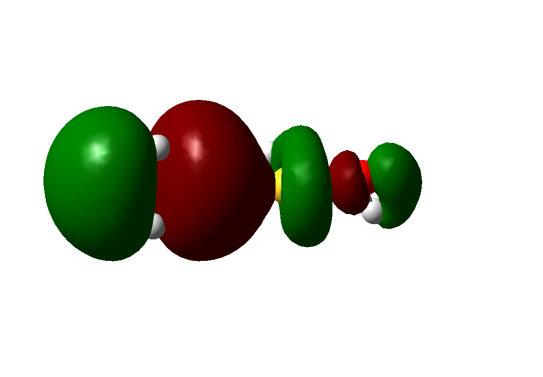


CP3-18B
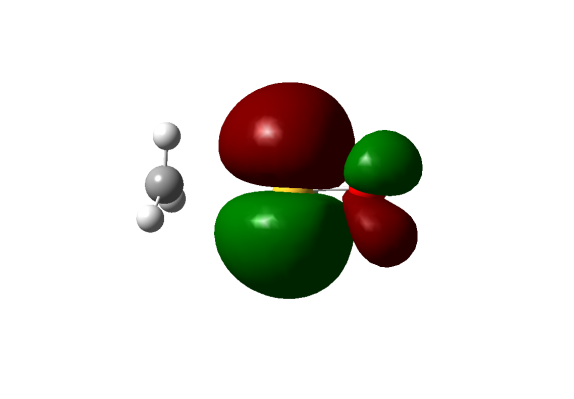

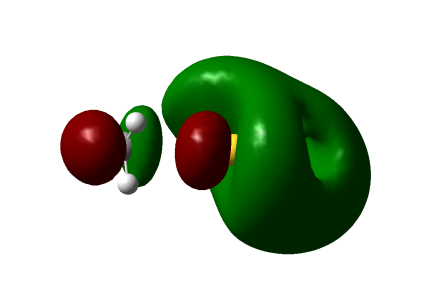


CP3-17B CP3-19B


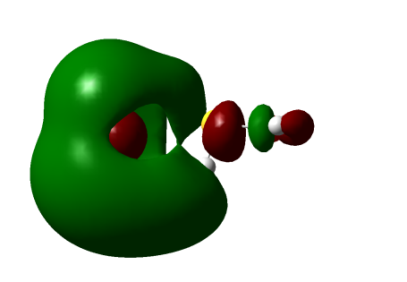


CP3-20B


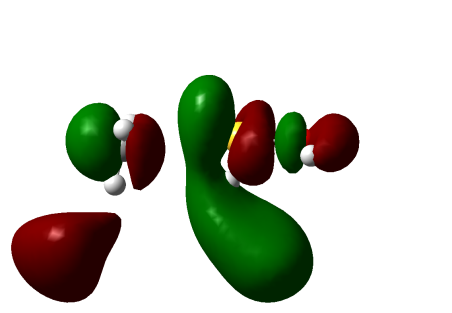


CP3-24B

**Figure S4.** Ground state and the first excited state orbitals of CP3.


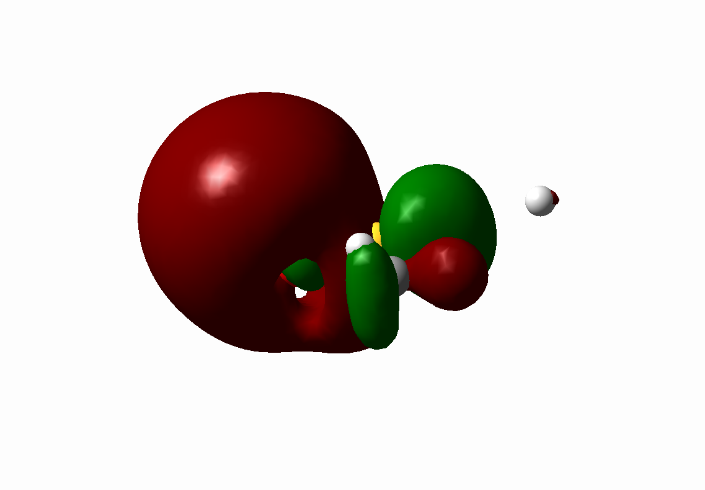


CP4-19A


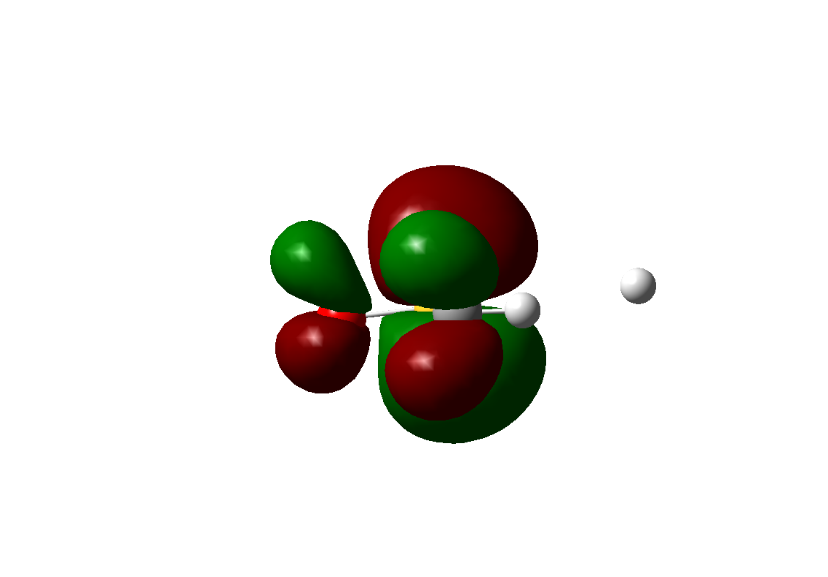

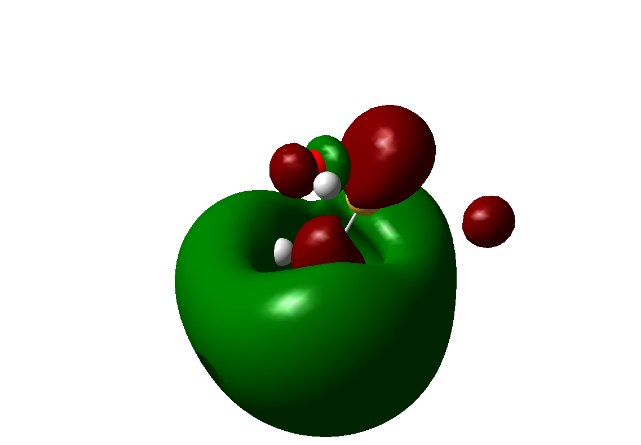


CP4-18A CP4-20A


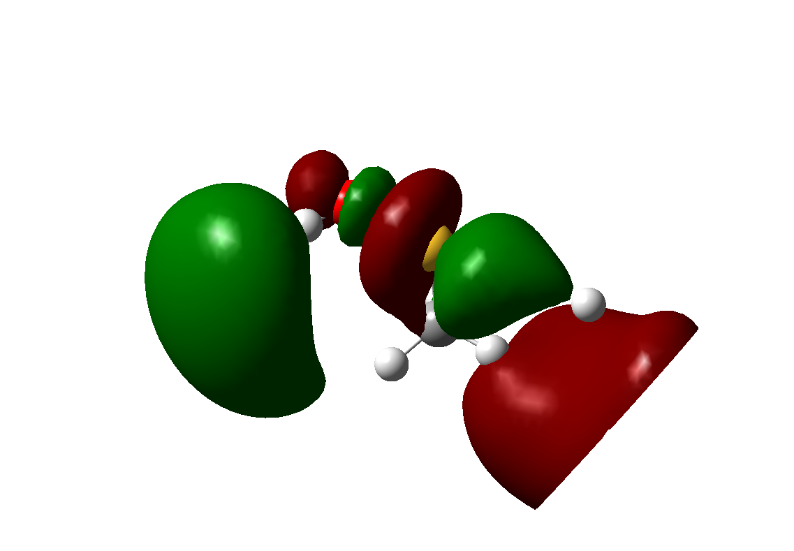


CP4-21A


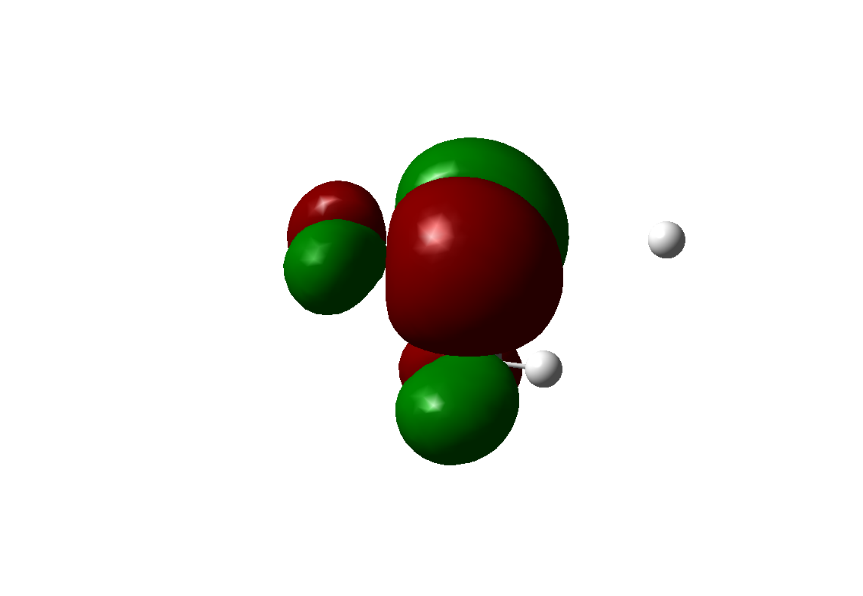

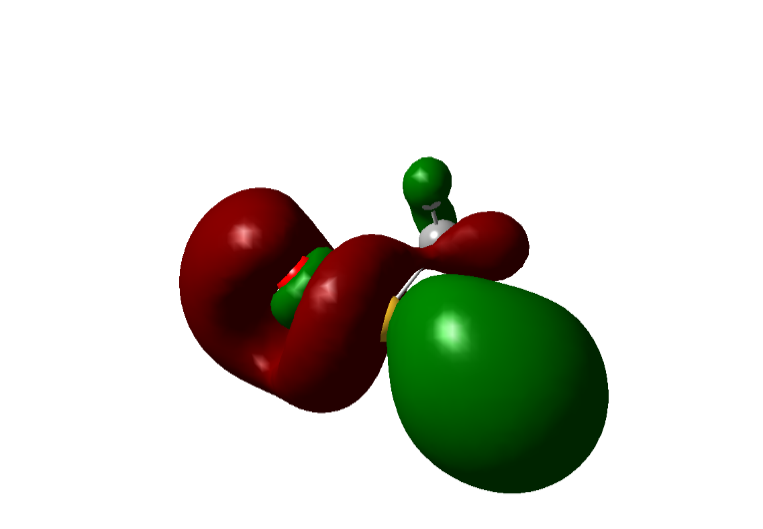


CP4-17B CP4-18B

**Figure S5.** Ground state and the second excited state orbitals of CP4.


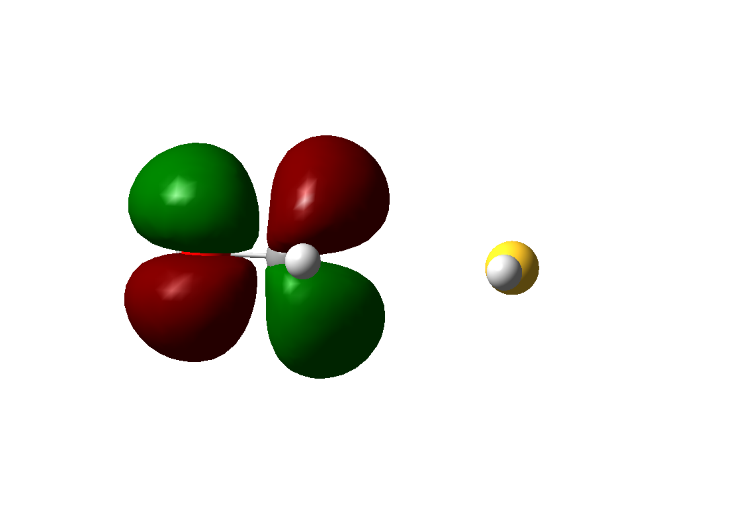

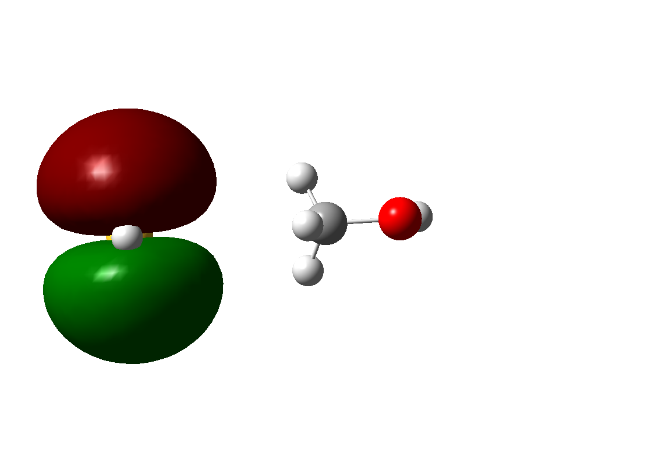


CP5-16B CP5-18B

The second excited state


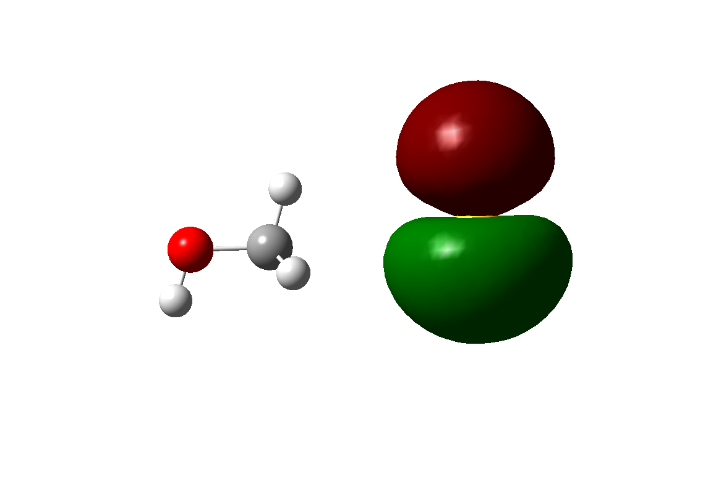


CP5-14B


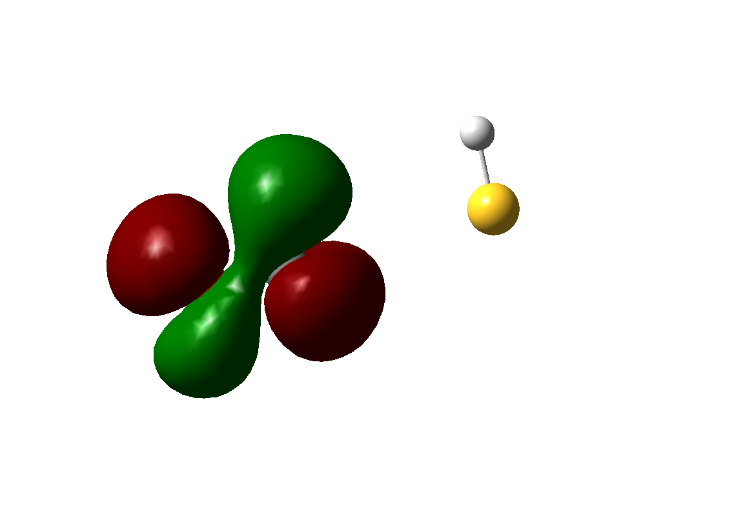

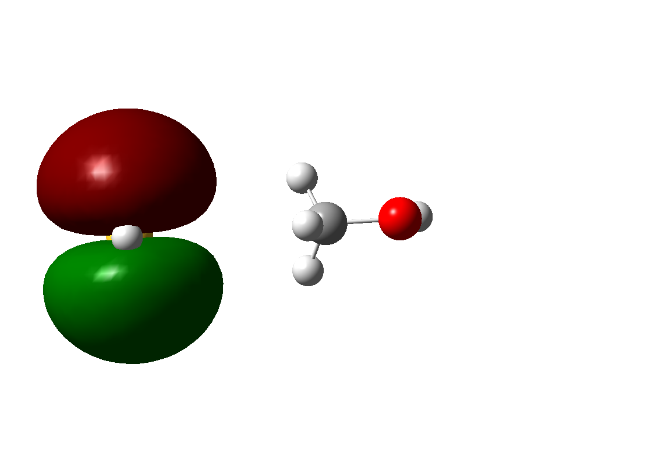


CP5-15B CP5-18B

The third excitrd state

**Figure S6.** Ground state and the second and third excited state orbitals of CP5.


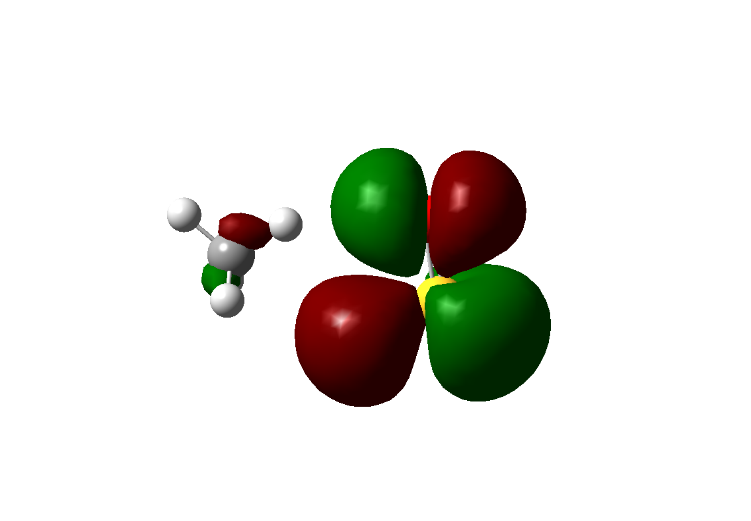

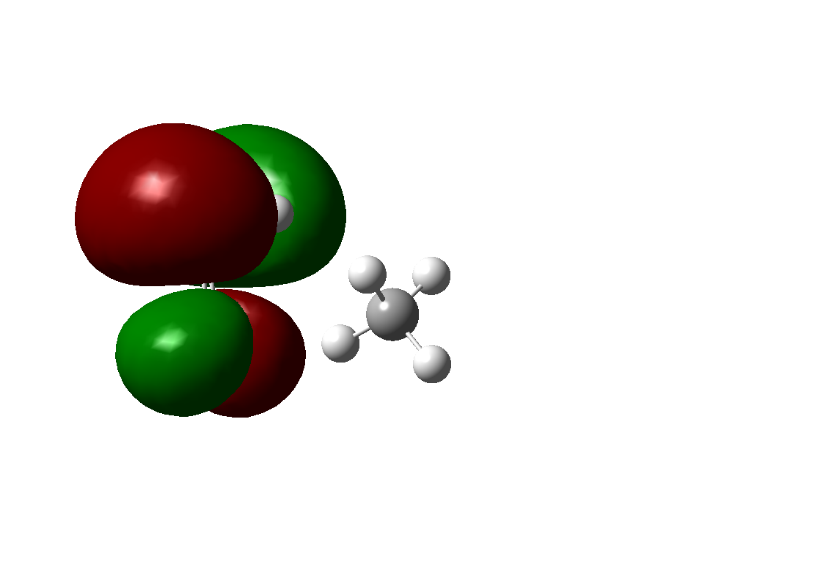


CP6-17B CP5-18B

**Figure S7.** Ground state and the first excited state orbitals of CP6.


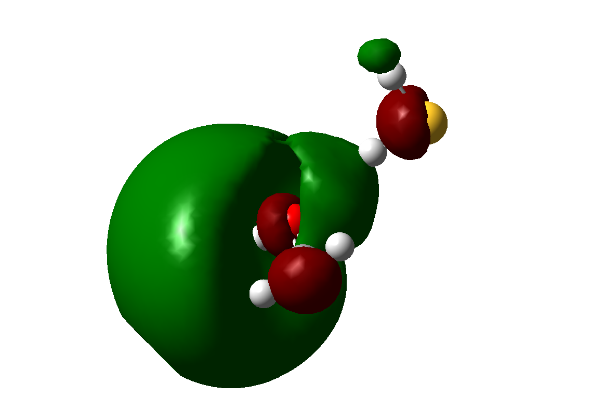


CP7-19A


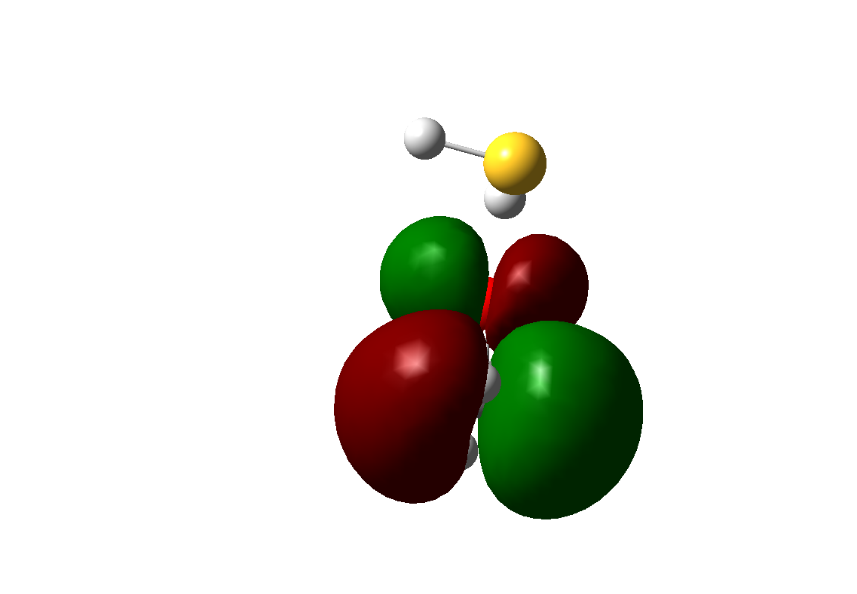

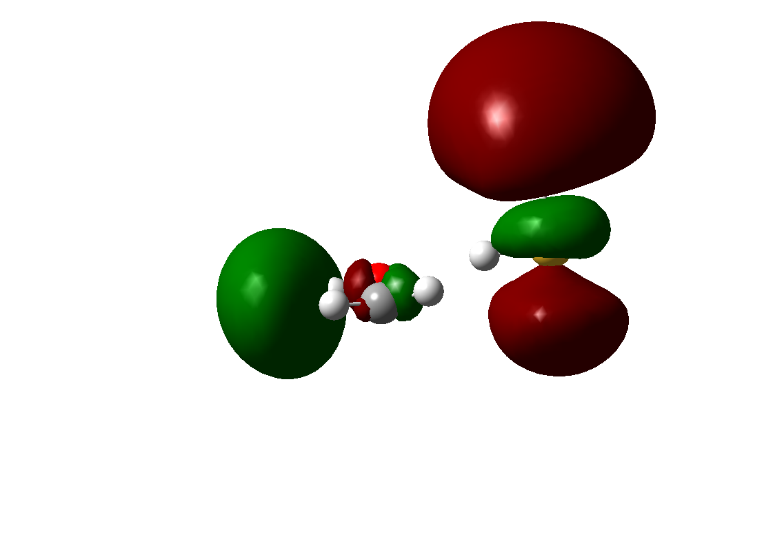


CP7-18A CP7-20A


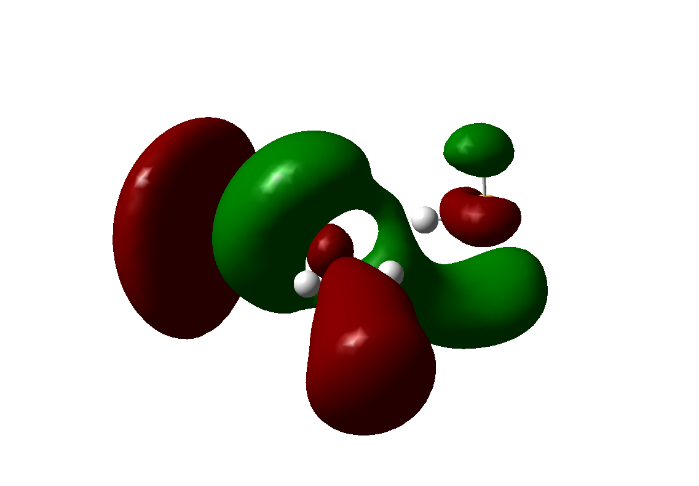


CP7-26A

**Figure S8.** Ground state and the first excited state orbitals of CP7.
